# Mapping the global distribution of *Strongyloides stercoralis* and hookworms by ecological niche modeling

**DOI:** 10.1186/s13071-022-05284-w

**Published:** 2022-06-08

**Authors:** Pedro Emanuel Fleitas, Sebastián Dario Kehl, Walter Lopez, Marina Travacio, Elvia Nieves, José Fernando Gil, Rubén Oscar Cimino, Alejandro Javier Krolewiecki

**Affiliations:** 1 Instituto de Investigaciones de Enfermedades Tropicales (IIET), Sede Regional Orán Universidad Nacional de Salta, Salta, Argentina; 2 Cátedra de Química Biológica, Facultad de Ciencias Naturales, Universidad Nacional de Salta, Salta, Argentina; 3 Consejo Nacional de Investigaciones Científicas y Técnicas (CONICET)–CCT Salta, Salta, Argentina; 4 Instituto Nacional de Enfermedades Infecciosas, Administración Nacional de Laboratorios e Institutos de Salud “Dr. C. Malbrán”, Buenos Aires, Argentina; 5 Facultad de Farmacia y Bioquímica, Cátedra de Química General e Inorgánica, Universidad de Buenos Aires, Buenos Aires, Argentina; 6 Instituto de Investigaciones en Energía No Convencional–CONICET, Salta, Argentina

**Keywords:** *Strongyloides stercoralis*, Hookworms, Ecological niche models, Environmental variables, Population at risk

## Abstract

**Background:**

The WHO has established a control strategy for *Strongyloides stercoralis* in school-aged children as well as targets and to maintain control programs for *Ascaris lumbricoides, Trichuris trichiura* and hookworms. For an efficient development of control programs, it is necessary to know the target countries around the world, as well as the areas within each country where efforts should be focused. Therefore, maps that provide information on the areas at risk for soil-transmitted helminth (STH) infections on a national and sub-national scale would allow for a better allocation of resources.

**Methods:**

We used the ecological niche models MaxEnt and Kuenm R library to estimate the global distribution of *S. stercoralis* and hookworms. We used occurrence points of both species extracted from surveys of two literature reviews and from the Global Atlas of Helminth Infection database, together with 14 raster maps of environmental variables.

**Results:**

We obtained two raster maps with the presence probability of *S. stercoralis* and hookworm infections at a global level and then estimated the global population at risk to be 2.6 and 3.4 billion, respectively. The population at risk was also estimated at the country level using estimations for areas as small as 25 km^2^. A relationship was found between the probability of the presence of *S. stercoralis* and its prevalence, and a raster map was generated. Annual precipitation, annual temperature, soil carbon content and land cover were the main associated environmental variables. The ecological niches of *Strongyloides stercoralis* and hookworms had an overlap of 68%.

**Conclusions:**

Here we provide information that can be used for developing more efficient and integrated control strategies for *S. stercoralis* and hookworm infections. This information can be annexed to the study of other risk factors or even other diseases to assess the health status of a community.

**Graphical Abstarct:**

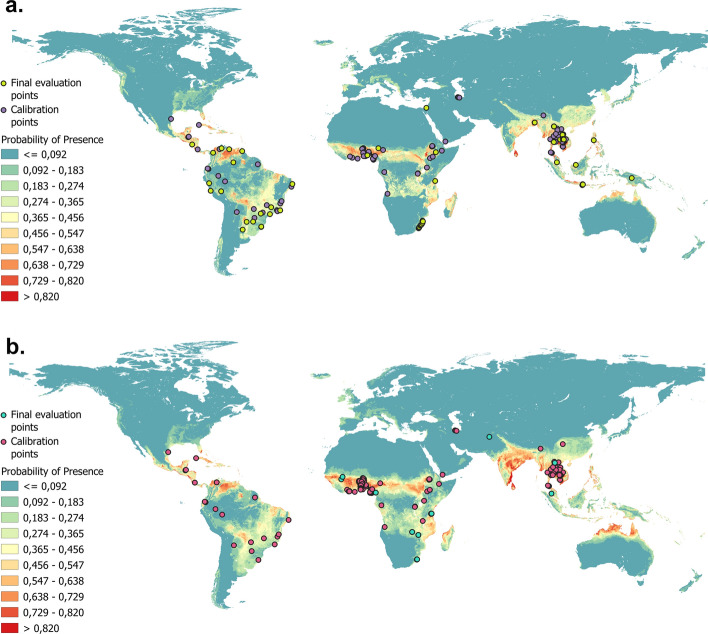

**Supplementary Information:**

The online version contains supplementary material available at 10.1186/s13071-022-05284-w.

## Background

Since 2001 the WHO has promoted preventive chemotherapy (PC) as a strategy to reduce the morbidity of three soil-transmitted helminths (STH), namely *Ascaris lumbricoides*, *Trichuris trichiura* and hookworms [1]. A control strategy for *S. stercoralis* in school-age children (SAC) and targets to maintain control programs for *A. lumbricoides*, *T. trichiura* and hookworms are defined in a recent WHO report [2]. For an efficient development of control programs, it is necessary to identify the target countries in the world, as well as the areas within each country where efforts should be focused. In this context, maps that provide information on the areas at risk for STH infections on a national and sub-national scale would allow for a better allocation of resources. In the last 20 years, a large number of epidemiological studies have been carried out to measure the prevalence of STH infections in different parts of the world [3, 4]. However, precise geographic information on the global distribution of STHs is incomplete for *A. lumbricoides*, *T. trichiura* and hookworms, and almost non-existent for *S. stercoralis*. Therefore, a clear understanding of the spatial distribution and environmental variables associated with disease epidemiology is required to successfully develop prevention and control programs [5].

The distribution of each species is restricted by environmental and biological factors. Thus, data-driving techniques, such as ecological niche models (ENMs), are used to estimate the potential distribution of the species under study, measuring the area suitability for its occurrence and estimating dispersion patterns [6–8]. This potential distribution is calculated using an algorithm with environmental variables and geographic presence/absence points as predictors. However, although data on the presence of STHs in different parts of the world are available, data on the absence of occurrence are scarce and also compromised as such data are affected by the low sensitivity of the diagnostic methods. Therefore, in such cases, algorithms that use only presence data are more suitable. The maximum entropy algorithm (MaxEnt) is frequently used for this purpose due to its high performance using only presence data [8, 9]. To estimate the potential distribution of a species, MaxEnt calculates the probability densities to presence points, which describe the relative likelihood of all environment variables in the model over the range of those points, and the probability density of background points which characterize the environment where a species has been found. MaxEnt measures the ratio between these two probability densities, which gives the relative environmental suitability for the presence of a species for each point in the study area [10, 11] and allows suitability to be transferred between areas by extrapolation, clamp extrapolation or no extrapolation [7, 12]. However, there are some limitations to choosing the presence-only data strategy. The low detectability of organisms and the sampling bias can define areas as being absent of the species, thereby discarding areas where the species can settle. Fortunately, MaxEnt uses a background value (pseudo-absence) for where the species has not been observed and thus this area is not treated as a real absence [11].

In this study, we focus on two species of STHs, hookworms and *S. stercoralis*. These species share biological characteristics and are correlated in their distribution and prevalence [3]. The main aims of this study were: (i) determine, at a global level, the areas that have the highest environmental suitability for *S. stercoralis* and hookworms by modeling raster maps; (ii) determine the population at risk of becoming infected with *S. stercoralis* or hookworm in all countries of the world, identify potential areas where surveys have never been conducted and show environmental suitability for *S. stercoralis* and hookworms; (iii) compare the most suitable areas with the reported prevalence data; (iv) determine the main environmental variables associated with presence of *S. stercoralis* and hookworm infections; and (v) determine the degree of overlap between the ecological niches of *S. stercoralis* and hookworms.

## Methods

### Occurrence data of *S. stercoralis* and hookworms

Only data from epidemiological surveys prior to the intervention (with prevalence other than zero) were used to model the environmental suitability of the species, in order to identify the environmental suitability without the pressure of massive deworming campaigns. Temporal variations in prevalence were not considered because this type of model seeks to model the environmental suitability of the species, not its prevalence, and the only relevant issue is whether the species is present or absent. The geographic presence points for the ecological niche modeling of *S. stercoralis* and hookworms were extracted from a systematic review that reported prevalence for both for the period ranging from 2001 to 2018 (PROSPERO registration code: CRD42019131127) [3]. All surveys that correctly reported the geographic location were incorporated into the present study, and those with ambiguous geographic location were discarded. To reduce the space autocorrelation, we retained unique locations within a vicinity of 10 km^2^ using the package ‘ecospat’ of R software [13, 14]. Based on this information, for the calibration of the niche model of *S. stercoralis* and hookworms, we used 104 and 156 presence points obtained from 69 and 70 papers, respectively, from the systematic review (Additional file 1: Table S1; Additional file 2: Table S2).

For the final evaluation of the *S. stercoralis* niche model, we used a literature review of the global prevalence of *S. stercoralis* [15]; the papers that were already included in the systematic review and those that presented ambiguous geographic location were discarded. On the other hand, for the final evaluation of the hookworm niche model, surveys from the Global Atlas of Helminth Infection (GAHI) [16] were used. Pre-intervention surveys from 2001 to 2015 were selected. Therefore, 56 independent presence points obtained from the literature review (from 41 papers) were used for the final evaluation of the *S. stercoralis* models [15]; and 62 independent presence points obtained from GAHI were used for the final evaluation of the hookworm models (Additional file 1: Table S1; Additional file 2: Table S2).

### Environmental variables

A total of 27 environmental variables were analyzed to determine the ENM for both species. We used a set of 19 climatic variables from the WorldClime Project derived from spatial interpolation of temperature and precipitation [17–19], elevation [19], soil pH [20], maximum soil moisture [20], aridity index [20], organic carbon of soil (OC) [21], soil nitrogen [21], soil classes (118 unique soil classes) [20, 22] and land cover (global land cover by National Mapping Organizations, which is geospatial information in raster format that classifies the status of land cover of the whole globe into 20 categories [23]). All variables were resampled to a spatial resolution of 25 km^2^ with an extension that covers most inhabited land surface using the ‘dismo’ and ‘raster’ packages of the R software package [24, 25]. Finally, of the 27 environmental variables, 13 were discarded due to their high correlation with and low biological importance for the target species. The remaining 14 variables are were annual mean temperature, annual mean diurnal range, isothermality, temperature seasonality, annual temperature range, annual precipitation, precipitation seasonality, soil nitrogen, soil pH, soil moisture, soil OC, land cover, soil classes and altitude.

### Ecological Niche modeling

The MaxEnt algorithm V.3.4.0 [26] was used to model ecological niches for both species using KuenmMood software V.1.1.6 [12]. MaxEnt uses the environmental and occurrence data to calculate habitat suitability, which is the area where there are certain structural conditions necessary for the survival or reproduction of the species [12].

Modeling in MaxEnt requires determining the mobility area of the species (M). M is the area where the species can explore by dispersion [6]. However, the mobility of STHs is linked to the human host; therefore, the geographic points of the surveys were used to calculate an hypothetical M [27, 28]. The points were grouped into three areas (Americas, Africa and Asia) and six sub-areas (South America, Central America, west Africa, east Africa, west Asia, east Asia). A convex-hull and its central point were calculated for each of these areas. Then, the distance between the most marginal points and the central point was measured. All these steps were performed using the QGIS V.3.16 software package [29]. A nested analysis of variance (ANOVA) was performed to determine whether there was a difference between the M calculated for the different areas and sub-areas. For both species, the nested ANOVA revealed that there were significant differences between the M calculated for South America and the M calculated for the other subcontinents (Additional file 3: Table S3). Therefore, an M was used for South America and a different M was used for the rest of the world. Finally, a buffer was calculated around all of the points with the chosen M. However, the calculation of this M is arbitrary and depends on the availability of survey data in a particular area. To reduce this limitation, since the size of M is critical for the development of an ENM, we constrained the size of M to 50% and 25% of the real measure (100%) (Additional file 3: Table S3). Therefore, the MaxEnt models were calibrated with three Ms for each species (M at 100% [m100%], M at 50% [M50%] and M at 25% [M25%]). In addition, the raster maps of the different variables were adjusted to the size of the different Ms, and a correlation analysis was performed to rule out highly correlated variables.

To model each species, 1479 candidate models for each M (4437 candidate models for each species) were created. These models were performed by combining three sets of environmental variables, 17 values of regularization multiplier (0.1–1.0 at intervals of 0.1, 2–6 at intervals of 1, 8 and 10), and all 29 possible combinations of five feature classes (linear [l], quadratic [q], product [p], threshold [t], and hinge [h]). Candidate model performance was evaluated based on the significance the partial receiver operating characteristic (partial ROC), with 500 iterations and 50% of data for bootstrapping, omission rates (*E* = 5%) and model complexity (Akaike’s information criterion) [12]. Of the models that met the evaluation criteria, the final model was chosen on the basis of the mean area under the curve (AUC) ratio obtained with independent presence points of the calibration. The final model for each species was transferred to a global distribution. The individual response curve from the result of the MaxEnt models was calculated. To determine the type of model output (free extrapolation, extrapolation or clamping), the behavior of the response curve outside the area M was observed for the three variables with the greatest contribution to the final model [30]. All models were created with logistic output, where the final output is the probability of presence (PP), which measures the probability that the species is present, conditional on environmental conditions [31].

### Comparison between the most suitable areas and the reported prevalence

Non-parametric Kruskal–Wallis tests and Dunn’s multiple comparisons test were performed to compare the PP and different prevalence ranges.

### Ecological niche overlap

The degree of overlap between niches was calculated as described previously [32]. Briefly, niche models based on ellipsoids were used, and the degree of overlap was calculated using the Jaccard index, which measures the proportion of points at the intersection of the two ellipsoids. In addition, we verified whether the two ellipsoids presented the same overlapping as random data [32]. For this purpose, the overlap between pairs of ellipsoids created with points randomly sampled from the background of each species was measured. This process was repeated 1000 times and the results compared with the observed value of the overlap. In this context, the null hypothesis is that the two ellipsoids fitted to the actual observations overlap at least as much as the random data ellipsoids [32].

### Population at risk of infection with *S. stercoralis* and hookworms

The population at risk was defined according to the number of people found in areas that are suitable for the development of *S. stercoralis* or hookworm infections.

A mesh of regular points was generated at 25-km^2^ intervals (one point within each pixel of the ENM raster). To know the population and the country of each point, a raster was used with all countries [33], and the population every 1 km was updated to 2020 [34]. Data on longitude, latitude, PP (for each species), country identification and population number were extracted for each point. A comma separated value (CSV) database was generated with this information, which includes over 9 million geographic points (Additional file 4: Total_data).

Low-risk areas were defined as those that presented a PP higher than the third quartile of null prevalence and lower than the median of non-null prevalence. Inversely, high-risk areas were defined as those presenting a PP equal to or greater than the median of non-null prevalence**.**

## Results

### Ecological niche modeling of *S. stercoralis* and hookworm

Of the 4437 candidate models for each species, six models met the evaluation criteria for *S. stercoralis* (two models with M100%, three models with M50% and only one model with M25%), and three for hookworm (two models with M100%, and one with M50%) (Additional file 5: Table S4; Additional file 6: Table S5). The best model for *S. stercoralis* presented a mean AUC ratio of 1.32, where only 5% of the independent points were found in areas with a PP of < 0.05. In comparison, the best hookworm model presented an AUC ratio of 1.59, where 1.6% of the independent points fell in areas with a PP of < 0.05. In both models, the extrapolation of the suitability areas was carried out through free extrapolation because most of the involved variables were based on this behavior. Raster maps of the best models for *S. stercoralis* and hookworms are provided in Additional file 7: asc file S. stercoralis_Niche_Raster_map, and Additional file 8: asc file HKW_Niche_Raster_Map. 

**Fig. 1:**
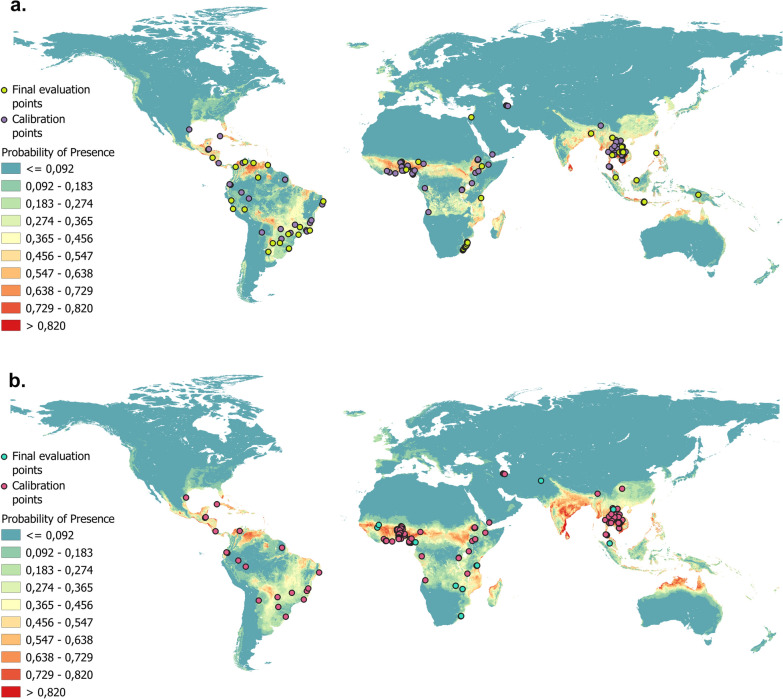
Logistic ecological niche models raster maps for *Strongyloides stercoralis* and hookworms.** a** *S. stercoralis*, **b** hookworms

As expected, the most suitable areas for both species were found in those countries within the tropics (Fig. 1). In addition, from the response curves of each variable provided by MaxEnt, it was determined that the four most relevant variables in the two models were annual precipitation, annual mean temperature, land cover and soil pH (Table 1). However, the percentage of contribution of these variables varied between the models. Although annual temperature is the main variable for both species, annual precipitation is more important for hookworms and land cover is more important for *S. stercoralis*. In addition, the optimal values of the variables are similar in both models with the exception of soil OC. This latter variable has a greater contribution in the *S. stercoralis* model; maximum PP is obtained at < 50 g/kg, and an increase in soil OC produces no increase or decrease in the PP of *S. stercoralis*. However, in the hookworm model, maximum PP is reached as the soil OC increases to levels > 25 g/kg.

**Table 1:** Contribution of each environmental variable to the *Strongyloides stercoralis* hookworm niche model

Variable	Percent contribution of variable to model	Optimal range/main categories
*S. stercoralis niche model*		
Annual precipitation	46.5	1000–1500 mm
Land cover	17.0	1. Urban 2. Paddy field 3. Cropland/other vegetation mosaic
Annual mean temperature	13.8	> 26 ºC
Soil pH	10.5	5.3–6.5
Soil organic carbon	7.2	≤ 50 g/kg
Precipitation seasonality	5	50-100 mm
*Hookworm niche model*		
Annual precipitation	32.2	1000–2000 mm
Annual mean temperature	26.6	26–30 ºC
Land cover	21.8	1. Mangrove 2. Urban 3. Cropland/other vegetation mosaic
Soil pH	10.6	5.5–6.5
Soil organic carbon	3.9	> 25 g/kg
Precipitation seasonality	1.9	60–120 mm
Isothermality	1.7	< 40 ºC
Annual temperature range	1.2	< 14 ºC
Annual mean diurnal range	0.1	< 2 ºC

### Comparison between the probability of presence and the reported prevalence

**Fig. 2:**
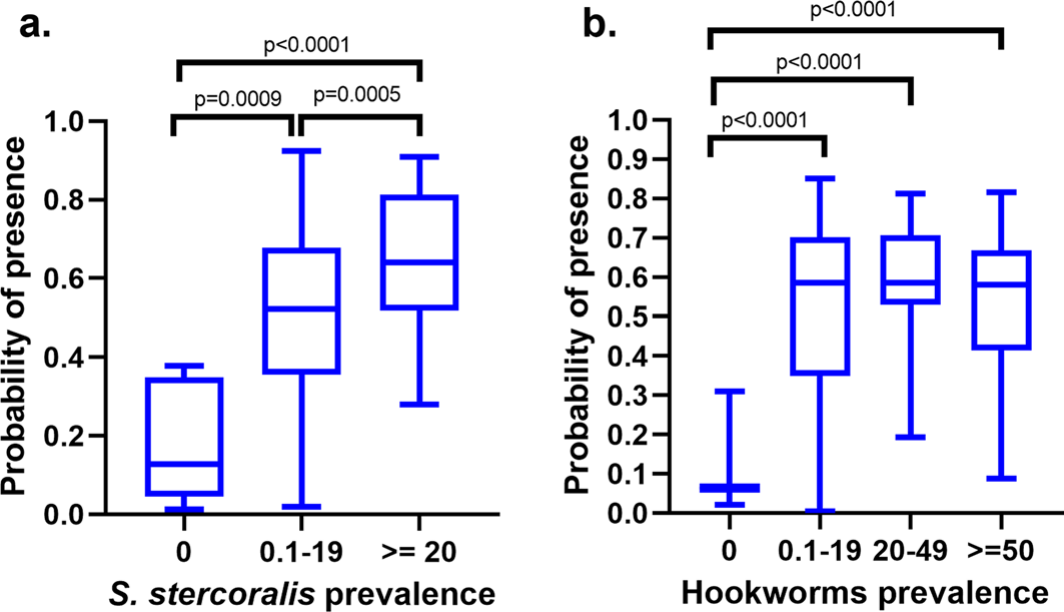
Probability of presence in locations of null, low and high (≥ 20) prevalence. **a** *S. stercoralis***, b** hookworms

*Strongyloides stercoralis* presented differences in PP in places with different prevalence (Fig. 2a). Those locations with a prevalence equal to zero presented a median PP of 0.13 (interquartile range [IQR] 0.35–0.04), and those with low prevalence (1–19%) and high prevalence (≥ 20%) presented a median PP of 0.52 (IQR 0.68–0.36) and 0.64 (IQR 0.81–0.52), respectively. In contrast, for hookworms,, those locations with different prevalence did not report differences in their PP, but differences were observed for locations with reported zero prevalence (Fig. 2b). The locations with zero prevalence present a median PP of 0.06 (IQR 0.07–0.05), while all those locations with non-zero prevalence present a median PP of 0.59 (IQR 0.70–0.41) (Fig. 2b). The scatter plot graphs of the prevalence versus the probability of presence for the two species are provided in Additional file 9: Figure S1.

**Fig. 3:**
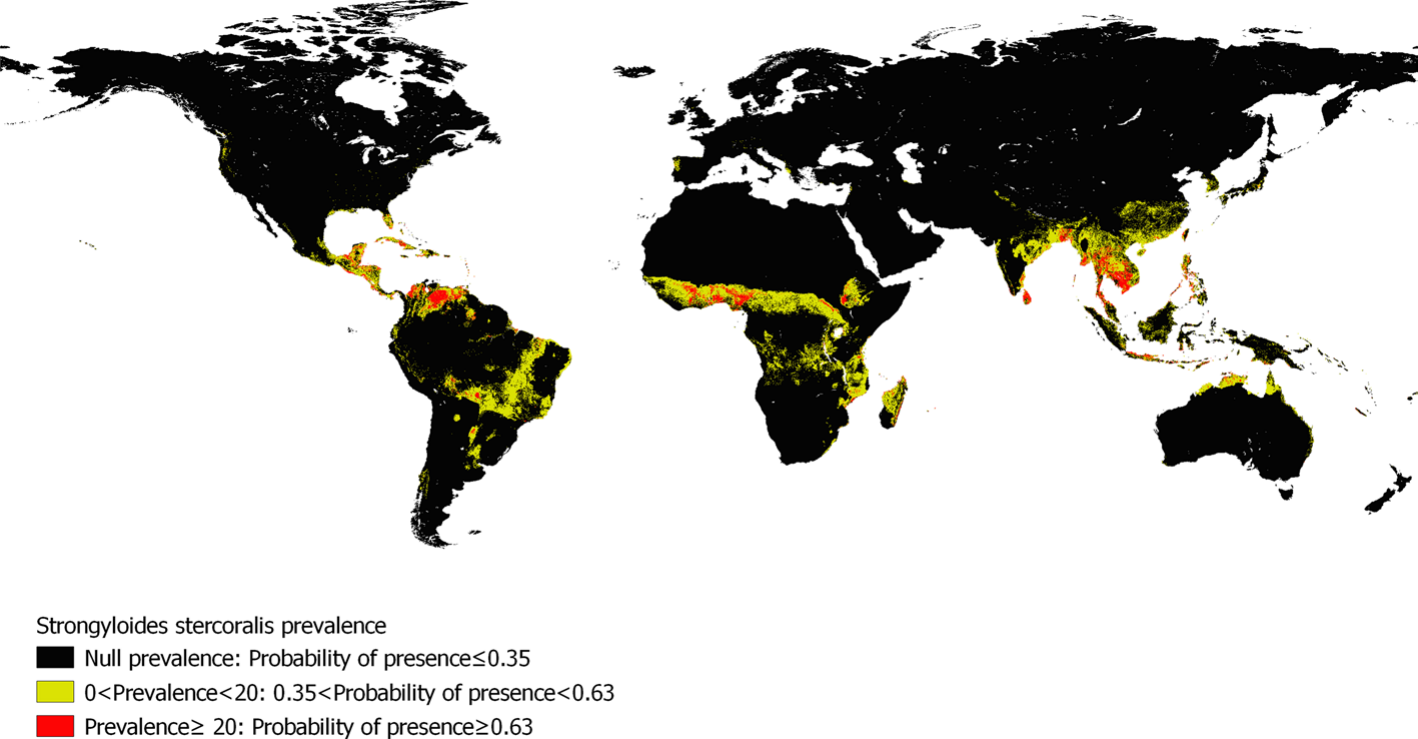
Global map of prevalence of *S. stercoralis*, estimated with the ecological niche model

Figure 2a suggests that there is a relationship between the prevalence of *S. stercoralis* and the PP. Therefore, it is possible to identify, using the PP ranges in Fig. 2a and the PP of Fig. 1a, areas of null, low or high prevalence for *S. stercoralis* (Fig. 3).

### Level of overlap of *S. stercoralis* and hookworm niches

**Fig. 4:**
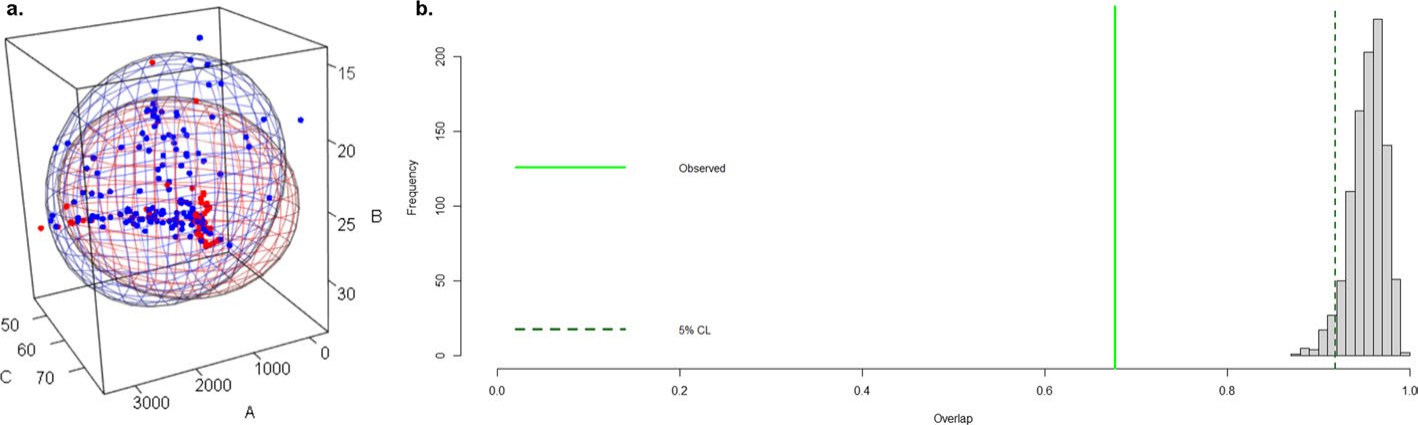
Representation of the overlap of the ecological niches of *S. stercoralis* and hookworms. **a** Niche overlap**.** The blue ellipsoid represents the *S. stercoralis* niche and the red ellipsoid represents the hookworm niche. Blue and red points represent occurrences for *S. stercoralis* and hookworms, respectively.* A* Annual precipitation,* B* annual mean temperature,* C* soil pH. **b** Significance test. The continuous green line shows the overlapping of the *S. stercoralis* and hookworm niches, and the green dotted line shows the overlapping of niches created with random data from the background of each species. Abbreviations: CL, Confidence limit

It was observed that *S. stercoralis* and hookworms share more than half of the environmental conditions (Jaccard index = 0.68), and based on the significance test, we rejected the null hypothesis: that is to say, the ecological niches are different (*p* < 0.01; Fig. 4).

### Population at risk of acquiring *S. stercoralis* or hookworm infections

It was found that the world population at risk of *S. stercoralis* infection is > 2.6 billion persons, while the population at risk of hookworms infection is > 3.4 billion persons (Table 2). (The complete table with all the countries by continent can be seen in Additional file 10: Table S6). These include those who live in areas suitable for the development of *S. stercoralis* or hookworms. Asia has the largest population at risk, with > 1.7 and 2.4 billion persons at risk for *S. stercoralis* and hookworm infection, respectively, with 65% of countries at risk and with countries like India, China, Bangladesh and Indonesia having > 100 million people at risk. Africa hosts more than 510 and 570 million persons at risk for *S. stercoralis* and hookworm infection, with 77% of the countries at risk and with 12 countries with > 10 million at risk: Nigeria, Ethiopia, Democratic Republic of Congo, Tanzania, Kenya, Uganda, Ghana, Ivory Coast, Madagascar, Mozambique, Benin and Cameroon. In third place are the Americas, with > 368 and 375 million at risk for *S. stercoralis* and hookworm infections, with 89% of the countries at risk; Brazil is the country with the highest number of people at risk. In contrast, Europe and Oceania have the lowest population at risk, with > 10 and 8 million persons, respectively. However, those 8 million people represent 27.8% of the entire population of Oceania.

**Table 2:** Risk population of *S. stercoralis* and hookworms

Continent	Country	Population of country (*n*)	Population at low risk for ST infection (*n*)	Population at high risk for ST infection (*n*)	Total population at risk for ST infection (*n*)	Percentage of population at risk for ST infection	Population at low risk for HKW infection (*n*)	Population at high risk for HKW infection (*n*)	Total population at risk for HKW (*n*)	Percentage of population at risk for HKW infection
Asia	India	1,525,704,000	302,142,700	165,507,400	467,650,100	30.7	624,915,700	644,776,800	1,269,692,500	83.2
Asia	China	1,551,020,000	298,324,300	139,983,000	438,307,300	28.3	357,562,200	12,475,960	370,038,160	23.9
Asia	Bangladesh	174,805,500	17,704,140	148,211,800	165,915,940	94.9	92,233,450	82,287,390	174,520,840	99.8
Asia	Indonesia	292,994,400	55,381,560	119,335,800	174,717,360	59.6	128,262,700	40,321,320	168,584,020	57.5
*Total Asia*		*5,066,129,848*	*855,813,064*	*913,900,091*	*1,769,713,155*	*34.9*	*1,427,200,0*31	*1,048,968,20*5	*2,476,168,236*	*48.9*
Africa	Nigeria	234,119,300	37,748,330	92,875,220	130,623,550	55.8	87,618,050	82,008,350	169,626,400	72.5
Africa	Ethiopia	113,451,000	30,060,080	25,832,740	55,892,820	49.3	19,713,430	964,709	20,678,139	18.2
Africa	Democratic Republic of Congo	123,728,800	41,440,290	7,917,517	49,357,807	39.9	38,628,350	4,434,984	43,063,334	34.8
Africa	Tanzania	57,917,100	18,496,130	9,655,451	28,151,581	48.6	22,644,170	7,809,555	30,453,725	52.6
*Total Africa*		*1,460,398,437*	*272,058,817*	*244,453,513*	*516,512,331*	*35.4*	*371,058,460*	1*99,269,506*	*570,327,96*6	*39.1*
America	Brazil	226,186,400	64,838,160	52,361,724	117,199,884	51.8	104,797,800	18,093,600	122,891,400	54.3
America	USA	366,044,900	24,799,830	14,636,370	39,436,200	10.8	23,617,820	4,533,182	28,151,002	7.7
America	Colombia	67,737,790	22,970,650	15,481,367	38,452,017	56.8	15,900,540	12,232,100	28,132,640	41.5
America	Mexico	152,410,700	13,652,610	10,830,574	24,483,184	16.1	28,110,530	13,082,960	41,193,490	27.0
*Total America*		*1,138,146,630*	*194,004,565*	*174,088,171*	*368,092,736*	*32.3*	*280,654,706*	*94,985,462*	*375,640,168*	*33.0*
Europe	Portugal	10,429,060	2,485,807	1,407,782	3,893,589	37.3	1,489,585	0	1,489,585	14.3
Europe	Italy	62,912,970	2,195,231	1,099,038	3,294,269	5.2	4,498,057	0	4,498,057	7.1
Europe	Spain	54,125,480	1,789,360	391,590	2,180,950	4.0	1,315,977	0	1,315,977	2.4
Europe	United Kingdom	67,997,440	1,747,281	0	1,747,281	2.6	1,341,649	0	1,341,649	2.0
*Total Europe*		*741,152,604*	*14,079,470*	*3,111,318*	17,190,788	*2.3*	*16,191,299*	*16,168*	*16,207,467*	*2.2*
Oceania	Australia	22,629,980	2,286,780	3,473,679	5,760,459	25.5	6,756,227	372,480	7,128,707	31.5
Oceania	Papua New Guinea	9,557,455	1,073,417	743,551	1,816,968	19.0	1,557,287	566,724	2,124,011	22.2
Oceania	New Zeeland	3,730,494	300,330	0	300,330	8.1	184,494	0	184,494	4.9
Oceania	Salomon Island	534,271	131,180	112,999	244,178	45.7	268,615	103,821	372,435	69.7
*Total Oceania*		*37,495,667*	*3,964,918*	*4,438,630*	*8,403,548*	*22.4*	*9,335,842*	*1,095,529*	*10,431,370*	*27.8*
*Total worldwide*		*8,443,323,187*	*1,339,920,835*	*1,339,991,724*	*2,679,912,559*	*31.7*	*2,104,440,338*	*1,344,334,868*	*3,448,775,207*	*40.8*

Only the four countries per continent that have the highest population at risk are shown. The complete list of countries by continent is given in Additional file 10: Table S6

*ST* *S. stercoralis*, *HKW* hookworms

## Discussion

In this study, we delimited the geographic areas in the world suitable for the development of *S. stercoralis* and hookworms. This was achieved using predictive modeling of the geographic distribution of species (MaxEnt), based on the environmental conditions (14 environmental variables) of the sites at which these species are present. In this way, we provide high-resolution information on a global scale across the world that includes over 9 million geographic points reporting the PP of *S. stercoralis* and hookworms, allowing risk areas with a surface area of up to 25 km^2^ to be identified. Based on this information, together with the population at every point, we estimate that in 2020 more than 2.6 billion people were at risk of being infected with *S. stercoralis* and more than 3.4 billion people were at risk of being infected with hookworms. This fine-grain analysis provides a more accurate and up-to-date estimate compared to previous reports for hookworms [35], and it is the first estimate of the populations at risk for *S. stercoralis* infections on a global scale. This information can be integrated with information on water and sanitation services, location of health centers, health indicators and even prevalence zones of other diseases, as it has been suggested that helminth infections may alter the response to other diseases, such as malaria or acquired immunodeficiency syndrome (AIDS) [36]. Such an integration of information would allow: (i) more efficient and integrated control strategies; (ii) the study of factors, such as drinking water, sewage, vaccination, level of education, among others, which could then be found with greater precision at the local level; (iii) possible antagonistic or synergistic effects with other diseases present in a given location; and (iv) possible locations at high risk for the emergence of drug resistance.

Currently, the main action undertaken for the control of STHs is the deworming of SAC [1, 2]. Chemotherapy is effective in the short term, but rapid reinfection after chemotherapy is common [36]. In addition, *S. stercoralis* and hookworms have a higher prevalence in the adult population [37–39]. Thus, mass deworming programs focused on SAC may be insufficient to eradicate the infection in high-risk areas for hookworms and *S. stercoralis*, where the optimal conditions for the hatching of hookworm eggs and the development *of S. stercoralis* larvae are found. In addition, *S. stercoralis* can generate free-living adult stages in soil, which, although they can only give rise to a single generation of infective larvae, contribute to amplification of the number of infective larvae in the environment [40]. Therefore, raster maps that precisely describe risk areas for *S. stercoralis* and hookworm infection are essential to identify hot spots that need to be reconsidered.

In this study we did not discard any geographic area to predict the PP with the models. Therefore, risk areas can be observed in highly developed countries, such as the southern USA and Japan. In these countries, economic development in the twentieth century has resulted in improved sanitary/hygienic conditions, access to clean water and the education of children and adults, which together with control programs has contributed to the elimination of helminth infections [41, 42]. However, given that the environmental conditions are still optimal for the development of STH infections, failed epidemiological surveillance can lead to a re-emergence of these diseases. Conversely, the main countries at risk in Asia, Africa and Latin America are at different stages in their implementation of PC programs and, consequently, with the progress achieved by these PC programs for *A. lumbricoides*, *T. trichiura* and hookworms [2]. Some countries that have > 90% of their population at risk for hookworm infection, such as Bangladesh, Belize, Cambodia, Dominican Republic, Ghana, Haiti, Laos, Myanmar, Nicaragua and Togo, have reached a coverage of > 75% for a period of > 5 years [2]. These countries present high-risk areas and have maintained chemotherapeutic treatment for > 5 years. However, if programs directed towards the improvement of water and sanitation facilities are not implemented to sustain the effects of these PC programs, these countries can be considred to be targets for evaluation of the effectiveness of these programs by enhanced post-intervention surveys and monitoring of drug resistance.

It has been shown that the ranges of temperature and precipitation are the main conditioning factors for STH [5, 35, 43]. Our results show that although the annual precipitation ranges are the same for *S. stercoralis* and hookworms, the annual temperature presented a more restricted range for hookworms (26–30 ºC). These results are in agreement with those of studies demonstrating that the optimum temperature for the development of hookworm larvae is 20–30 ºC, while temperatures exceeding 35–40 ºC cause the death of the eggs [44, 45]. In addition, our study shows that hookworms had a closer association with rural areas, which agrees with the highest prevalence reported in these areas compared to urban areas [35]. An interesting finding was the high suitability of mangroves for hookworms. This is a worrying observation given that mangroves in Asia are known to be used to dispose of fecal matter and that a high frequency of STH eggs, including hookworms, have already been reported in mangroves [46]. In addition, the optimal pH range range (5.5–6.5) for both species corresponds to a moderately acidic soil, which is optimal for the hatching of hookworm eggs [45]. Also, moderately acidic soil ensures adequate availability of nutrients for plants and are expected in rural areas [47].

Previous studies have reported the relationship between the risk for *S. stercoralis* and hookworm infection and soil OC content [43, 48, 49], but the relationship between the development of these parasites and soil OC content remains poorly understood. In studies carried out in Cambodia, higher prevalences of *S. stercoralis* were observed in areas with low soil OC content [48]; however, this could be due the analyzed areas being rural areas where deforestation produced a decrease in the organic content of the soil. For hookworms, higher prevalences were observed in areas of Indonesia with higher soil OC content [49]. Our results suggest that hookworms are more sensitive to high values of soil OC, since as OC content increases to levels > 25 g/kg, the PP increases. *Strongyloides stercoralis* is sensitive to values of soil OC < 50 g/kg. Differences in sensitivity to soil OC content between *S. stercoralis* and hookworms may possibly be due to differences in their life-cycles: in *S. stercoralis*, rhabditiform larvae are released into the soil, where they feed in the environment and then molt into filariform larvae or free-living adults [40]; for hookworms, eggs are released into the environment and hatch into rhabditiform larvae [50].

We have reported previously that there is an association between the prevalence of *S. stercoralis* and hookworms [3]. In the present study, we observed that although they share 68% of their ecological niches, their niches are different (*p* < 0.01). In addition, the relationship between PP and prevalence is very different between the two species. For *S. stercoralis*, definite limits of PP correspond to different prevalences (Fig. 3a), which suggest that environmental characteristics exert a strong pressure on the development and spread of *S. stercoralis* infection. Hookworms, however, were found to be very different, and in the present study, a difference in PP was only present when prevalence was zero. This result indicates that when an approximate PP of 0.30 (Fig. 3b) is exceeded, hookworms can develop in that location, but the prevalence cannot be predicted by environmental conditions at the site. Similar results were found in a study conducted in different schools in Timor Leste, where three different statistical approaches (mixed logistic regression, recursive partitioning and Bayesian networks) were used to evaluate the association between hookworms and environmental variables, socioeconomic variables and washing habits [51]. In that study, environmental variables were the least relevant predictors of hookworm outcomes [51]. Therefore, we conclude that hookworm prevalence is influenced by variables other than environmental factors. Socioeconomic variables, such as poverty, overcrowding, type of floor in dwelling and household sanitation facilities have been reported to have a positive association with the prevalence of hookworms [52, 53]. However, these same variables are risk factors for *S. stercoralis*; therefore, in addition to differences in exposure to infection (due to household, socioeconomic, climatic, environmental and occupational factors), the prevalence of hookworms may be highly influenced by differences in host susceptibility to infection and the ability of the host to mount an effective immune response (due to genetic and/or nutritional factors) [54]. Hookworm prevalence may be largely influenced by: (i) the rate of exposure to infectious stages; (ii) the rate of successful establishment overcoming host immunity; and (iii) the mortality rate of the adult parasites, and how these processes vary with the age of the host [55, 56].

A limitation of this study is the relatively low number of presence points, due to the fact that only a small percentage of epidemiological studies accurately report the geographic location of the evaluated areas. In addition, the need to use pre-intervention data produced an even greater reduction in data. However, since > 4500 models were rigorously evaluated for each species, it was possible to obtain models with high statistical significance that are consistent with the biological behavior of these species. Another limitation of the present study is that data from epidemiological surveys were used; these data orginate from studies of different designs and the studies are biased to epidemiological areas with a history of STH infections. This results the environmental variables being measured in a limited number of points of presence of the studied species. An additional limitation is that the two species of hookworms were modeled as if they were a single species. *Necator americanus* is the most abundant and most widely distributed hookworm worldwide, while *Ancylostoma duodenale* is focally endemic. In addition, *A. duodenale* is more resistant to environmental changes due to its ability to undergo arrested development in host tissues during periods of dryness or cold [54]. This raises the possibility that *N. americanus* and *A. duodenale* have different ecological niches.

## Conclusion

In this study we created and make available raster maps that indicate those areas throughout the world that are suitable for the presence of *S. stercoralis* and hookworm infections at a scale of 25 km^2^, along with the human population at each point and the country to which it belongs. Our aim was to estimate the population at risk for infection by each of these STH species, at the level of a locality or village. The integration of the information provided by this study with data on other variables (e.g. WASH, other disease prevalences, interventions) could result in more efficient survey and control strategies.

## Supplementary Information

**Additional file 1: Table S1**: Surveys used for the ecological niche model of *S. stercoralis.*

**Additional file 2: Table S2:** Surveys used for the ecological niche model of hookworms.

**Additional file 3: Table S3:** Nested anova of mobility area (M) of *S. stercoralis* and hookworms.

**Additional file 4: Total_data:** csv file with the longitude, latitude, PP (for each species), country identification and population number for each pixel of the hookworm and S. stercoralis ecological niche models.

**Additional file 5: ** **Table S4:** Final niche models for *S. stercoralis*.

**Additional file 6: Table S5: **Final niche models for hookworms.

**Additional file 7: **asc file* S. stercoralis*_Niche_Raster_map. 

**Additional file 8: **asc file HKW_Niche_Raster_Map. 

**Additional file 9: Figure S1:** Scatter plot graphs of the prevalence versus the probability of presence. 

**Additional file 10: Table S6:** Risk population of *S. stercoralis* and hookworms. 

## Data Availability

All data used in this study are freely accessible, and can be found in the manuscript and in supplementary material.
