# Supplementary material for: Mapping the global distribution of Strongyloides stercoralis and hookworms by ecological niche modeling
Source: Parasit Vectors. 2022 Jun 8;15:197. doi: 10.1186/s13071-022-05284-w (PMC9178904; doi:10.1186/s13071-022-05284-w)
Supplement: Supplementary file 1 — Additional file 1: Table S1: Surveys used for the ecological niche model of S. stercoralis. [file 13071_2022_5284_MOESM1_ESM.docx]

# Additional file 1: Table S1: Surveys used for the ecological niche model of S*. stercoralis*

| **Author** | **Year** | **Title** | **Journal** | **Longitud** | **Latitud** | **Prevalence** | **use of data** |
| --- | --- | --- | --- | --- | --- | --- | --- |
| 5_Al-Hindi_2002 | 2002 | Prevalence of intestinal parasites among school children in Deir El-Balah Town in Gaza Strip, Palestine | Annals of Saudi Medicine | 34.327 | 31.3706 | 1.7 | Final validation of the model |
| Abah, A. E. Arene, F. O. | 2015 | Status of Intestinal Parasitic Infections among Primary School Children in Rivers State, Nigeria | Journal of Parasitology Research | 6.9639 | 4.5699 | 7.14 | Design and calibration of the model |
| Abah, A. E. Arene, F. O. | 2015 | Status of Intestinal Parasitic Infections among Primary School Children in Rivers State, Nigeria | Journal of Parasitology Research | 7.1252 | 4.8869 | 7.14 | Design and calibration of the model |
| Abah, A. E. Arene, F. O. | 2015 | Status of Intestinal Parasitic Infections among Primary School Children in Rivers State, Nigeria | Journal of Parasitology Research | 6.6585 | 5.0828 | 7.14 | Design and calibration of the model |
| Agbolade, O.M., Agu, N.C., Adesanya, O.O., Odejayi, A.O., Adigun, A.A., Adesanlu, E.B., Ogunleye, F.G., Sodimu, A.O., Adeshina, S.A., Bisiriyu, G.O., Omotoso, O.I., Udia, K.M | 2007 | Intestinal Helminthiases and Schistosomiasis among School Children in an Urban Center and Some Rural Communities in Southwest Nigeria | The Korean Journal of Parasitology | 3.8101 | 6.8582 | 2 | Final validation of the model |
| Agbolade, Olufemi Moses, Ndubuisi Chinweike Agu, Oluseyi Olusegun Adesanya, Adedayo Olugbenga Odejayi, Aliu Adekunle Adigun, Emmanuel Babatunde Adesanlu, Flourish George Ogunleye, et al. | 2007 | Intestinal Helminthiases and Schistosomiasis among School Children in an Urban Center and Some Rural Communities in Southwest Nigeria | The Korean Journal of Parasitology | 3.7691 | 6.9104 | 5 | Final validation of the model |
| Aguiar, J. I. A. Goncalves, A. Q. Sodre, F. C. Pereira, S. D. R. Boia, M. N. De Lemos, E. R. S. Daher, R. R. | 2007 | Intestinal protozoa and helminths among Terena Indians in the State of Mato Grosso do Sul: High prevalence of Blastocystis hominis | Revista da Sociedade Brasileira de Medicina Tropical | -54.9723 | -20.9262 | 3.8 | Design and calibration of the model |
| Ahmad, A.F., Hadip, F., Ngui, R., Lim, Y.A.L., Mahmud, R. | 2013 | Serological and molecular detection of Strongyloides stercoralis infection among an Orang Asli community in Malaysia | Parasitology Research | 101.88 | 3.2199 | 5.6 | Final validation of the model |
| Aimpun, P. Hshieh, P. | 2004 | Survey for intestinal parasites in Belize, Central America | Southeast Asian Journal of Tropical Medicine & Public Health | -88.7986 | 16.3474 | 1 | Design and calibration of the model |
| Aimpun, P. Hshieh, P. | 2004 | Survey for intestinal parasites in Belize, Central America | Southeast Asian Journal of Tropical Medicine & Public Health | -88.741 | 16.4411 | 1 | Design and calibration of the model |
| Aimpun, P. Hshieh, P. | 2004 | Survey for intestinal parasites in Belize, Central America | Southeast Asian Journal of Tropical Medicine & Public Health | -88.9066 | 16.2155 | 1 | Design and calibration of the model |
| Aimpun, P. Hshieh, P. | 2004 | Survey for intestinal parasites in Belize, Central America | Southeast Asian Journal of Tropical Medicine & Public Health | -88.6255 | 16.4725 | 1 | Design and calibration of the model |
| Akogun, O.B | 1989 | Some Social Aspects of Helminthiasis Among the People of Gumau District, Bauchi State, Nigeria | Journal of Tropical Medicine and Hygiene | 9.5 | 10.4995 | 0.4 | Final validation of the model |
| Alsubaie, A. S. R. Azazy, A. A. Omer, E. O. Al-Shibani, L. A. Al-Mekhlafi, A. Q. Al-Khawlani, F. A. | 2016 | Pattern of parasitic infections as public health problem among school children: A comparative study between rural and urban areas | Journal of Taibah University Medical Sciences | 44.1651 | 13.9693 | 0.8 | Design and calibration of the model |
| Amor, A. Rodriguez, E. Saugar, J. M. Arroyo, A. Lopez-Quintana, B. Abera, B. Yimer, M. Yizengaw, E. Zewdie, D. Ayehubizu, Z. Hailu, T. Mulu, W. Echazu, A. Krolewieki, A. J. Aparicio, P. Herrador, Z. Anegagrie, M. Benito, A | 2016 | High prevalence of Strongyloides stercoralis in school-aged children in a rural highland of north-western Ethiopia: the role of intensive diagnostic work-up | Parasites & Vectors | 37.2087 | 11.3705 | 20.7 | Design and calibration of the model |
| Amor, A. Rodriguez, E. Saugar, J. M. Arroyo, A. Lopez-Quintana, B. Abera, B. Yimer, M. Yizengaw, E. Zewdie, D. Ayehubizu, Z. Hailu, T. Mulu, W. Echazu, A. Krolewieki, A. J. Aparicio, P. Herrador, Z. Anegagrie, M. Benito, A | 2016 | High prevalence of Strongyloides stercoralis in school-aged children in a rural highland of north-western Ethiopia: the role of intensive diagnostic work-up | Parasites & Vectors | 37.2831 | 11.4731 | 20.7 | Design and calibration of the model |
| Amor, A. Rodriguez, E. Saugar, J. M. Arroyo, A. Lopez-Quintana, B. Abera, B. Yimer, M. Yizengaw, E. Zewdie, D. Ayehubizu, Z. Hailu, T. Mulu, W. Echazu, A. Krolewieki, A. J. Aparicio, P. Herrador, Z. Anegagrie, M. Benito, A | 2016 | High prevalence of Strongyloides stercoralis in school-aged children in a rural highland of north-western Ethiopia: the role of intensive diagnostic work-up | Parasites & Vectors | 37.3546 | 11.707 | 20.7 | Design and calibration of the model |
| Amor, A. Rodriguez, E. Saugar, J. M. Arroyo, A. Lopez-Quintana, B. Abera, B. Yimer, M. Yizengaw, E. Zewdie, D. Ayehubizu, Z. Hailu, T. Mulu, W. Echazu, A. Krolewieki, A. J. Aparicio, P. Herrador, Z. Anegagrie, M. Benito, A | 2016 | High prevalence of Strongyloides stercoralis in school-aged children in a rural highland of north-western Ethiopia: the role of intensive diagnostic work-up | Parasites & Vectors | 37.5834 | 11.4857 | 20.7 | Design and calibration of the model |
| Appleton, C.C., Maurihungirire, M., Gouws, E. | 1999 | The distribution of helminth infections along the coastal plain of Kwazulu-Natal province, South Africa | Annals of Tropical Medicine and Parasitology | 30.3147 | -30.8889 | 0.6 | Final validation of the model |
| Appleton, C.C., Maurihungirire, M., Gouws, E. | 1999 | The distribution of helminth infections along the coastal plain of Kwazulu-Natal province, South Africa | Annals of Tropical Medicine and Parasitology | 32.3447 | -26.9464 | 1.3 | Final validation of the model |
| Appleton, C.C., Maurihungirire, M., Gouws, E. | 1999 | The distribution of helminth infections along the coastal plain of Kwazulu-Natal province, South Africa | Annals of Tropical Medicine and Parasitology | 30.5856 | -30.5403 | 1.8 | Final validation of the model |
| Appleton, C.C., Maurihungirire, M., Gouws, E. | 1999 | The distribution of helminth infections along the coastal plain of Kwazulu-Natal province, South Africa | Annals of Tropical Medicine and Parasitology | 30.7289 | -30.2361 | 1.9 | Final validation of the model |
| Appleton, C.C., Maurihungirire, M., Gouws, E. | 1999 | The distribution of helminth infections along the coastal plain of Kwazulu-Natal province, South Africa | Annals of Tropical Medicine and Parasitology | 31.4972 | -29.1917 | 2.4 | Final validation of the model |
| Appleton, C.C., Maurihungirire, M., Gouws, E. | 1999 | The distribution of helminth infections along the coastal plain of Kwazulu-Natal province, South Africa | Annals of Tropical Medicine and Parasitology | 31.2278 | -29.4861 | 3 | Final validation of the model |
| Appleton, C.C., Maurihungirire, M., Gouws, E. | 1999 | The distribution of helminth infections along the coastal plain of Kwazulu-Natal province, South Africa | Annals of Tropical Medicine and Parasitology | 32.3447 | -28.1119 | 5.4 | Final validation of the model |
| Appleton, C.C., Maurihungirire, M., Gouws, E. | 1999 | The distribution of helminth infections along the coastal plain of Kwazulu-Natal province, South Africa | Annals of Tropical Medicine and Parasitology | 32.2911 | -27.4522 | 5.4 | Final validation of the model |
| Appleton, C.C., Maurihungirire, M., Gouws, E. | 1999 | The distribution of helminth infections along the coastal plain of Kwazulu-Natal province, South Africa | Annals of Tropical Medicine and Parasitology | 32.5461 | -27.3306 | 6.9 | Final validation of the model |
| Appleton, C.C., Maurihungirire, M., Gouws, E. | 1999 | The distribution of helminth infections along the coastal plain of Kwazulu-Natal province, South Africa | Annals of Tropical Medicine and Parasitology | 31.8975 | -28.8589 | 18 | Final validation of the model |
| Araujo, Claudio Fernández Fernández, Claudia Leite | 2005 | Prevalência de parasitoses intestinais na cidade de Eirunepé, Amazonas | Revista da Sociedade Brasileira de Medicina Tropical | -69.8681 | -6.6596 | 1 | Design and calibration of the model |
| Assis, E. M. de Olivieria, R. C. de Moreira, L. E. Pena, J. L. Rodrigues, L. C. Machado-Coelho, G. L. L. | 2013 | Prevalence of intestinal parasites in the Maxakali indigenous community in Minas Gerais, Brazil, 2009 | Cadernos de Saúde Pública | -40.5934 | -16.8625 | 5.9 | Design and calibration of the model |
| Auer, C. | 1990 | Health status of children living in a squatter area of Manila, Philippines, with particular emphasis on intestinal parasitoses | The Southeast Asian Journal of Tropical Medicine and Public Health | 120.9643 | 14.632 | 0.8 | Final validation of the model |
| Babatunde, S. K. Adedayo, M. R. Ajiboye, A. E. Sunday, O. Ameen, N. | 2013 | Soil-transmitted helminth infections among school children in rural communities of Moro Local Government Area, Kwara State, Nigeria | African Journal of Microbiology Research | 4.4723 | 8.7082 | 7.1 | Design and calibration of the model |
| Bangs, M.J., Andersen, E.M., Anthony, R.L. | 1996 | Intestinal parasites of humans in a highland community of Irian Jaya, Indonesia | Annals of Tropical Medicine and Parasitology | 140.6325 | -4.9095 | 0.5 | Final validation of the model |
| Barbosa, C. V. Barreto, M. M. Andrade, R. J. Sodre, F. d'Avila-Levy, C. M. Peralta, J. M. Igreja, R. P. de Macedo, H. W. Santos, H. L. C. | 2018 | Intestinal parasite infections in a rural community of Rio de Janeiro (Brazil): Prevalence and genetic diversity of Blastocystis subtypes | PLoS ONE | -42.6892 | -22.0461 | 0.7 | Design and calibration of the model |
| Bayeh, Abera Genetu, Alem Mulat, Yimer Herrador, Z. | 2013 | Epidemiology of soil-transmitted helminths, schistosoma mansoni, and haematocrit values among schoolchildren in Ethiopia | Journal of Infection in Developing Countries | 37.3795 | 11.5726 | 3.47 | Design and calibration of the model |
| Becker, S. L. Sieto, B. Silue, K. D. Adjossan, L. Kone, S. Hatz, C. Kern, W. V. N'Goran, E. K. Utzinger, J. | 2011 | Diagnosis, clinical features, and self-reported morbidity of Strongyloides stercoralis and hookworm infection in a Co-endemic setting | PLoS Neglected Tropical Diseases | -5.217 | 6.2328 | 12.7 | Design and calibration of the model |
| Beltramino, D., Lurá, M.C., Carrera, E. | 2003 | El tratamiento antihelmíntico selectivo frente al tratamiento masivo. Experiencia en dos comunidades hiperendémicas. | Revista panamericana de salud publica | -60.7336 | -31.5961 | 2.6 | Final validation of the model |
| Bencke, A., Artuso, G.L., Souza, R., Lima, N | 2006 | Enteroparasitosesem escolares residentes na periferia | Revista De Patologia Tropical | -51.1049 | -30.0427 | 9.8 | Final validation of the model |
| Boko, P. M. Ibikounle, M. Onzo-Aboki, A. Tougoue, J. J. Sissinto, Y. Batcho, W. Kinde-Gazard, D. Kabore, A. | 2016 | Schistosomiasis and Soil Transmitted Helminths Distribution in Benin: A Baseline Prevalence Survey in 30 Districts | PLoS ONE | 1.115 | 10.2231 | 0.05 | Design and calibration of the model |
| Boko, P. M. Ibikounle, M. Onzo-Aboki, A. Tougoue, J. J. Sissinto, Y. Batcho, W. Kinde-Gazard, D. Kabore, A. | 2016 | Schistosomiasis and Soil Transmitted Helminths Distribution in Benin: A Baseline Prevalence Survey in 30 Districts | PLoS ONE | 1.1328 | 10.1264 | 0.05 | Design and calibration of the model |
| Boko, P. M. Ibikounle, M. Onzo-Aboki, A. Tougoue, J. J. Sissinto, Y. Batcho, W. Kinde-Gazard, D. Kabore, A. | 2016 | Schistosomiasis and Soil Transmitted Helminths Distribution in Benin: A Baseline Prevalence Survey in 30 Districts | PLoS ONE | 1.5536 | 10.0544 | 0.05 | Design and calibration of the model |
| Boko, P. M. Ibikounle, M. Onzo-Aboki, A. Tougoue, J. J. Sissinto, Y. Batcho, W. Kinde-Gazard, D. Kabore, A. | 2016 | Schistosomiasis and Soil Transmitted Helminths Distribution in Benin: A Baseline Prevalence Survey in 30 Districts | PLoS ONE | 1.6156 | 10.1997 | 0.05 | Design and calibration of the model |
| Boko, P. M. Ibikounle, M. Onzo-Aboki, A. Tougoue, J. J. Sissinto, Y. Batcho, W. Kinde-Gazard, D. Kabore, A. | 2016 | Schistosomiasis and Soil Transmitted Helminths Distribution in Benin: A Baseline Prevalence Survey in 30 Districts | PLoS ONE | 1.6453 | 8.0161 | 0.05 | Design and calibration of the model |
| Boko, P. M. Ibikounle, M. Onzo-Aboki, A. Tougoue, J. J. Sissinto, Y. Batcho, W. Kinde-Gazard, D. Kabore, A. | 2016 | Schistosomiasis and Soil Transmitted Helminths Distribution in Benin: A Baseline Prevalence Survey in 30 Districts | PLoS ONE | 1.6586 | 8.1589 | 0.05 | Design and calibration of the model |
| Boko, P. M. Ibikounle, M. Onzo-Aboki, A. Tougoue, J. J. Sissinto, Y. Batcho, W. Kinde-Gazard, D. Kabore, A. | 2016 | Schistosomiasis and Soil Transmitted Helminths Distribution in Benin: A Baseline Prevalence Survey in 30 Districts | PLoS ONE | 1.7281 | 10.5639 | 0.05 | Design and calibration of the model |
| Boko, P. M. Ibikounle, M. Onzo-Aboki, A. Tougoue, J. J. Sissinto, Y. Batcho, W. Kinde-Gazard, D. Kabore, A. | 2016 | Schistosomiasis and Soil Transmitted Helminths Distribution in Benin: A Baseline Prevalence Survey in 30 Districts | PLoS ONE | 1.7558 | 10.3811 | 0.05 | Design and calibration of the model |
| Boko, P. M. Ibikounle, M. Onzo-Aboki, A. Tougoue, J. J. Sissinto, Y. Batcho, W. Kinde-Gazard, D. Kabore, A. | 2016 | Schistosomiasis and Soil Transmitted Helminths Distribution in Benin: A Baseline Prevalence Survey in 30 Districts | PLoS ONE | 1.8806 | 7.9825 | 0.05 | Design and calibration of the model |
| Boko, P. M. Ibikounle, M. Onzo-Aboki, A. Tougoue, J. J. Sissinto, Y. Batcho, W. Kinde-Gazard, D. Kabore, A. | 2016 | Schistosomiasis and Soil Transmitted Helminths Distribution in Benin: A Baseline Prevalence Survey in 30 Districts | PLoS ONE | 1.8856 | 8.4175 | 0.05 | Design and calibration of the model |
| Boko, P. M. Ibikounle, M. Onzo-Aboki, A. Tougoue, J. J. Sissinto, Y. Batcho, W. Kinde-Gazard, D. Kabore, A. | 2016 | Schistosomiasis and Soil Transmitted Helminths Distribution in Benin: A Baseline Prevalence Survey in 30 Districts | PLoS ONE | 1.9222 | 6.4033 | 0.05 | Design and calibration of the model |
| Boko, P. M. Ibikounle, M. Onzo-Aboki, A. Tougoue, J. J. Sissinto, Y. Batcho, W. Kinde-Gazard, D. Kabore, A. | 2016 | Schistosomiasis and Soil Transmitted Helminths Distribution in Benin: A Baseline Prevalence Survey in 30 Districts | PLoS ONE | 2.0161 | 7.7733 | 0.05 | Design and calibration of the model |
| Boko, P. M. Ibikounle, M. Onzo-Aboki, A. Tougoue, J. J. Sissinto, Y. Batcho, W. Kinde-Gazard, D. Kabore, A. | 2016 | Schistosomiasis and Soil Transmitted Helminths Distribution in Benin: A Baseline Prevalence Survey in 30 Districts | PLoS ONE | 2.0822 | 7.8961 | 0.05 | Design and calibration of the model |
| Boonjaraspinyo, S. Boonmars, T. Kaewsamut, B. Ekobol, N. Laummaunwai, P. Aukkanimart, R. Wonkchalee, N. Juasook, A. Sriraj, P. | 2013 | A cross-sectional study on intestinal parasitic infections in rural communities, northeast Thailand | Korean Journal of Parasitology | 102.8236 | 16.4322 | 0.4 | Design and calibration of the model |
| Borda, C.E., Felega, J., Rosa, J.R., Maidana, C | 1996 | Intestinal parasitism in San Cayetano, Corrientes, Argentina | Bulletin of the Pan American Health Organization | -58.6939 | -27.569 | 2 | Final validation of the model |
| Bracho M, Angela Rivero-Rodríguez, Zulbey Rios P, Melary Atencio T, Ricardo Villalobos P, Rafael Rodríguez, Luis | 2014 | Parasitosis intestinales en niños y adolescentes de la etnia Yukpa de Toromo, estado Zulia, Venezuela: Comparación de los años 2002 Y 2012 | Kasmera | -72.7138 | 10.0488 | 14.5 | Design and calibration of the model |
| Brandelli, C. L. C. Carli, G. A. de Macedo, A. J. Tasca, T. | 2012 | Intestinal parasitism and socio-environmental factors among Mbyá-Guarani indians, Porto Alegre, Rio Grande do Sul, Brazil | Revista do Instituto de Medicina Tropical de São PauloRevista do Instituto de Medicina Tropical de São Paulo | -51.127 | -30.0906 | 3.2 | Design and calibration of the model |
| Cabada, M.M., Lopez, M., Arque, E., White, A.C | 2014 | Prevalence of soil-transmitted helminths after mass albendazole administration in an indigenous community of the Manu jungle in Peru | Pathogens and global health | -71.87 | -11.76 | 5.6 | Final validation of the model |
| Cardozo Ocampos, G. E. Cañete Duarte, Z. Lenartovicz, V. | 2015 | Frecuencia de enteroparásitos en niños y niñas del primer ciclo de la educación escolar básica de escuelas públicas de ciudad del este, Paraguay | Memorias del Instituto de Investigaciones en Ciencias de la Salud | -54.6162 | -25.5162 | 2 | Design and calibration of the model |
| Carme, B. Motard, A. Bau, P. Day, C. Aznar, C. Moreau, B. | 2002 | Intestinal parasitoses among Wayampi Indians from French Guiana | Parasite | -52.9017 | 2.1969 | 16.3 | Design and calibration of the model |
| Carpio, I. | 2007 | Presencia de Strongyloides stercoralis en un estudio sobre enteroparasitosis en escolares del asentamiento humano "La Candelaria", distrito de Chancay, provincia de Huaral, departamento de Lima | Acta Medica Peruana | -77.2936 | -11.4977 | 1.1 | Final validation of the model |
| Carvalho, Gabriela Lanna Xavier de Moreira, Luciano Evangelista Pena, João Luiz Marinho, Carolina Coimbra Bahia, Maria Terezinha Machado-Coelho, George Luiz Lins | 2012 | A comparative study of the TF-Test®, Kato-Katz, Hoffman-Pons-Janer, Willis and Baermann-Moraes coprologic methods for the detection of human parasitosis | Memórias do Instituto Oswaldo Cruz | -41.2654 | -18.0632 | 9.1 | Design and calibration of the model |
| Cerdas, Carlos Araya, Edna Coto, Susana | 2003 | Parásitos intestinales en la Escuela 15 de agosto, Tirrases de Curridabat, Costa Rica. Mayo-Junio de 2002 | Revista Costarricense de Ciencias Médicas | -84.0247 | 9.9014 | 0.31 | Design and calibration of the model |
| Chacin-Bonilla, L., Bonilla, E., Parra, A.M., Estevez, J., Morales, L.M., Suarez, H. | 1992 | Prevalence of Entamoeba histolytica and other intestinal parasites in a community from Maracaibo | Annals of Tropical Medicine and Parasitology | -71.6471 | 10.5036 | 8.4 | Final validation of the model |
| Chessed, G. Kwalagbe, B. Furo, N. A. | 2005 | Intestinal helminthiases among school children in Gyawana District, Adamawa State, Nigeria | Global Journal of Pure and Applied Sciences | 11.9215 | 9.5894 | 6.6 | Design and calibration of the model |
| Chukwuma, M. C. Ekejindu, I. M. Agbakoba, N. R. Ezeagwuna, D. A. Anaghalu, I. C. Nwosu, D. C. | 2009 | The prevalence and risk factors of geohelminth infections among primary school children in Ebenebe Town, Anambra State, Nigeria | Middle East Journal of Scientific Research | 7.133 | 6.337 | 5.9 | Design and calibration of the model |
| Conlan, J. V. Khamlome, B. Vongxay, K. Elliot, A. Pallant, L. Sripa, B. Blacksell, S. D. Fenwick, S. Thompson, R. C | 2012 | Soil-transmitted helminthiasis in Laos: a community-wide cross-sectional study of humans and dogs in a mass drug administration environment | American Journal of Tropical Medicine & Hygiene | 101.9856 | 20.6902 | 8.9 | Design and calibration of the model |
| Conlan, J. V. Khamlome, B. Vongxay, K. Elliot, A. Pallant, L. Sripa, B. Blacksell, S. D. Fenwick, S. Thompson, R. C | 2012 | Soil-transmitted helminthiasis in Laos: a community-wide cross-sectional study of humans and dogs in a mass drug administration environment | American Journal of Tropical Medicine & Hygiene | 102.1832 | 19.7589 | 8.9 | Design and calibration of the model |
| Conlan, J. V. Khamlome, B. Vongxay, K. Elliot, A. Pallant, L. Sripa, B. Blacksell, S. D. Fenwick, S. Thompson, R. C | 2012 | Soil-transmitted helminthiasis in Laos: a community-wide cross-sectional study of humans and dogs in a mass drug administration environment | American Journal of Tropical Medicine & Hygiene | 104.2274 | 20.4096 | 8.9 | Design and calibration of the model |
| Cooper, P. J. Chico, M. E. Gaus, D. Griffin, G. E. | 2003 | Relationship between bacille Calmette-Guerin vaccination, Mantoux test positivity, and geohelminth infection | Transactions of the Royal Society of Tropical Medicine & Hygiene | -78.4398 | -0.2219 | 3 | Design and calibration of the model |
| Cooper, P. J. Chico, M. E. Gaus, D. Griffin, G. E. | 2003 | Relationship between bacille Calmette-Guerin vaccination, Mantoux test positivity, and geohelminth infection | Transactions of the Royal Society of Tropical Medicine & Hygiene | -78.8922 | 0.0246 | 3 | Design and calibration of the model |
| Couto, L. D. Tibirica, S. H. C. Pinheiro, I. O. Mitterofhe, A. Lima, A. C. Castro, M. F. Goncalves, M. Silva, M. R. Guimaraes, R. J. P. S. Rosa, F. M. Coimbra, E. S. | 2014 | Neglected tropical diseases: Prevalence and risk factors for schistosomiasis and soil-transmitted helminthiasis in a region of Minas Gerais State, Brazil | Transactions of the Royal Society of Tropical Medicine and Hygiene | -43.0155 | -21.5365 | 1.2 | Design and calibration of the model |
| Dankwa, K. Addy-Lamptey, P. Latif, A. Essien-Baidoo, S. Ephraim, R. K. D. Gavor-Kwashi, C. E. K. Nuvor, S. V. | 2017 | Intestinal parasitic infections among primary school pupils in Elmina, a fishing community in Ghana | International Journal of Medical and Health Sciences | -1.2706 | 5.1241 | 1 | Design and calibration of the model |
| de Alegria, Mlar Colmenares, K. Espasa, M. Amor, A. Lopez, I. Nindia, A. Kanjala, J. Guilherme, D. Sulleiro, E. Barriga, B. Gil, E. Salvador, F. Bocanegra, C. Lopez, T. Moreno, M. Molina, I. | 2017 | Prevalence of Strongyloides stercoralis and Other Intestinal Parasite Infections in School Children in a Rural Area of Angola: A Cross-Sectional Study | American Journal of Tropical Medicine & Hygiene | 14.2419 | -13.0403 | 12.2 | Design and calibration of the model |
| Devi, U. Borkakoty, B. Mahanta, J. | 2011 | Strongyloidiasis in Assam, India: A community-based study | Tropical Parasitology | 94.9113 | 27.4729 | 8.5 | Design and calibration of the model |
| Easton, A. V. Oliveira, R. G. O'Connell, E. M. Kepha, S. Mwandawiro, C. S. Njenga, S. M. Kihara, J. H. Mwatele, C. Odiere, M. R. Brooker, S. J. Webster, J. P. Anderson, R. M. Nutman, T. B | 2016 | Multi-parallel qPCR provides increased sensitivity and diagnostic breadth for gastrointestinal parasites of humans: field-based inferences on the impact of mass deworming | Parasites & Vectors | 34.5941 | 0.5097 | 1 | Design and calibration of the model |
| Erlanger, T. E. Sayasone, S. Krieger, G. R. Kaul, S. Sananikhom, P. Tanner, M. Odermatt, P. Utzinger, J. | 2008 | Baseline health situation of communities affected by the Nam Theun 2 hydroelectric project in central Lao PDR and indicators for monitoring | International Journal of Environmental Health Research | 105.1881 | 17.6975 | 1.4 | Design and calibration of the model |
| Faulkner, C. T. Garcia, B. B. Logan, M. H. New, J. C. Patton, S. | 2003 | Prevalence of endoparasitic infection in children and its relation with cholera prevention efforts in Mexico | Pan American Journal of Public Health | -97.8098 | 25.6673 | 0.22 | Design and calibration of the model |
| Fontanet, B. | 2000 | Epidemiology of infections with intestinal parasites and human immunodeficiency virus (HIV) among sugar-estate residents in Ethiopia | Annals of Tropical Medicine and Parasitology | 39.2271 | 8.4517 | 13 | Final validation of the model |
| Fontes, G. Lessa Oliveira, K. K. Lessa Oliveira, A. K. Mauricio da Rocha, E. M. | 2003 | Influence of specific treatment of intestinal parasites and schistosomiasis on prevalence in students in Barra de Santo Antonio, AL. [Portuguese] | Revista da Sociedade Brasileira de Medicina Tropical | -35.508 | -9.4038 | 51.3 | Design and calibration of the model |
| Forrer, A. Khieu, V. Schar, F. Vounatsou, P. Chammartin, F. Marti, H. Muth, S. Odermatt, P. | 2018 | Strongyloides stercoralis and hookworm co-infection: spatial distribution and determinants in Preah Vihear Province, Cambodia | Parasites & Vectors | 104.6988 | 13.819 | 48.6 | Design and calibration of the model |
| Forrer, A. Khieu, V. Schar, F. Vounatsou, P. Chammartin, F. Marti, H. Muth, S. Odermatt, P. | 2018 | Strongyloides stercoralis and hookworm co-infection: spatial distribution and determinants in Preah Vihear Province, Cambodia | Parasites & Vectors | 104.9012 | 13.5786 | 48.6 | Design and calibration of the model |
| Forrer, A. Khieu, V. Schar, F. Vounatsou, P. Chammartin, F. Marti, H. Muth, S. Odermatt, P. | 2018 | Strongyloides stercoralis and hookworm co-infection: spatial distribution and determinants in Preah Vihear Province, Cambodia | Parasites & Vectors | 104.941 | 14.2174 | 48.6 | Design and calibration of the model |
| Forrer, A. Khieu, V. Schar, F. Vounatsou, P. Chammartin, F. Marti, H. Muth, S. Odermatt, P. | 2018 | Strongyloides stercoralis and hookworm co-infection: spatial distribution and determinants in Preah Vihear Province, Cambodia | Parasites & Vectors | 105.0356 | 13.8556 | 48.6 | Design and calibration of the model |
| Forrer, A. Khieu, V. Schar, F. Vounatsou, P. Chammartin, F. Marti, H. Muth, S. Odermatt, P. | 2018 | Strongyloides stercoralis and hookworm co-infection: spatial distribution and determinants in Preah Vihear Province, Cambodia | Parasites & Vectors | 105.2576 | 13.6537 | 48.6 | Design and calibration of the model |
| Gamboa, María Inés Kozubsky, Leonora Eugenia Costas, María Elena Garraza, Mariela Cardozo, Marta Inés Susevich, María Laura Magistrello, Paula Natalia Navone, Graciela Teresa | 2009 | Asociación entre geohelmintos y condiciones socioambientales en diferentes poblaciones humanas de Argentina | Revista Panamericana Salud Publica | -54.893 | -27.0964 | 0 | Figure 3a |
| Gamboa, María Inés Kozubsky, Leonora Eugenia Costas, María Elena Garraza, Mariela Cardozo, Marta Inés Susevich, María Laura Magistrello, Paula Natalia Navone, Graciela Teresa | 2009 | Asociación entre geohelmintos y condiciones socioambientales en diferentes poblaciones humanas de Argentina | Revista Panamericana Salud Publica | -57.9414 | -34.8419 | 0 | Figure 3a |
| Gamboa, María Inés Kozubsky, Leonora Eugenia Costas, María Elena Garraza, Mariela Cardozo, Marta Inés Susevich, María Laura Magistrello, Paula Natalia Navone, Graciela Teresa | 2009 | Asociación entre geohelmintos y condiciones socioambientales en diferentes poblaciones humanas de Argentina | Revista Panamericana Salud Publica | -57.9766 | -35.0121 | 0 | Figure 3a |
| Giraldi, N., Vidotto, O., Navarro, I.T., Garcia, J.L. | 2001 | Enteroparasites prevalence among daycare and elementary school children of municipal schools, Rolandia, PR, Brazil | Revista da Sociedade Brasileira de Medicina Tropical | -51.3773 | -23.3125 | 0.4 | Final validation of the model |
| Girum, Tadesse | 2005 | The prevalence of intestinal helminthic infections and associated risk factors among school children in Babile town, eastern Ethiopia | Ethiopian Journal of Health Development | 42.3311 | 9.2263 | 1 | Design and calibration of the model |
| Glinz, D. Silue, K. D. Knopp, S. Lohourignon, L. K. Yao, K. P. Steinmann, P. Rinaldi, L. Cringoli, G. N'Goran, E. K. Utzinger, J. | 2010 | Comparing diagnostic accuracy of Kato-Katz, Koga agar plate, ether-concentration, and FLOTAC for Schistosoma mansoni and soil-transmitted helminthsComparing diagnostic accuracy of Kato-Katz, Koga agar plate, ether-concentration, and FLOTAC for Schistosoma mansoni and soil-transmitted helminths | PLoS Neglected Tropical DiseasesPLoS Neglected Tropical Diseases | -4.0161 | 5.6028 | 33.9 | Design and calibration of the model |
| Goncalves, J.F. | 1990 | Parasitological and serological studies on amebiasis and other intestinal parasitic infections in the rural sector around Recife, Northeast Brazil | Revista Do Instituto De Medicina Tropical De Sao Paulo | -35.0186 | -8.007 | 5.8 | Final validation of the model |
| Hall, A., Conway, D.J., Anwar, K.S., Rahman, M.L | 1994 | Strongyloides stercoralis in an urban slum community in Bangladesh: factors independently associated with infection | Transactions of the Royal Society of Tropical Medicine and Hygiene | 90.3672 | 23.8209 | 11.6 | Final validation of the model |
| Ibidapo, C. A. Okwa, O. | 2008 | The prevalence and intensity of soil transmitted helminths in a rural community, Lagos suburb, South West Nigeria | International Journal of Agriculture and Biology | 2.8954 | 6.4249 | 18 | Design and calibration of the model |
| Ijagbone, I. F. Olagunju, T. F. | 2006 | Intestinal helminth parasites in school children in Iragbiji, Boripe Local Government, Osun State, Nigeria | African Journal of Biomedical Research | 4.7031 | 7.9006 | 0.6 | Design and calibration of the model |
| Incani, R.N., Ferrer, E., Hoek, D., Ramak, R., Roelfsema, J., Mughini-Gras, L., Kortbeek, T., Pinelli, E | 2017 | Diagnosis of intestinal parasites in a rural community of Venezuela: Advantages and disadvantages of using microscopy or RT-PCR | Acta Tropica | -67.7138 | 9.9684 | 2 | Final validation of the model |
| Kaewpitoon, S. J. Loyd, R. A. Kaewpitoon, N. | 2015 | A Cross-Sectional Survey of Intestinal Helminthiases in Rural Communities of Nakhon Ratchasima Province, Thailand | Journal of the Medical Association of Thailand | 101.5913 | 14.7066 | 1.44 | Design and calibration of the model |
| Khieu, V. Schar, F. Marti, H. Sayasone, S. Duong, S. Muth, S. Odermatt, P. | 2013 | Diagnosis, treatment and risk factors of Strongyloides stercoralis in schoolchildren in Cambodia | PLoS Neglected Tropical Diseases | 105.0276 | 11.2677 | 24.8 | Design and calibration of the model |
| Khieu, V. Schar, F. Marti, H. Sayasone, S. Duong, S. Muth, S. Odermatt, P. | 2013 | Diagnosis, treatment and risk factors of Strongyloides stercoralis in schoolchildren in Cambodia | PLoS Neglected Tropical Diseases | 104.9881 | 11.3599 | 24.8 | Design and calibration of the model |
| Kitvatanachai, S. Boonslip, S. Watanasatitarpa, S. | 2008 | Intestinal parasitic infections in Srimum suburban area of Nakhon Ratchasima Province, Thailand | Tropical Biomedicine | 98.7303 | 8.387 | 3.2 | Design and calibration of the model |
| Kitvatanachai, S. Boonslip, S. Watanasatitarpa, S. | 2008 | Intestinal parasitic infections in Srimum suburban area of Nakhon Ratchasima Province, Thailand | Tropical Biomedicine | 99.0503 | 8.2639 | 3.2 | Design and calibration of the model |
| Kitvatanachai, S. Boonslip, S. Watanasatitarpa, S. | 2008 | Intestinal parasitic infections in Srimum suburban area of Nakhon Ratchasima Province, Thailand | Tropical Biomedicine | 99.2938 | 8.0649 | 3.2 | Design and calibration of the model |
| Kitvatanachai, S. Taylor, A. Rhongbutsri, P. Pongstaporn, W. | 2017 | Determine the prevalence of intestinal and soil-transmitted helminths using different copromicroscopic techniques in Krabi Province, Thailand | Asian Pacific Journal of Tropical Disease | 101.9822 | 14.9981 | 3.3 | Design and calibration of the model |
| Koga-Kita, K. | 2004 | Intestinal parasitic infections and socioeconomic status in Prek Russey Commune, Cambodia | Nippon Koshu Eisei Zasshi - Japanese Journal of Public Health | 104.9441 | 11.4829 | 14.6 | Design and calibration of the model |
| Lemlem, Legesse Berhanu, Erko Asrat, Hailu | 2010 | Current status of intestinal schistosomiasis and soil-transmitted helminthiasis among primary school children in Adwa Town, northern Ethiopia | Ethiopian Journal of Health Development | 38.8889 | 14.1672 | 0 | Figure 3a |
| Ludwig, Karin Maria Ribeiro, André Luiz Teroso Conte, Adriane de Oliveira Campos Decleva, Diego Vinicius Ribeiro, Jéssica Trautwein Diniz | 2012 | Ocorrência de enteroparasitoses na população de um bairro da cidade de Cândido Mota-SP | Journal of the Health Sciences Institute | -50.3835 | -22.7554 | 13 | Design and calibration of the model |
| Machado, E. R. Freitas, C. V. de Costa-Cruz, J. M. | 2010 | Strongyloides stercoralis and other enteroparasites in individuals of rural area of Uberlândia, Minas Gerais State, Brazil | Revista de Patologia Tropical | -48.2755 | -18.9128 | 6.7 | Design and calibration of the model |
| Machado, E. R.Machado, E. R. Souza, T. S. de Costa, J. M. da Costa-Cruz, J. M. | 2008 | Enteroparasites and commensals among individuals living in rural and urban areas in Abadia dos Dourados, Minas Gerais State, Brazil | Sociedad Chilena de Parasitología | -47.4061 | -18.485 | 0 | Figure 3a |
| Marcos, Luis Maco, Vicente Terashima, Angelica Samalvides, Frine Miranda, Elba Gotuzzo, Eduardo | 2003 | Parasitosis intestinal en poblaciones urbana y rural en Sandia, Departamento de Puno, Perú | Parasitología latinoamericana | -69.63 | -14.5745 | 0 | Figure 3a |
| M'Bondoukwe N, P. Kendjo, E. Mawili-Mboumba, D. P. Koumba Lengongo, J. V. Offouga Mbouoronde, C. Nkoghe, D. Toure, F. Bouyou-Akotet, M. K. | 2018 | Prevalence of and risk factors for malaria, filariasis, and intestinal parasites as single infections or co-infections in different settlements of Gabon, Central Africa | Infectious Diseases of Poverty | 12.5456 | -1.8222 | 3.7 | Design and calibration of the model |
| Ngui, R., Halim, N.A.A., Rajoo, Y., Lim, Y. AL, Ambu, S., Rajoo, K., Chang, T.S., Woon, L.C., Mahmud, R | 2016 | Epidemiological Characteristics of Strongyloidiasis in Inhabitants of Indigenous Communities in Borneo Island, Malaysia | The Korean journal of parasitology | 111.6833 | 1.8833 | 2.1 | Final validation of the model |
| Niamnuy, N., Kaewthamasorn, M., Congpuong, K., Phaytanavanh, B., Lohsoonthorn, V. | 2016 | Prevalence and associated risk factors of intestinal parasites in humans and domestic animals across borders of Thailand and Lao PDR: Focus on hookworm and threadworm | The Southeast Asian journal of tropical medicine and public health | 105.447 | 15.0106 | 14 | Final validation of the model |
| Niamnuy, N., Kaewthamasorn, M., Congpuong, K., Phaytanavanh, B., Lohsoonthorn, V. | 2016 | Prevalence and associated risk factors of intestinal parasites in humans and domestic animals across borders of Thailand and Lao PDR: Focus on hookworm and threadworm | The Southeast Asian journal of tropical medicine and public health | 105.4207 | 15.301 | 15.7 | Final validation of the model |
| Nithikathkul, C. Changsap, B. Wannapinyosheep, S. Arnat, N. Kongkham, S. Benchawattananon, R. Leemingsawat, S. | 2003 | Parasitic infections among Karen in Kanchanaburi Province, western Thailand | Southeast Asian Journal of Tropical Medicine & Public Health | 98.4541 | 15.1532 | 7.14 | Design and calibration of the model |
| Ogochukwu, C. O. Patience, O. U. | 2015 | A cross-sectional study of Ascaris lumbricoides infection in a rural community in Ebonyi state, Nigeria: prevalence and risk factors | Iranian Journal of Public Health | 7.7756 | 6.4772 | 3.3 | Design and calibration of the model |
| Olivera, R., Raciny, A., Consuelo, L., Moncada. L., Reyes, H. | 2014 | Detección de Strongyloides stercoralis en Tierralta, Colombia, utilizando cuatro métodos parasitológicos | ." Revista cubana de medicina tropical | -76.0587 | 8.1701 | 1.5 | Final validation of the model |
| Pereira, A.P.M.F., Alencar, M.F.L., Cohen, S.C., Souza-Júnior, P.R.B., Cecchetto, F., Mathias, L.S., Santos, C.P., Almeida, J.C.A., De Moraes Neto, A.H.A. | 2012 | The influence of health education on the prevalence of intestinal parasites in a low-income community of Campos dos Goytacazes, Rio de Janeiro State, Brazil | Parasitology | -41.3112 | -21.6184 | 1.5 | Final validation of the model |
| Pino Santos, A. Nunez Fernandez, F. A. Martinez Sanchez, R. Domenech Canete, I. Rodriguez, M. Jerez Puebla, L. Rodriguez Gonzalez, Z. | 2014 | Prevalence and risk factors for intestinal parasitic infections in a rural community in 'Consolacion del Sur' municipality, Cuba | West Indian Medical Journal | -83.3784 | 22.5536 | 0.67 | Design and calibration of the model |
| Polseela, R. Vitta, A. | 2015 | Prevalence of intestinal parasitic infections among schoolchildren in Phitsanulok Province, Northern Thailand | Asian Pacific Journal of Tropical Disease | 100.2092 | 16.7586 | 2.8 | Design and calibration of the model |
| Popruk, S. Thima, K. Udonsom, R. Rattaprasert, P. Sukthana, Y. | 2011 | Does silent giardia infection need any attention? | Open Tropical Medicine Journal | 100.4928 | 13.7559 | 0.39 | Design and calibration of the model |
| Ribas, A. Jollivet, C. Morand, S. Thongmalayvong, B. Somphavong, S. Siew, C. C. Ting, P. J. Suputtamongkol, S. Saensombath, V. Sanguankiat, S. Tan, B. H. Paboriboune, P. Akkhavong, K. Chaisiri, K. | 2017 | Intestinal Parasitic Infections and Environmental Water Contamination in a Rural Village of Northern Lao PDR | Korean Journal of Parasitology | 102.1678 | 19.8486 | 4.59 | Design and calibration of the model |
| Rocha, R.S., Silva, J.G., Peixoto, S.V., Caldeira, R.L., Firmo, J.O.A., Carvalho, O. dos S., Katz, N | 2000 | Assessment of schistosomiasis and other intestinal parasitoses in school children of the Bambuí municipality, Minas Gerais, Brazil | Revista da Sociedade Brasileira de Medicina Tropical | -45.9795 | -20.0171 | 0.3 | Final validation of the model |
| Roldán, W.H., Espinoza, Y.A., Huapaya, P.E., Huiza, A.F., Sevilla, C.R., Jiménez, S | 2009 | Frequency of human toxocariasis in a rural population from Cajamarca, Peru determined by DOT-ELISA test | Revista do Instituto de Medicina Tropical de Sao Paulo | -78.0833 | -7.5833 | 0.87 | Final validation of the model |
| Salim, N., Schindler, T., Abdul, U., Rothen, J., Genton, B., Lweno, O., Mohammed, A.S., Masimba, J., Kwaba, D., Abdulla, S., Tanner, M., Daubenberger, C., Knopp, S. | 2014 | Enterobiasis and strongyloidiasis and associated co-infections and morbidity markers in infants, preschool- and school-aged children from rural coastal Tanzania: a cross-sectional study | BMC infectious diseases | 38.8986 | -6.4323 | 6.9 | Final validation of the model |
| Sawaya, A.L., Amigo, H., Sigulem, D | 1990 | he risk approach in preschool children suffering malnutrition and intestinal parasitic infection in the city of são paulo, Brazil | Journal of tropical pediatrics | -46.6196 | -23.5808 | 2 | Final validation of the model |
| Sayasone, S. Mak, T. K. Vanmany, M. Rasphone, O. Vounatsou, P. Utzinger, J. Akkhavong, K. Odermatt, P. | 2011 | Helminth and intestinal protozoa infections, multiparasitism and risk factors in Champasack Province, Lao People's Democratic Republic | PLoS Neglected Tropical Diseases | 106.5456 | 15.1863 | 4.6 | Design and calibration of the model |
| Sayasone, S. Mak, T. K. Vanmany, M. Rasphone, O. Vounatsou, P. Utzinger, J. Akkhavong, K. Odermatt, P. | 2011 | Helminth and intestinal protozoa infections, multiparasitism and risk factors in Champasack Province, Lao People's Democratic Republic | PLoS Neglected Tropical Diseases | 105.7681 | 14.0958 | 4.6 | Design and calibration of the model |
| Sayasone, S. Mak, T. K. Vanmany, M. Rasphone, O. Vounatsou, P. Utzinger, J. Akkhavong, K. Odermatt, P. | 2011 | Helminth and intestinal protozoa infections, multiparasitism and risk factors in Champasack Province, Lao People's Democratic Republic | PLoS Neglected Tropical Diseases | 105.7281 | 14.2902 | 4.6 | Design and calibration of the model |
| Sayasone, S. Vonghajack, Y. Vanmany, M. Rasphone, O. Tesana, S. Utzinger, J. Akkhavong, K. Odermatt, P.Sayasone, S. Vonghajack, Y. Vanmany, M. Rasphone, O. Tesana, S. Utzinger, J. Akkhavong, K. Odermatt, P. | 2009 | Diversity of human intestinal helminthiasis in Lao PDR | Transactions of the Royal Society of Tropical Medicine & Hygiene | 102.6326 | 17.9737 | 10.3 | Design and calibration of the model |
| Sayasone, S. Vonghajack, Y. Vanmany, M. Rasphone, O. Tesana, S. Utzinger, J. Akkhavong, K. Odermatt, P.Sayasone, S. Vonghajack, Y. Vanmany, M. Rasphone, O. Tesana, S. Utzinger, J. Akkhavong, K. Odermatt, P. | 2009 | Diversity of human intestinal helminthiasis in Lao PDR | Transactions of the Royal Society of Tropical Medicine & Hygiene | 104.7696 | 16.572 | 10.3 | Design and calibration of the model |
| Schar, F. Inpankaew, T. Traub, R. J. Khieu, V. Dalsgaard, A. Chimnoi, W. Chhoun, C. Sok, D. Marti, H. Muth, S. Odermatt, P. | 2014 | The prevalence and diversity of intestinal parasitic infections in humans and domestic animals in a rural Cambodian village | Parasitology International | 105.1152 | 13.3602 | 24.3 | Design and calibration of the model |
| Senephansiri, P. Laummaunwai, P. Laymanivong, S. Boonmar, T. | 2017 | Status and Risk Factors of Strongyloides stercoralis Infection in Rural Communities of Xayaburi Province, Lao PDR | Korean Journal of Parasitology | 101.3505 | 17.9114 | 44.2 | Design and calibration of the model |
| Sithithaworn, P. Srisawangwong, T. Tesana, S. Daenseekaew, W. Sithithaworn, J. Fujimaki, Y. Ando, K. | 2003 | Epidemiology of Strongyloides stercoralis in north-east Thailand: application of the agar plate culture technique compared with the enzyme-linked immunosorbent assay | Transactions of the Royal Society of Tropical Medicine & Hygiene | 102.6387 | 16.0836 | 28.9 | Design and calibration of the model |
| Sithithaworn, P. Srisawangwong, T. Tesana, S. Daenseekaew, W. Sithithaworn, J. Fujimaki, Y. Ando, K. | 2003 | Epidemiology of Strongyloides stercoralis in north-east Thailand: application of the agar plate culture technique compared with the enzyme-linked immunosorbent assay | Transactions of the Royal Society of Tropical Medicine & Hygiene | 102.4211 | 15.8059 | 28.9 | Design and calibration of the model |
| Sithithaworn, P. Srisawangwong, T. Tesana, S. Daenseekaew, W. Sithithaworn, J. Fujimaki, Y. Ando, K. | 2003 | Epidemiology of Strongyloides stercoralis in north-east Thailand: application of the agar plate culture technique compared with the enzyme-linked immunosorbent assay | Transactions of the Royal Society of Tropical Medicine & Hygiene | 103.22 | 16.6 | 28.9 | Design and calibration of the model |
| Steinmann, P., Yap, P., Utzinger, J., Du, Z.W., Jiang, J.Y., Chen, R., Wu, F.W., Chen, J.X., Zhou, H., Zhou, X.N | 2015 | Control of soil-transmitted helminthiasis in Yunnan province, People’s Republic of China: Experiences and lessons from a 5-year multi-intervention trial | Acta Tropica | 100.3991 | 21.7761 | 3.7 | Final validation of the model |
| Steinmann, P., Yap, P., Utzinger, J., Du, Z.W., Jiang, J.Y., Chen, R., Wu, F.W., Chen, J.X., Zhou, H., Zhou, X.N | 2015 | Control of soil-transmitted helminthiasis in Yunnan province, People’s Republic of China: Experiences and lessons from a 5-year multi-intervention trial | Acta Tropica | 100.3974 | 21.7673 | 7.8 | Final validation of the model |
| Steinmann, P., Yap, P., Utzinger, J., Du, Z.W., Jiang, J.Y., Chen, R., Wu, F.W., Chen, J.X., Zhou, H., Zhou, X.N | 2015 | Control of soil-transmitted helminthiasis in Yunnan province, People’s Republic of China: Experiences and lessons from a 5-year multi-intervention trial | Acta Tropica | 100.3131 | 21.7525 | 13 | Final validation of the model |
| Steinmann, P., Zhou, X.N., Du, Z.W., Jiang, J.Y., Wang, L.B., Wang, X.Z., Li, L.H., Marti, H., Utzinger, J. | 2007 | Occurrence of Strongyloides stercoralis in Yunnan Province, China, and comparison of diagnostic methods | Plos NTD | 100.35 | 21.81 | 11.7 | Final validation of the model |
| Štrkolcová, G., Goldová, M., Bocková, E., Mojžišová, J. | 2017 | The roundworm Strongyloides stercoralis in children, dogs, and soil inside and outside a segregated settlement in Eastern Slovakia: frequent but hardly detectable parasite | Parasitology research | 20.8897 | 48.7039 | 0 | Figure 3a |
| Sultana, Y., Gilbert, G.L., Ahmed, B.N., Lee, R | 2012 | Seroepidemiology of Strongyloides stercoralis in Dhaka, Bangladesh | Parasitology | 90.4035 | 23.7848 | 22.1 | Final validation of the model |
| Sultana, Y., Gilbert, G.L., Ahmed, B.N., Lee, R | 2012 | Strongyloidiasis in a high risk community of Dhaka, Bangladesh | Transactions of the Royal Society of Tropical Medicine and Hygiene | 90.3546 | 23.773 | 61.2 | Final validation of the model |
| Suntaravitun, P. Dokmaikaw, A. | 2017 | Prevalence of intestinal protozoan infections among schoolchildren in Bang Khla District, Chachoengsao Province, Central Thailand | Asian Pacific Journal of Tropical Disease | 101.2183 | 13.8067 | 0.5 | Design and calibration of the model |
| Taranto, N. J. Cajal, S. P. Marzi, M. C. de Fernández, M. M. Frank, F. M. Brú, A. M. Minvielle, M. C. Basualdo, J. A. Malchiodi, E. L. | 2003 | Clinical status and parasitic infection in a Wichí Aboriginal community in Salta, Argentina | Transactions of the Royal Society of Tropical Medicine and Hygiene | -63.7901 | -22.5189 | 50.5 | Design and calibration of the model |
| Téllez, A., Morales, W., Rivera, T., Meyer, E., Leiva, B., Linder, E. | 1997 | Prevalence of intestinal parasites in the human population of Leon, Nicaragua | Acta Tropica | -86.8765 | 12.4367 | 0.3 | Final validation of the model |
| Tilahun, Teklehaymanot | 2009 | Intestinal parasitosis among Kara and Kwego semi-pastoralist tribes in lower Omo Valley, Southwestern Ethiopia | Ethiopian Journal of Health Development | 36.2122 | 5.3805 | 1 | Design and calibration of the model |
| Tork, M. Sharif, M. Charati, J. Y. Nazar, I. Hosseini, S. A. | 2016 | Prevalence of intestinal parasitic infections and associated risk factors in West of Mazandaran Province, Iran. [Persian] | Journal of Mazandaran University of Medical Sciences | 50.643 | 36.9097 | 0.6 | Design and calibration of the model |
| Tork, M. Sharif, M. Charati, J. Y. Nazar, I. Hosseini, S. A. | 2016 | Prevalence of intestinal parasitic infections and associated risk factors in West of Mazandaran Province, Iran. [Persian] | Journal of Mazandaran University of Medical Sciences | 50.8776 | 36.8108 | 0.6 | Design and calibration of the model |
| Tork, M. Sharif, M. Charati, J. Y. Nazar, I. Hosseini, S. A. | 2016 | Prevalence of intestinal parasitic infections and associated risk factors in West of Mazandaran Province, Iran. [Persian] | Journal of Mazandaran University of Medical Sciences | 51.4202 | 36.6557 | 0.6 | Design and calibration of the model |
| Traore, S. G. Odermatt, P. Bonfoh, B. Utzinger, J. Aka, N. D. Adoubryn, K. D. Assoumou, A. Dreyfuss, G. Koussemon, M. | 2011 | No Paragonimus in high-risk groups in Cote d'Ivoire, but considerable prevalence of helminths and intestinal protozoon infections | Parasites and Vectors | -4.3668 | 5.3262 | 0 | Figure 3a |
| Traore, S. G. Odermatt, P. Bonfoh, B. Utzinger, J. Aka, N. D. Adoubryn, K. D. Assoumou, A. Dreyfuss, G. Koussemon, M. | 2011 | No Paragonimus in high-risk groups in Cote d'Ivoire, but considerable prevalence of helminths and intestinal protozoon infections | Parasites and Vectors | -4.0662 | 5.3767 | 0 | Figure 3a |
| Tungtrongchitr, A., Chiworaporn, C., Praewanich, R | 2007 | The potential usefulness of the modified Kato thick smear technique in the detection of intestinal sarcocystosis during field surveys | Southeast Asian journal of tropical medicine and public health | 104.8456 | 15.2519 | 23.1 | Final validation of the model |
| Tuyizere, A. Ndayambaje, A. Walker, T. D. Bayingana, C. Ntirenganya, C. Dusabejambo, V. Hale, D. C. | 2018 | Prevalence of Strongyloides stercoralis infection and other soil-transmitted helminths by cross-sectional survey in a rural community in Gisagara District, Southern Province, Rwanda | Transactions of the Royal Society of Tropical Medicine and Hygiene | 29.8562 | -2.676 | 17.4 | Design and calibration of the model |
| Tuyizere, A. Ndayambaje, A. Walker, T. D. Bayingana, C. Ntirenganya, C. Dusabejambo, V. Hale, D. C. | 2018 | Prevalence of Strongyloides stercoralis infection and other soil-transmitted helminths by cross-sectional survey in a rural community in Gisagara District, Southern Province, Rwanda | Transactions of the Royal Society of Tropical Medicine and Hygiene | 29.9443 | -2.4378 | 17.4 | Design and calibration of the model |
| Ugbomoiko, U. S. Ofoezie, I. E. | 2007 | Multiple infection diagnosis of intestinal helminthiasis in the assessment of health and environmental effect of development projects in Nigeria | Journal of Helminthology | 4.539 | 7.9578 | 1.6 | Design and calibration of the model |
| Vannachone, B | 1998 | An epidemiological survey on intestinal parasite infection in Khammouane Province, Lao PDR, with special reference to strongyloides infection | The Southeast Asian journal of tropical medicine and public health | 104.8043 | 17.4004 | 27.5 | Final validation of the model |
| Verhagen, L.M., Incani, R.N., Franco, C.R., Ugarte, A., Cadenas, Y., Sierra Ruiz, C.I., Hermans, P.W.M., Hoek, D., Campos Ponce, M., de Waard, J.H., Pinelli, E. | 2013 | High Malnutrition Rate in Venezuelan Yanomami Compared to Warao Amerindians and Creoles: Significant Associations WITH Intestinal Parasites and Anemia | PLOS ONE | -60.9781 | 9.0086 | 7 | Final validation of the model |
| Verhagen, L.M., Incani, R.N., Franco, C.R., Ugarte, A., Cadenas, Y., Sierra Ruiz, C.I., Hermans, P.W.M., Hoek, D., Campos Ponce, M., de Waard, J.H., Pinelli, E. | 2013 | High Malnutrition Rate in Venezuelan Yanomami Compared to Warao Amerindians and Creoles: Significant Associations WITH Intestinal Parasites and Anemia | PLOS ONE | -65.5414 | 3.1791 | 24 | Final validation of the model |
| Waree, P. Polseela, P. Pannarunothai, S. Pipitgool, V. | 2001 | THE PRESENT SITUATION OF PARAGONIMIASIS IN ENDEMIC AREA IN PHITSANULOK PROVINCE | Southeast Asian Journal of Tropical Medicine & Public Health | 100.6965 | 16.5604 | 9.59 | Design and calibration of the model |
| Waree, P. Polseela, P. Pannarunothai, S. Pipitgool, V. | 2001 | THE PRESENT SITUATION OF PARAGONIMIASIS IN ENDEMIC AREA IN PHITSANULOK PROVINCE | Southeast Asian Journal of Tropical Medicine & Public Health | 100.7436 | 16.4578 | 9.59 | Design and calibration of the model |
| Warunee, N. Choomanee, L. Sataporn, P. Rapeeporn, Y. Nuttapong, W. Sompong, S. Thongdee, S. Bang-On, S. Rachada, K. | 2007 | Intestinal parasitic infections among school children in Thailand | Tropical Biomedicine | 100.3234 | 13.7937 | 0.05 | Design and calibration of the model |
| Wegayehu, T. Tsalla, T. Seifu, B. Teklu, T. | 2013 | Prevalence of intestinal parasitic infections among highland and lowland dwellers in Gamo area, South Ethiopia | BMC Public Health | 37.6454 | 6.1364 | 5.9 | Design and calibration of the model |
| Widjana, D.P., Sutisna, P | 2000 | Prevalence of soil-transmitted helminth infections in the rural population of Bali, Indonesia | The Southeast Asian journal of tropical medicine and public health | 115.0591 | -8.5562 | 0.9 | Final validation of the model |
| Widjana, D.P., Sutisna, P | 2000 | Prevalence of soil-transmitted helminth infections in the rural population of Bali, Indonesia | The Southeast Asian journal of tropical medicine and public health | 115.3502 | -8.2589 | 1 | Final validation of the model |
| Widjana, D.P., Sutisna, P | 2000 | Prevalence of soil-transmitted helminth infections in the rural population of Bali, Indonesia | The Southeast Asian journal of tropical medicine and public health | 115.2309 | -8.0881 | 1.5 | Final validation of the model |
| Widjana, D.P., Sutisna, P | 2000 | Prevalence of soil-transmitted helminth infections in the rural population of Bali, Indonesia | The Southeast Asian journal of tropical medicine and public health | 115.0813 | -8.5309 | 3.3 | Final validation of the model |
| Wongjindanon, N., Suksrichavalit, T., Subsutti, W., Sarachart, T., Worapisuttiwong, U., Norramatha, P. | 2005 | Current infection rate of Giardia lamblia in two provinces of Thailand | The Southeast Asian journal of tropical medicine and public health | 100.2974 | 13.6638 | 9.7 | Final validation of the model |
| Wongjindanon, N., Suksrichavalit, T., Subsutti, W., Sarachart, T., Worapisuttiwong, U., Norramatha, P. | 2005 | Current infection rate of Giardia lamblia in two provinces of Thailand | The Southeast Asian journal of tropical medicine and public health | 103.5886 | 15.0039 | 9.7 | Final validation of the model |
| Yori, P. P. Kosek, M. Gilman, R. H. Cordova, J. Bern, C. Chavez, C. B. Olortegui, M. P. Montalvan, C. Sanchez, G. M. Worthen, B. Worthen, J. Leung, F. Ore, C. V. | 2006 | Seroepidemiology of strongyloidiasis in the Peruvian Amazon | American Journal of Tropical Medicine & Hygiene | -73.3381 | -3.8023 | 8.7 | Design and calibration of the model |
| Zonta, M.L., Oyhenart, E.E., Navone, G.T. | 2009 | utritional status, body composition, and intestinal parasitism among the mbyá -guaraní communities of misiones, Argentina | American Journal of Human Biology | -55.3447 | -27.4597 | 22.2 | Final validation of the model |
