# Supplementary material for: Mapping the global distribution of Strongyloides stercoralis and hookworms by ecological niche modeling
Source: Parasit Vectors. 2022 Jun 8;15:197. doi: 10.1186/s13071-022-05284-w (PMC9178904; doi:10.1186/s13071-022-05284-w)
Supplement: Supplementary file 2 — Additional file 2: Table S2: Surveys used for the ecological niche model of hookworms. [file 13071_2022_5284_MOESM2_ESM.docx]

# Additional file 2: Table S2: Surveys used for the ecological niche model of hookworms

| **Author** | **Year** | **Title** | **Journal** | **Longitud** | **Latitud** | **Prevalence** | **use of data** |
| --- | --- | --- | --- | --- | --- | --- | --- |
| Faulkner, C. T. Garcia, B. B. Logan, M. H. New, J. C. Patton, S. | 2003 | Prevalence of endoparasitic infection in children and its relation with cholera prevention efforts in Mexico | Pan American Journal of Public Health | -97.8098 | 25.6673 | 0.22 | Design and calibration of the model |
| Aimpun, P. Hshieh, P. | 2004 | Survey for intestinal parasites in Belize, Central America | Southeast Asian Journal of Tropical Medicine & Public Health | -88.9066 | 16.2155 | 55 | Design and calibration of the model |
| Aimpun, P. Hshieh, P. | 2004 | Survey for intestinal parasites in Belize, Central America | Southeast Asian Journal of Tropical Medicine & Public Health | -88.7986 | 16.3474 | 55 | Design and calibration of the model |
| Aimpun, P. Hshieh, P. | 2004 | Survey for intestinal parasites in Belize, Central America | Southeast Asian Journal of Tropical Medicine & Public Health | -88.741 | 16.4411 | 55 | Design and calibration of the model |
| Aimpun, P. Hshieh, P. | 2004 | Survey for intestinal parasites in Belize, Central America | Southeast Asian Journal of Tropical Medicine & Public Health | -88.6255 | 16.4725 | 55 | Design and calibration of the model |
| Cerdas, Carlos Araya, Edna Coto, Susana | 2003 | Parásitos intestinales en la Escuela 15 de agosto, Tirrases de Curridabat, Costa Rica. Mayo-Junio de 2002 | Revista Costarricense de Ciencias Médicas | -84.0247 | 9.9014 | 0.31 | Design and calibration of the model |
| Pino Santos, A. Nunez Fernandez, F. A. Martinez Sanchez, R. Domenech Canete, I. Rodriguez, M. Jerez Puebla, L. Rodriguez Gonzalez, Z. | 2014 | Prevalence and risk factors for intestinal parasitic infections in a rural community in 'Consolacion del Sur' municipality, Cuba | West Indian Medical Journal | -83.3784 | 22.5536 | 2 | Design and calibration of the model |
| Cooper, P. J. Chico, M. E. Gaus, D. Griffin, G. E. | 2003 | Relationship between bacille Calmette-Guerin vaccination, Mantoux test positivity, and geohelminth infection | Transactions of the Royal Society of Tropical Medicine & Hygiene | -78.8922 | 0.0246 | 7.6 | Design and calibration of the model |
| Cooper, P. J. Chico, M. E. Gaus, D. Griffin, G. E. | 2003 | Relationship between bacille Calmette-Guerin vaccination, Mantoux test positivity, and geohelminth infection | Transactions of the Royal Society of Tropical Medicine & Hygiene | -78.4398 | -0.2219 | 7.6 | Design and calibration of the model |
| Yori, P. P. Kosek, M. Gilman, R. H. Cordova, J. Bern, C. Chavez, C. B. Olortegui, M. P. Montalvan, C. Sanchez, G. M. Worthen, B. Worthen, J. Leung, F. Ore, C. V. | 2006 | Seroepidemiology of strongyloidiasis in the Peruvian Amazon | American Journal of Tropical Medicine & Hygiene | -73.3381 | -3.8023 | 4.5 | Design and calibration of the model |
| Bracho M, Angela Rivero-Rodríguez, Zulbey Rios P, Melary Atencio T, Ricardo Villalobos P, Rafael Rodríguez, Luis | 2014 | Parasitosis intestinales en niños y adolescentes de la etnia Yukpa de Toromo, estado Zulia, Venezuela: Comparación de los años 2002 Y 2012 | Kasmera | -72.7138 | 10.0488 | 23.7 | Design and calibration of the model |
| Araujo, Claudio Fernández Fernández, Claudia Leite | 2005 | Prevalência de parasitoses intestinais na cidade de Eirunepé, Amazonas | Revista da Sociedade Brasileira de Medicina Tropical | -69.8681 | -6.6596 | 9.9 | Design and calibration of the model |
| Marcos, Luis Maco, Vicente Terashima, Angelica Samalvides, Frine Miranda, Elba Gotuzzo, Eduardo | 2003 | Parasitosis intestinal en poblaciones urbana y rural en Sandia, Departamento de Puno, Perú | Parasitología latinoamericana | -69.63 | -14.5745 | 0 | Figure 3b |
| Taranto, N. J. Cajal, S. P. Marzi, M. C. de Fernández, M. M. Frank, F. M. Brú, A. M. Minvielle, M. C. Basualdo, J. A. Malchiodi, E. L. | 2003 | Clinical status and parasitic infection in a Wichí Aboriginal community in Salta, Argentina | Transactions of the Royal Society of Tropical Medicine and Hygiene | -63.7901 | -22.5189 | 76 | Design and calibration of the model |
| Gamboa, María Inés Kozubsky, Leonora Eugenia Costas, María Elena Garraza, Mariela Cardozo, Marta Inés Susevich, María Laura Magistrello, Paula Natalia Navone, Graciela Teresa | 2009 | Asociación entre geohelmintos y condiciones socioambientales en diferentes poblaciones humanas de Argentina | Revista Panamericana Salud Publica | -57.9766 | -35.0121 | 0 | Figure 3b |
| Gamboa, María Inés Kozubsky, Leonora Eugenia Costas, María Elena Garraza, Mariela Cardozo, Marta Inés Susevich, María Laura Magistrello, Paula Natalia Navone, Graciela Teresa | 2009 | Asociación entre geohelmintos y condiciones socioambientales en diferentes poblaciones humanas de Argentina | Revista Panamericana Salud Publica | -57.9414 | -34.8419 | 0 | Figure 3b |
| Aguiar, J. I. A. Goncalves, A. Q. Sodre, F. C. Pereira, S. D. R. Boia, M. N. De Lemos, E. R. S. Daher, R. R. | 2007 | Intestinal protozoa and helminths among Terena Indians in the State of Mato Grosso do Sul: High prevalence of Blastocystis hominis | Revista da Sociedade Brasileira de Medicina Tropical | -54.9723 | -20.9262 | 7 | Design and calibration of the model |
| Gamboa, María Inés Kozubsky, Leonora Eugenia Costas, María Elena Garraza, Mariela Cardozo, Marta Inés Susevich, María Laura Magistrello, Paula Natalia Navone, Graciela Teresa | 2009 | Asociación entre geohelmintos y condiciones socioambientales en diferentes poblaciones humanas de Argentina | Revista Panamericana Salud Publica | -54.893 | -27.0964 | 0 | Figure 3b |
| Cardozo Ocampos, G. E. Cañete Duarte, Z. Lenartovicz, V. | 2015 | Frecuencia de enteroparásitos en niños y niñas del primer ciclo de la educación escolar básica de escuelas públicas de ciudad del este, Paraguay | Memorias del Instituto de Investigaciones en Ciencias de la Salud | -54.6162 | -25.5162 | 2 | Design and calibration of the model |
| Carme, B. Motard, A. Bau, P. Day, C. Aznar, C. Moreau, B. | 2002 | Intestinal parasitoses among Wayampi Indians from French Guiana | Parasite | -52.9017 | 2.1969 | 57.1 | Design and calibration of the model |
| Brandelli, C. L. C. Carli, G. A. de Macedo, A. J. Tasca, T. | 2012 | Intestinal parasitism and socio-environmental factors among Mbyá-Guarani indians, Porto Alegre, Rio Grande do Sul, Brazil | Revista do Instituto de Medicina Tropical de São PauloRevista do Instituto de Medicina Tropical de São Paulo | -51.127 | -30.0906 | 6.45 | Design and calibration of the model |
| Machado, E. R. Freitas, C. V. de Costa-Cruz, J. M. | 2010 | Strongyloides stercoralis and other enteroparasites in individuals of rural area of Uberlândia, Minas Gerais State, Brazil | Revista de Patologia Tropical | -48.2755 | -18.9128 | 7.2 | Design and calibration of the model |
| Machado, E. R.Machado, E. R. Souza, T. S. de Costa, J. M. da Costa-Cruz, J. M. | 2008 | Enteroparasites and commensals among individuals living in rural and urban areas in Abadia dos Dourados, Minas Gerais State, Brazil | Sociedad Chilena de Parasitología | -47.4061 | -18.485 | 7.4 | Design and calibration of the model |
| Barbosa, C. V. Barreto, M. M. Andrade, R. J. Sodre, F. d'Avila-Levy, C. M. Peralta, J. M. Igreja, R. P. de Macedo, H. W. Santos, H. L. C. | 2018 | Intestinal parasite infections in a rural community of Rio de Janeiro (Brazil): Prevalence and genetic diversity of Blastocystis subtypes | PLoS ONE | -42.6892 | -22.0461 | 7.1 | Design and calibration of the model |
| Carvalho, Gabriela Lanna Xavier de Moreira, Luciano Evangelista Pena, João Luiz Marinho, Carolina Coimbra Bahia, Maria Terezinha Machado-Coelho, George Luiz Lins | 2012 | A comparative study of the TF-Test®, Kato-Katz, Hoffman-Pons-Janer, Willis and Baermann-Moraes coprologic methods for the detection of human parasitosis | Memórias do Instituto Oswaldo Cruz | -41.2654 | -18.0632 | 8.8 | Design and calibration of the model |
| Assis, E. M. de Olivieria, R. C. de Moreira, L. E. Pena, J. L. Rodrigues, L. C. Machado-Coelho, G. L. L. | 2013 | Prevalence of intestinal parasites in the Maxakali indigenous community in Minas Gerais, Brazil, 2009 | Cadernos de Saúde Pública | -40.5934 | -16.8625 | 37.9 | Design and calibration of the model |
| Fontes, G. Lessa Oliveira, K. K. Lessa Oliveira, A. K. Mauricio da Rocha, E. M. | 2003 | Influence of specific treatment of intestinal parasites and schistosomiasis on prevalence in students in Barra de Santo Antonio, AL. [Portuguese] | Revista da Sociedade Brasileira de Medicina Tropical | -35.508 | -9.4038 | 14 | Design and calibration of the model |
| Becker, S. L. Sieto, B. Silue, K. D. Adjossan, L. Kone, S. Hatz, C. Kern, W. V. N'Goran, E. K. Utzinger, J. | 2011 | Diagnosis, clinical features, and self-reported morbidity of Strongyloides stercoralis and hookworm infection in a Co-endemic setting | PLoS Neglected Tropical Diseases | -5.217 | 6.2328 | 51 | Design and calibration of the model |
| Traore, S. G. Odermatt, P. Bonfoh, B. Utzinger, J. Aka, N. D. Adoubryn, K. D. Assoumou, A. Dreyfuss, G. Koussemon, M. | 2011 | No Paragonimus in high-risk groups in Cote d'Ivoire, but considerable prevalence of helminths and intestinal protozoon infections | Parasites and Vectors | -4.3668 | 5.3262 | 13.3 | Design and calibration of the model |
| Traore, S. G. Odermatt, P. Bonfoh, B. Utzinger, J. Aka, N. D. Adoubryn, K. D. Assoumou, A. Dreyfuss, G. Koussemon, M. | 2011 | No Paragonimus in high-risk groups in Cote d'Ivoire, but considerable prevalence of helminths and intestinal protozoon infections | Parasites and Vectors | -4.0235 | 5.3647 | 13.3 | Design and calibration of the model |
| Glinz, D. Silue, K. D. Knopp, S. Lohourignon, L. K. Yao, K. P. Steinmann, P. Rinaldi, L. Cringoli, G. N'Goran, E. K. Utzinger, J. | 2010 | Comparing diagnostic accuracy of Kato-Katz, Koga agar plate, ether-concentration, and FLOTAC for Schistosoma mansoni and soil-transmitted helminthsComparing diagnostic accuracy of Kato-Katz, Koga agar plate, ether-concentration, and FLOTAC for Schistosoma mansoni and soil-transmitted helminths | PLoS Neglected Tropical DiseasesPLoS Neglected Tropical Diseases | -4.0161 | 5.6028 | 55.4 | Design and calibration of the model |
| Dankwa, K. Addy-Lamptey, P. Latif, A. Essien-Baidoo, S. Ephraim, R. K. D. Gavor-Kwashi, C. E. K. Nuvor, S. V. | 2017 | Intestinal parasitic infections among primary school pupils in Elmina, a fishing community in Ghana | International Journal of Medical and Health Sciences | -1.2706 | 5.1241 | 4.5 | Design and calibration of the model |
| Boko, P. M. Ibikounle, M. Onzo-Aboki, A. Tougoue, J. J. Sissinto, Y. Batcho, W. Kinde-Gazard, D. Kabore, A. | 2016 | Schistosomiasis and Soil Transmitted Helminths Distribution in Benin: A Baseline Prevalence Survey in 30 Districts | PLoS ONE | 0.9067 | 10.4756 | 14.45 | Design and calibration of the model |
| Boko, P. M. Ibikounle, M. Onzo-Aboki, A. Tougoue, J. J. Sissinto, Y. Batcho, W. Kinde-Gazard, D. Kabore, A. | 2016 | Schistosomiasis and Soil Transmitted Helminths Distribution in Benin: A Baseline Prevalence Survey in 30 Districts | PLoS ONE | 0.9161 | 10.7789 | 14.45 | Design and calibration of the model |
| Boko, P. M. Ibikounle, M. Onzo-Aboki, A. Tougoue, J. J. Sissinto, Y. Batcho, W. Kinde-Gazard, D. Kabore, A. | 2016 | Schistosomiasis and Soil Transmitted Helminths Distribution in Benin: A Baseline Prevalence Survey in 30 Districts | PLoS ONE | 0.9919 | 10.0131 | 14.45 | Design and calibration of the model |
| Boko, P. M. Ibikounle, M. Onzo-Aboki, A. Tougoue, J. J. Sissinto, Y. Batcho, W. Kinde-Gazard, D. Kabore, A. | 2016 | Schistosomiasis and Soil Transmitted Helminths Distribution in Benin: A Baseline Prevalence Survey in 30 Districts | PLoS ONE | 1.0039 | 10.8272 | 14.45 | Design and calibration of the model |
| Boko, P. M. Ibikounle, M. Onzo-Aboki, A. Tougoue, J. J. Sissinto, Y. Batcho, W. Kinde-Gazard, D. Kabore, A. | 2016 | Schistosomiasis and Soil Transmitted Helminths Distribution in Benin: A Baseline Prevalence Survey in 30 Districts | PLoS ONE | 1.0083 | 10.5106 | 14.45 | Design and calibration of the model |
| Boko, P. M. Ibikounle, M. Onzo-Aboki, A. Tougoue, J. J. Sissinto, Y. Batcho, W. Kinde-Gazard, D. Kabore, A. | 2016 | Schistosomiasis and Soil Transmitted Helminths Distribution in Benin: A Baseline Prevalence Survey in 30 Districts | PLoS ONE | 1.0269 | 10.6308 | 14.45 | Design and calibration of the model |
| Boko, P. M. Ibikounle, M. Onzo-Aboki, A. Tougoue, J. J. Sissinto, Y. Batcho, W. Kinde-Gazard, D. Kabore, A. | 2016 | Schistosomiasis and Soil Transmitted Helminths Distribution in Benin: A Baseline Prevalence Survey in 30 Districts | PLoS ONE | 1.115 | 10.2231 | 14.45 | Design and calibration of the model |
| Boko, P. M. Ibikounle, M. Onzo-Aboki, A. Tougoue, J. J. Sissinto, Y. Batcho, W. Kinde-Gazard, D. Kabore, A. | 2016 | Schistosomiasis and Soil Transmitted Helminths Distribution in Benin: A Baseline Prevalence Survey in 30 Districts | PLoS ONE | 1.1328 | 10.1264 | 14.45 | Design and calibration of the model |
| Boko, P. M. Ibikounle, M. Onzo-Aboki, A. Tougoue, J. J. Sissinto, Y. Batcho, W. Kinde-Gazard, D. Kabore, A. | 2016 | Schistosomiasis and Soil Transmitted Helminths Distribution in Benin: A Baseline Prevalence Survey in 30 Districts | PLoS ONE | 1.1436 | 10.8106 | 14.45 | Design and calibration of the model |
| Boko, P. M. Ibikounle, M. Onzo-Aboki, A. Tougoue, J. J. Sissinto, Y. Batcho, W. Kinde-Gazard, D. Kabore, A. | 2016 | Schistosomiasis and Soil Transmitted Helminths Distribution in Benin: A Baseline Prevalence Survey in 30 Districts | PLoS ONE | 1.2633 | 10.6158 | 14.45 | Design and calibration of the model |
| Boko, P. M. Ibikounle, M. Onzo-Aboki, A. Tougoue, J. J. Sissinto, Y. Batcho, W. Kinde-Gazard, D. Kabore, A. | 2016 | Schistosomiasis and Soil Transmitted Helminths Distribution in Benin: A Baseline Prevalence Survey in 30 Districts | PLoS ONE | 1.3772 | 10.4967 | 14.45 | Design and calibration of the model |
| Boko, P. M. Ibikounle, M. Onzo-Aboki, A. Tougoue, J. J. Sissinto, Y. Batcho, W. Kinde-Gazard, D. Kabore, A. | 2016 | Schistosomiasis and Soil Transmitted Helminths Distribution in Benin: A Baseline Prevalence Survey in 30 Districts | PLoS ONE | 1.5536 | 10.0544 | 14.45 | Design and calibration of the model |
| Boko, P. M. Ibikounle, M. Onzo-Aboki, A. Tougoue, J. J. Sissinto, Y. Batcho, W. Kinde-Gazard, D. Kabore, A. | 2016 | Schistosomiasis and Soil Transmitted Helminths Distribution in Benin: A Baseline Prevalence Survey in 30 Districts | PLoS ONE | 1.6156 | 10.1997 | 14.45 | Design and calibration of the model |
| Boko, P. M. Ibikounle, M. Onzo-Aboki, A. Tougoue, J. J. Sissinto, Y. Batcho, W. Kinde-Gazard, D. Kabore, A. | 2016 | Schistosomiasis and Soil Transmitted Helminths Distribution in Benin: A Baseline Prevalence Survey in 30 Districts | PLoS ONE | 1.6453 | 8.0161 | 14.45 | Design and calibration of the model |
| Boko, P. M. Ibikounle, M. Onzo-Aboki, A. Tougoue, J. J. Sissinto, Y. Batcho, W. Kinde-Gazard, D. Kabore, A. | 2016 | Schistosomiasis and Soil Transmitted Helminths Distribution in Benin: A Baseline Prevalence Survey in 30 Districts | PLoS ONE | 1.6586 | 8.1589 | 14.45 | Design and calibration of the model |
| Boko, P. M. Ibikounle, M. Onzo-Aboki, A. Tougoue, J. J. Sissinto, Y. Batcho, W. Kinde-Gazard, D. Kabore, A. | 2016 | Schistosomiasis and Soil Transmitted Helminths Distribution in Benin: A Baseline Prevalence Survey in 30 Districts | PLoS ONE | 1.6667 | 6.7625 | 14.45 | Design and calibration of the model |
| Boko, P. M. Ibikounle, M. Onzo-Aboki, A. Tougoue, J. J. Sissinto, Y. Batcho, W. Kinde-Gazard, D. Kabore, A. | 2016 | Schistosomiasis and Soil Transmitted Helminths Distribution in Benin: A Baseline Prevalence Survey in 30 Districts | PLoS ONE | 1.6683 | 6.5825 | 14.45 | Design and calibration of the model |
| Boko, P. M. Ibikounle, M. Onzo-Aboki, A. Tougoue, J. J. Sissinto, Y. Batcho, W. Kinde-Gazard, D. Kabore, A. | 2016 | Schistosomiasis and Soil Transmitted Helminths Distribution in Benin: A Baseline Prevalence Survey in 30 Districts | PLoS ONE | 1.6772 | 6.9406 | 14.45 | Design and calibration of the model |
| Boko, P. M. Ibikounle, M. Onzo-Aboki, A. Tougoue, J. J. Sissinto, Y. Batcho, W. Kinde-Gazard, D. Kabore, A. | 2016 | Schistosomiasis and Soil Transmitted Helminths Distribution in Benin: A Baseline Prevalence Survey in 30 Districts | PLoS ONE | 1.7281 | 10.5639 | 14.45 | Design and calibration of the model |
| Boko, P. M. Ibikounle, M. Onzo-Aboki, A. Tougoue, J. J. Sissinto, Y. Batcho, W. Kinde-Gazard, D. Kabore, A. | 2016 | Schistosomiasis and Soil Transmitted Helminths Distribution in Benin: A Baseline Prevalence Survey in 30 Districts | PLoS ONE | 1.7558 | 10.3811 | 14.45 | Design and calibration of the model |
| Boko, P. M. Ibikounle, M. Onzo-Aboki, A. Tougoue, J. J. Sissinto, Y. Batcho, W. Kinde-Gazard, D. Kabore, A. | 2016 | Schistosomiasis and Soil Transmitted Helminths Distribution in Benin: A Baseline Prevalence Survey in 30 Districts | PLoS ONE | 1.7803 | 7.0981 | 14.45 | Design and calibration of the model |
| Boko, P. M. Ibikounle, M. Onzo-Aboki, A. Tougoue, J. J. Sissinto, Y. Batcho, W. Kinde-Gazard, D. Kabore, A. | 2016 | Schistosomiasis and Soil Transmitted Helminths Distribution in Benin: A Baseline Prevalence Survey in 30 Districts | PLoS ONE | 1.7839 | 6.8047 | 14.45 | Design and calibration of the model |
| Boko, P. M. Ibikounle, M. Onzo-Aboki, A. Tougoue, J. J. Sissinto, Y. Batcho, W. Kinde-Gazard, D. Kabore, A. | 2016 | Schistosomiasis and Soil Transmitted Helminths Distribution in Benin: A Baseline Prevalence Survey in 30 Districts | PLoS ONE | 1.7914 | 6.2747 | 14.45 | Design and calibration of the model |
| Boko, P. M. Ibikounle, M. Onzo-Aboki, A. Tougoue, J. J. Sissinto, Y. Batcho, W. Kinde-Gazard, D. Kabore, A. | 2016 | Schistosomiasis and Soil Transmitted Helminths Distribution in Benin: A Baseline Prevalence Survey in 30 Districts | PLoS ONE | 1.8364 | 6.9792 | 14.45 | Design and calibration of the model |
| Boko, P. M. Ibikounle, M. Onzo-Aboki, A. Tougoue, J. J. Sissinto, Y. Batcho, W. Kinde-Gazard, D. Kabore, A. | 2016 | Schistosomiasis and Soil Transmitted Helminths Distribution in Benin: A Baseline Prevalence Survey in 30 Districts | PLoS ONE | 1.8806 | 7.9825 | 14.45 | Design and calibration of the model |
| Boko, P. M. Ibikounle, M. Onzo-Aboki, A. Tougoue, J. J. Sissinto, Y. Batcho, W. Kinde-Gazard, D. Kabore, A. | 2016 | Schistosomiasis and Soil Transmitted Helminths Distribution in Benin: A Baseline Prevalence Survey in 30 Districts | PLoS ONE | 1.8856 | 8.4175 | 14.45 | Design and calibration of the model |
| Boko, P. M. Ibikounle, M. Onzo-Aboki, A. Tougoue, J. J. Sissinto, Y. Batcho, W. Kinde-Gazard, D. Kabore, A. | 2016 | Schistosomiasis and Soil Transmitted Helminths Distribution in Benin: A Baseline Prevalence Survey in 30 Districts | PLoS ONE | 1.9083 | 6.6231 | 14.45 | Design and calibration of the model |
| Boko, P. M. Ibikounle, M. Onzo-Aboki, A. Tougoue, J. J. Sissinto, Y. Batcho, W. Kinde-Gazard, D. Kabore, A. | 2016 | Schistosomiasis and Soil Transmitted Helminths Distribution in Benin: A Baseline Prevalence Survey in 30 Districts | PLoS ONE | 1.9222 | 6.4033 | 14.45 | Design and calibration of the model |
| Boko, P. M. Ibikounle, M. Onzo-Aboki, A. Tougoue, J. J. Sissinto, Y. Batcho, W. Kinde-Gazard, D. Kabore, A. | 2016 | Schistosomiasis and Soil Transmitted Helminths Distribution in Benin: A Baseline Prevalence Survey in 30 Districts | PLoS ONE | 2.0161 | 7.7733 | 14.45 | Design and calibration of the model |
| Boko, P. M. Ibikounle, M. Onzo-Aboki, A. Tougoue, J. J. Sissinto, Y. Batcho, W. Kinde-Gazard, D. Kabore, A. | 2016 | Schistosomiasis and Soil Transmitted Helminths Distribution in Benin: A Baseline Prevalence Survey in 30 Districts | PLoS ONE | 2.0822 | 7.8961 | 14.45 | Design and calibration of the model |
| Boko, P. M. Ibikounle, M. Onzo-Aboki, A. Tougoue, J. J. Sissinto, Y. Batcho, W. Kinde-Gazard, D. Kabore, A. | 2016 | Schistosomiasis and Soil Transmitted Helminths Distribution in Benin: A Baseline Prevalence Survey in 30 Districts | PLoS ONE | 2.1789 | 8.0131 | 14.45 | Design and calibration of the model |
| Boko, P. M. Ibikounle, M. Onzo-Aboki, A. Tougoue, J. J. Sissinto, Y. Batcho, W. Kinde-Gazard, D. Kabore, A. | 2016 | Schistosomiasis and Soil Transmitted Helminths Distribution in Benin: A Baseline Prevalence Survey in 30 Districts | PLoS ONE | 2.1847 | 8.2231 | 14.45 | Design and calibration of the model |
| Boko, P. M. Ibikounle, M. Onzo-Aboki, A. Tougoue, J. J. Sissinto, Y. Batcho, W. Kinde-Gazard, D. Kabore, A. | 2016 | Schistosomiasis and Soil Transmitted Helminths Distribution in Benin: A Baseline Prevalence Survey in 30 Districts | PLoS ONE | 2.2678 | 10.3342 | 14.45 | Design and calibration of the model |
| Boko, P. M. Ibikounle, M. Onzo-Aboki, A. Tougoue, J. J. Sissinto, Y. Batcho, W. Kinde-Gazard, D. Kabore, A. | 2016 | Schistosomiasis and Soil Transmitted Helminths Distribution in Benin: A Baseline Prevalence Survey in 30 Districts | PLoS ONE | 2.3167 | 8.0789 | 14.45 | Design and calibration of the model |
| Boko, P. M. Ibikounle, M. Onzo-Aboki, A. Tougoue, J. J. Sissinto, Y. Batcho, W. Kinde-Gazard, D. Kabore, A. | 2016 | Schistosomiasis and Soil Transmitted Helminths Distribution in Benin: A Baseline Prevalence Survey in 30 Districts | PLoS ONE | 2.3992 | 10.2781 | 14.45 | Design and calibration of the model |
| Boko, P. M. Ibikounle, M. Onzo-Aboki, A. Tougoue, J. J. Sissinto, Y. Batcho, W. Kinde-Gazard, D. Kabore, A. | 2016 | Schistosomiasis and Soil Transmitted Helminths Distribution in Benin: A Baseline Prevalence Survey in 30 Districts | PLoS ONE | 2.4206 | 11.2872 | 14.45 | Design and calibration of the model |
| Boko, P. M. Ibikounle, M. Onzo-Aboki, A. Tougoue, J. J. Sissinto, Y. Batcho, W. Kinde-Gazard, D. Kabore, A. | 2016 | Schistosomiasis and Soil Transmitted Helminths Distribution in Benin: A Baseline Prevalence Survey in 30 Districts | PLoS ONE | 2.5564 | 9.1256 | 14.45 | Design and calibration of the model |
| Boko, P. M. Ibikounle, M. Onzo-Aboki, A. Tougoue, J. J. Sissinto, Y. Batcho, W. Kinde-Gazard, D. Kabore, A. | 2016 | Schistosomiasis and Soil Transmitted Helminths Distribution in Benin: A Baseline Prevalence Survey in 30 Districts | PLoS ONE | 2.5664 | 9.4508 | 14.45 | Design and calibration of the model |
| Boko, P. M. Ibikounle, M. Onzo-Aboki, A. Tougoue, J. J. Sissinto, Y. Batcho, W. Kinde-Gazard, D. Kabore, A. | 2016 | Schistosomiasis and Soil Transmitted Helminths Distribution in Benin: A Baseline Prevalence Survey in 30 Districts | PLoS ONE | 2.5817 | 9.2772 | 14.45 | Design and calibration of the model |
| Boko, P. M. Ibikounle, M. Onzo-Aboki, A. Tougoue, J. J. Sissinto, Y. Batcho, W. Kinde-Gazard, D. Kabore, A. | 2016 | Schistosomiasis and Soil Transmitted Helminths Distribution in Benin: A Baseline Prevalence Survey in 30 Districts | PLoS ONE | 2.6239 | 9.5625 | 14.45 | Design and calibration of the model |
| Boko, P. M. Ibikounle, M. Onzo-Aboki, A. Tougoue, J. J. Sissinto, Y. Batcho, W. Kinde-Gazard, D. Kabore, A. | 2016 | Schistosomiasis and Soil Transmitted Helminths Distribution in Benin: A Baseline Prevalence Survey in 30 Districts | PLoS ONE | 2.6675 | 9.3733 | 14.45 | Design and calibration of the model |
| Boko, P. M. Ibikounle, M. Onzo-Aboki, A. Tougoue, J. J. Sissinto, Y. Batcho, W. Kinde-Gazard, D. Kabore, A. | 2016 | Schistosomiasis and Soil Transmitted Helminths Distribution in Benin: A Baseline Prevalence Survey in 30 Districts | PLoS ONE | 2.6736 | 10.6761 | 14.45 | Design and calibration of the model |
| Boko, P. M. Ibikounle, M. Onzo-Aboki, A. Tougoue, J. J. Sissinto, Y. Batcho, W. Kinde-Gazard, D. Kabore, A. | 2016 | Schistosomiasis and Soil Transmitted Helminths Distribution in Benin: A Baseline Prevalence Survey in 30 Districts | PLoS ONE | 2.735 | 10.8986 | 14.45 | Design and calibration of the model |
| Boko, P. M. Ibikounle, M. Onzo-Aboki, A. Tougoue, J. J. Sissinto, Y. Batcho, W. Kinde-Gazard, D. Kabore, A. | 2016 | Schistosomiasis and Soil Transmitted Helminths Distribution in Benin: A Baseline Prevalence Survey in 30 Districts | PLoS ONE | 2.7822 | 10.7169 | 14.45 | Design and calibration of the model |
| Boko, P. M. Ibikounle, M. Onzo-Aboki, A. Tougoue, J. J. Sissinto, Y. Batcho, W. Kinde-Gazard, D. Kabore, A. | 2016 | Schistosomiasis and Soil Transmitted Helminths Distribution in Benin: A Baseline Prevalence Survey in 30 Districts | PLoS ONE | 2.8747 | 10.9789 | 14.45 | Design and calibration of the model |
| Ibidapo, C. A. Okwa, O. | 2008 | The prevalence and intensity of soil transmitted helminths in a rural community, Lagos suburb, South West Nigeria | International Journal of Agriculture and Biology | 2.8954 | 6.4249 | 45 | Design and calibration of the model |
| Boko, P. M. Ibikounle, M. Onzo-Aboki, A. Tougoue, J. J. Sissinto, Y. Batcho, W. Kinde-Gazard, D. Kabore, A. | 2016 | Schistosomiasis and Soil Transmitted Helminths Distribution in Benin: A Baseline Prevalence Survey in 30 Districts | PLoS ONE | 2.9222 | 12.2914 | 14.45 | Design and calibration of the model |
| Boko, P. M. Ibikounle, M. Onzo-Aboki, A. Tougoue, J. J. Sissinto, Y. Batcho, W. Kinde-Gazard, D. Kabore, A. | 2016 | Schistosomiasis and Soil Transmitted Helminths Distribution in Benin: A Baseline Prevalence Survey in 30 Districts | PLoS ONE | 2.9364 | 11.1389 | 14.45 | Design and calibration of the model |
| Boko, P. M. Ibikounle, M. Onzo-Aboki, A. Tougoue, J. J. Sissinto, Y. Batcho, W. Kinde-Gazard, D. Kabore, A. | 2016 | Schistosomiasis and Soil Transmitted Helminths Distribution in Benin: A Baseline Prevalence Survey in 30 Districts | PLoS ONE | 3.0331 | 12.2036 | 14.45 | Design and calibration of the model |
| Boko, P. M. Ibikounle, M. Onzo-Aboki, A. Tougoue, J. J. Sissinto, Y. Batcho, W. Kinde-Gazard, D. Kabore, A. | 2016 | Schistosomiasis and Soil Transmitted Helminths Distribution in Benin: A Baseline Prevalence Survey in 30 Districts | PLoS ONE | 3.2069 | 12.0325 | 14.45 | Design and calibration of the model |
| Boko, P. M. Ibikounle, M. Onzo-Aboki, A. Tougoue, J. J. Sissinto, Y. Batcho, W. Kinde-Gazard, D. Kabore, A. | 2016 | Schistosomiasis and Soil Transmitted Helminths Distribution in Benin: A Baseline Prevalence Survey in 30 Districts | PLoS ONE | 3.3758 | 11.1194 | 14.45 | Design and calibration of the model |
| Boko, P. M. Ibikounle, M. Onzo-Aboki, A. Tougoue, J. J. Sissinto, Y. Batcho, W. Kinde-Gazard, D. Kabore, A. | 2016 | Schistosomiasis and Soil Transmitted Helminths Distribution in Benin: A Baseline Prevalence Survey in 30 Districts | PLoS ONE | 3.4222 | 11.8561 | 14.45 | Design and calibration of the model |
| Boko, P. M. Ibikounle, M. Onzo-Aboki, A. Tougoue, J. J. Sissinto, Y. Batcho, W. Kinde-Gazard, D. Kabore, A. | 2016 | Schistosomiasis and Soil Transmitted Helminths Distribution in Benin: A Baseline Prevalence Survey in 30 Districts | PLoS ONE | 3.4878 | 10.9208 | 14.45 | Design and calibration of the model |
| Boko, P. M. Ibikounle, M. Onzo-Aboki, A. Tougoue, J. J. Sissinto, Y. Batcho, W. Kinde-Gazard, D. Kabore, A. | 2016 | Schistosomiasis and Soil Transmitted Helminths Distribution in Benin: A Baseline Prevalence Survey in 30 Districts | PLoS ONE | 3.5169 | 11.7867 | 14.45 | Design and calibration of the model |
| Boko, P. M. Ibikounle, M. Onzo-Aboki, A. Tougoue, J. J. Sissinto, Y. Batcho, W. Kinde-Gazard, D. Kabore, A. | 2016 | Schistosomiasis and Soil Transmitted Helminths Distribution in Benin: A Baseline Prevalence Survey in 30 Districts | PLoS ONE | 3.5839 | 10.7864 | 14.45 | Design and calibration of the model |
| Babatunde, S. K. Adedayo, M. R. Ajiboye, A. E. Sunday, O. Ameen, N. | 2013 | Soil-transmitted helminth infections among school children in rural communities of Moro Local Government Area, Kwara State, Nigeria | African Journal of Microbiology Research | 4.4723 | 8.7082 | 15.4 | Design and calibration of the model |
| Ugbomoiko, U. S. Ofoezie, I. E. | 2007 | Multiple infection diagnosis of intestinal helminthiasis in the assessment of health and environmental effect of development projects in Nigeria | Journal of Helminthology | 4.539 | 7.9578 | 43.7 | Design and calibration of the model |
| Ijagbone, I. F. Olagunju, T. F. | 2006 | Intestinal helminth parasites in school children in Iragbiji, Boripe Local Government, Osun State, Nigeria | African Journal of Biomedical Research | 4.7031 | 7.9006 | 20.5 | Design and calibration of the model |
| Abah, A. E. Arene, F. O. | 2015 | Status of Intestinal Parasitic Infections among Primary School Children in Rivers State, Nigeria | Journal of Parasitology Research | 6.6585 | 5.0828 | 25 | Design and calibration of the model |
| Abah, A. E. Arene, F. O. | 2015 | Status of Intestinal Parasitic Infections among Primary School Children in Rivers State, Nigeria | Journal of Parasitology Research | 6.6638 | 4.6138 | 25 | Design and calibration of the model |
| Abah, A. E. Arene, F. O. | 2015 | Status of Intestinal Parasitic Infections among Primary School Children in Rivers State, Nigeria | Journal of Parasitology Research | 6.8458 | 4.7456 | 25 | Design and calibration of the model |
| Abah, A. E. Arene, F. O. | 2015 | Status of Intestinal Parasitic Infections among Primary School Children in Rivers State, Nigeria | Journal of Parasitology Research | 6.9639 | 4.5699 | 25 | Design and calibration of the model |
| Abah, A. E. Arene, F. O. | 2015 | Status of Intestinal Parasitic Infections among Primary School Children in Rivers State, Nigeria | Journal of Parasitology Research | 7.0283 | 4.8776 | 25 | Design and calibration of the model |
| Abah, A. E. Arene, F. O. | 2015 | Status of Intestinal Parasitic Infections among Primary School Children in Rivers State, Nigeria | Journal of Parasitology Research | 7.1252 | 4.8869 | 25 | Design and calibration of the model |
| Chukwuma, M. C. Ekejindu, I. M. Agbakoba, N. R. Ezeagwuna, D. A. Anaghalu, I. C. Nwosu, D. C. | 2009 | The prevalence and risk factors of geohelminth infections among primary school children in Ebenebe Town, Anambra State, Nigeria | Middle East Journal of Scientific Research | 7.133 | 6.337 | 45.5 | Design and calibration of the model |
| Abah, A. E. Arene, F. O. | 2015 | Status of Intestinal Parasitic Infections among Primary School Children in Rivers State, Nigeria | Journal of Parasitology Research | 7.2437 | 4.7518 | 25 | Design and calibration of the model |
| Abah, A. E. Arene, F. O. | 2015 | Status of Intestinal Parasitic Infections among Primary School Children in Rivers State, Nigeria | Journal of Parasitology Research | 7.2869 | 4.6692 | 25 | Design and calibration of the model |
| Abah, A. E. Arene, F. O. | 2015 | Status of Intestinal Parasitic Infections among Primary School Children in Rivers State, Nigeria | Journal of Parasitology Research | 7.541 | 4.5134 | 25 | Design and calibration of the model |
| Ogochukwu, C. O. Patience, O. U. | 2015 | A cross-sectional study of Ascaris lumbricoides infection in a rural community in Ebonyi state, Nigeria: prevalence and risk factors | Iranian Journal of Public Health | 7.7756 | 6.4772 | 25.5 | Design and calibration of the model |
| Chessed, G. Kwalagbe, B. Furo, N. A. | 2005 | Intestinal helminthiases among school children in Gyawana District, Adamawa State, Nigeria | Global Journal of Pure and Applied Sciences | 11.9215 | 9.5894 | 20.1 | Design and calibration of the model |
| M'Bondoukwe N, P. Kendjo, E. Mawili-Mboumba, D. P. Koumba Lengongo, J. V. Offouga Mbouoronde, C. Nkoghe, D. Toure, F. Bouyou-Akotet, M. K. | 2018 | Prevalence of and risk factors for malaria, filariasis, and intestinal parasites as single infections or co-infections in different settlements of Gabon, Central Africa | Infectious Diseases of Poverty | 12.5456 | -1.8222 | 0.4 | Design and calibration of the model |
| de Alegria, Mlar Colmenares, K. Espasa, M. Amor, A. Lopez, I. Nindia, A. Kanjala, J. Guilherme, D. Sulleiro, E. Barriga, B. Gil, E. Salvador, F. Bocanegra, C. Lopez, T. Moreno, M. Molina, I. | 2017 | Prevalence of Strongyloides stercoralis and Other Intestinal Parasite Infections in School Children in a Rural Area of Angola: A Cross-Sectional Study | American Journal of Tropical Medicine & Hygiene | 14.2419 | -13.0403 | 6.1 | Design and calibration of the model |
| Tuyizere, A. Ndayambaje, A. Walker, T. D. Bayingana, C. Ntirenganya, C. Dusabejambo, V. Hale, D. C. | 2019 | Prevalence of Strongyloides stercoralis infection and other soil-transmitted helminths by cross-sectional survey in a rural community in Gisagara District, Southern Province, Rwanda | Transactions of the Royal Society of Tropical Medicine and Hygiene | 29.8562 | -2.676 | 8.2 | Design and calibration of the model |
| Tuyizere, A. Ndayambaje, A. Walker, T. D. Bayingana, C. Ntirenganya, C. Dusabejambo, V. Hale, D. C. | 2018 | Prevalence of Strongyloides stercoralis infection and other soil-transmitted helminths by cross-sectional survey in a rural community in Gisagara District, Southern Province, Rwanda | Transactions of the Royal Society of Tropical Medicine and Hygiene | 29.9443 | -2.4378 | 8.2 | Design and calibration of the model |
| Randall, A. E. Perez, M. A. Floyd, S. Black, G. F. Crampin, A. C. Ngwira, B. Pistoni, W. N. Mulawa, D. Sichali, L. Mwaungulu, L. Bickle, Q. Fine, P. E. M. | 2002 | Patterns of helminth infection and relationship to BCG vaccination in Karonga District, northern Malawi | Transactions of the Royal Society of Tropical Medicine and Hygiene | 33.9334 | -9.9326 | 63.6 | Design and calibration of the model |
| Easton, A. V. Oliveira, R. G. O'Connell, E. M. Kepha, S. Mwandawiro, C. S. Njenga, S. M. Kihara, J. H. Mwatele, C. Odiere, M. R. Brooker, S. J. Webster, J. P. Anderson, R. M. Nutman, T. B | 2016 | Multi-parallel qPCR provides increased sensitivity and diagnostic breadth for gastrointestinal parasites of humans: field-based inferences on the impact of mass deworming | Parasites & Vectors | 34.5941 | 0.5097 | 18.3 | Design and calibration of the model |
| Tilahun, Teklehaymanot | 2009 | Intestinal parasitosis among Kara and Kwego semi-pastoralist tribes in lower Omo Valley, Southwestern Ethiopia | Ethiopian Journal of Health Development | 36.2122 | 5.3805 | 3.49 | Design and calibration of the model |
| Amor, A. Rodriguez, E. Saugar, J. M. Arroyo, A. Lopez-Quintana, B. Abera, B. Yimer, M. Yizengaw, E. Zewdie, D. Ayehubizu, Z. Hailu, T. Mulu, W. Echazu, A. Krolewieki, A. J. Aparicio, P. Herrador, Z. Anegagrie, M. Benito, A | 2016 | High prevalence of Strongyloides stercoralis in school-aged children in a rural highland of north-western Ethiopia: the role of intensive diagnostic work-up | Parasites & Vectors | 37.2087 | 11.3705 | 54.5 | Design and calibration of the model |
| Amor, A. Rodriguez, E. Saugar, J. M. Arroyo, A. Lopez-Quintana, B. Abera, B. Yimer, M. Yizengaw, E. Zewdie, D. Ayehubizu, Z. Hailu, T. Mulu, W. Echazu, A. Krolewieki, A. J. Aparicio, P. Herrador, Z. Anegagrie, M. Benito, A | 2016 | High prevalence of Strongyloides stercoralis in school-aged children in a rural highland of north-western Ethiopia: the role of intensive diagnostic work-up | Parasites & Vectors | 37.2831 | 11.4731 | 54.5 | Design and calibration of the model |
| Amor, A. Rodriguez, E. Saugar, J. M. Arroyo, A. Lopez-Quintana, B. Abera, B. Yimer, M. Yizengaw, E. Zewdie, D. Ayehubizu, Z. Hailu, T. Mulu, W. Echazu, A. Krolewieki, A. J. Aparicio, P. Herrador, Z. Anegagrie, M. Benito, A | 2016 | High prevalence of Strongyloides stercoralis in school-aged children in a rural highland of north-western Ethiopia: the role of intensive diagnostic work-up | Parasites & Vectors | 37.3546 | 11.707 | 54.5 | Design and calibration of the model |
| Bayeh, Abera Genetu, Alem Mulat, Yimer Herrador, Z. | 2013 | Epidemiology of soil-transmitted helminths, schistosoma mansoni, and haematocrit values among schoolchildren in Ethiopia | Journal of Infection in Developing Countries | 37.3795 | 11.5726 | 28.8 | Design and calibration of the model |
| Wegayehu, T. Tsalla, T. Seifu, B. Teklu, T. | 2013 | Prevalence of intestinal parasitic infections among highland and lowland dwellers in Gamo area, South Ethiopia | BMC Public Health | 37.5742 | 6.1931 | 4.9 | Design and calibration of the model |
| Amor, A. Rodriguez, E. Saugar, J. M. Arroyo, A. Lopez-Quintana, B. Abera, B. Yimer, M. Yizengaw, E. Zewdie, D. Ayehubizu, Z. Hailu, T. Mulu, W. Echazu, A. Krolewieki, A. J. Aparicio, P. Herrador, Z. Anegagrie, M. Benito, A | 2016 | High prevalence of Strongyloides stercoralis in school-aged children in a rural highland of north-western Ethiopia: the role of intensive diagnostic work-up | Parasites & Vectors | 37.5834 | 11.4857 | 54.5 | Design and calibration of the model |
| Wegayehu, T. Tsalla, T. Seifu, B. Teklu, T. | 2013 | Prevalence of intestinal parasitic infections among highland and lowland dwellers in Gamo area, South Ethiopia | BMC Public Health | 37.6454 | 6.1364 | 4.9 | Design and calibration of the model |
| Lemlem, Legesse Berhanu, Erko Asrat, Hailu | 2010 | Current status of intestinal schistosomiasis and soil-transmitted helminthiasis among primary school children in Adwa Town, northern Ethiopia | Ethiopian Journal of Health Development | 38.8889 | 14.1672 | 1 | Design and calibration of the model |
| Girum, Tadesse | 2005 | The prevalence of intestinal helminthic infections and associated risk factors among school children in Babile town, eastern Ethiopia | Ethiopian Journal of Health Development | 42.3311 | 9.2263 | 6.15 | Design and calibration of the model |
| Alsubaie, A. S. R. Azazy, A. A. Omer, E. O. Al-Shibani, L. A. Al-Mekhlafi, A. Q. Al-Khawlani, F. A. | 2016 | Pattern of parasitic infections as public health problem among school children: A comparative study between rural and urban areas | Journal of Taibah University Medical Sciences | 44.1651 | 13.9693 | 1.2 | Design and calibration of the model |
| Tork, M. Sharif, M. Charati, J. Y. Nazar, I. Hosseini, S. A. | 2016 | Prevalence of intestinal parasitic infections and associated risk factors in West of Mazandaran Province, Iran. [Persian] | Journal of Mazandaran University of Medical Sciences | 50.6425 | 36.9146 | 0.1 | Design and calibration of the model |
| Tork, M. Sharif, M. Charati, J. Y. Nazar, I. Hosseini, S. A. | 2016 | Prevalence of intestinal parasitic infections and associated risk factors in West of Mazandaran Province, Iran. [Persian] | Journal of Mazandaran University of Medical Sciences | 50.8776 | 36.8108 | 0.1 | Design and calibration of the model |
| Tork, M. Sharif, M. Charati, J. Y. Nazar, I. Hosseini, S. A. | 2016 | Prevalence of intestinal parasitic infections and associated risk factors in West of Mazandaran Province, Iran. [Persian] | Journal of Mazandaran University of Medical Sciences | 51.4202 | 36.6557 | 0.1 | Design and calibration of the model |
| Devi, U. Borkakoty, B. Mahanta, J. | 2011 | Strongyloidiasis in Assam, India: A community-based study | Tropical Parasitology | 94.9113 | 27.4729 | 7.1 | Design and calibration of the model |
| Nithikathkul, C. Changsap, B. Wannapinyosheep, S. Arnat, N. Kongkham, S. Benchawattananon, R. Leemingsawat, S. | 2003 | Parasitic infections among Karen in Kanchanaburi Province, western Thailand | Southeast Asian Journal of Tropical Medicine & Public Health | 98.4541 | 15.1532 | 17.1 | Design and calibration of the model |
| Kitvatanachai, S. Boonslip, S. Watanasatitarpa, S. | 2008 | Intestinal parasitic infections in Srimum suburban area of Nakhon Ratchasima Province, Thailand | Tropical Biomedicine | 98.7303 | 8.387 | 8.1 | Design and calibration of the model |
| Kitvatanachai, S. Boonslip, S. Watanasatitarpa, S. | 2008 | Intestinal parasitic infections in Srimum suburban area of Nakhon Ratchasima Province, Thailand | Tropical Biomedicine | 99.0503 | 8.2639 | 8.1 | Design and calibration of the model |
| Kitvatanachai, S. Boonslip, S. Watanasatitarpa, S. | 2008 | Intestinal parasitic infections in Srimum suburban area of Nakhon Ratchasima Province, Thailand | Tropical Biomedicine | 99.2938 | 8.0649 | 8.1 | Design and calibration of the model |
| Polseela, R. Vitta, A. | 2015 | Prevalence of intestinal parasitic infections among schoolchildren in Phitsanulok Province, Northern Thailand | Asian Pacific Journal of Tropical Disease | 100.2188 | 16.7496 | 0.9 | Design and calibration of the model |
| Warunee, N. Choomanee, L. Sataporn, P. Rapeeporn, Y. Nuttapong, W. Sompong, S. Thongdee, S. Bang-On, S. Rachada, K. | 2007 | Intestinal parasitic infections among school children in Thailand | Tropical Biomedicine | 100.3234 | 13.7937 | 0.05 | Design and calibration of the model |
| Popruk, S. Thima, K. Udonsom, R. Rattaprasert, P. Sukthana, Y. | 2011 | Does silent giardia infection need any attention? | Open Tropical Medicine Journal | 100.4803 | 13.7712 | 1.82 | Design and calibration of the model |
| Waree, P. Polseela, P. Pannarunothai, S. Pipitgool, V. | 2001 | THE PRESENT SITUATION OF PARAGONIMIASIS IN ENDEMIC AREA IN PHITSANULOK PROVINCE | Southeast Asian Journal of Tropical Medicine & Public Health | 100.6965 | 16.5604 | 8.22 | Design and calibration of the model |
| Waree, P. Polseela, P. Pannarunothai, S. Pipitgool, V. | 2001 | THE PRESENT SITUATION OF PARAGONIMIASIS IN ENDEMIC AREA IN PHITSANULOK PROVINCE | Southeast Asian Journal of Tropical Medicine & Public Health | 100.7436 | 16.4578 | 8.22 | Design and calibration of the model |
| Suntaravitun, P. Dokmaikaw, A. | 2017 | Prevalence of intestinal protozoan infections among schoolchildren in Bang Khla District, Chachoengsao Province, Central Thailand | Asian Pacific Journal of Tropical Disease | 101.2183 | 13.8067 | 0.5 | Design and calibration of the model |
| Senephansiri, P. Laummaunwai, P. Laymanivong, S. Boonmar, T. | 2017 | Status and Risk Factors of Strongyloides stercoralis Infection in Rural Communities of Xayaburi Province, Lao PDR | Korean Journal of Parasitology | 101.3505 | 17.9114 | 17.1 | Design and calibration of the model |
| Kaewpitoon, S. J. Loyd, R. A. Kaewpitoon, N. | 2015 | A Cross-Sectional Survey of Intestinal Helminthiases in Rural Communities of Nakhon Ratchasima Province, Thailand | Journal of the Medical Association of Thailand | 101.5913 | 14.7066 | 4.31 | Design and calibration of the model |
| Kitvatanachai, S. Boonslip, S. Watanasatitarpa, S. | 2008 | Intestinal parasitic infections in Srimum suburban area of Nakhon Ratchasima Province, Thailand | Tropical Biomedicine | 101.9822 | 14.9981 | 8.1 | Design and calibration of the model |
| Conlan, J. V. Khamlome, B. Vongxay, K. Elliot, A. Pallant, L. Sripa, B. Blacksell, S. D. Fenwick, S. Thompson, R. C | 2012 | Soil-transmitted helminthiasis in Laos: a community-wide cross-sectional study of humans and dogs in a mass drug administration environment | American Journal of Tropical Medicine & Hygiene | 101.9856 | 20.6902 | 46.3 | Design and calibration of the model |
| Ribas, A. Jollivet, C. Morand, S. Thongmalayvong, B. Somphavong, S. Siew, C. C. Ting, P. J. Suputtamongkol, S. Saensombath, V. Sanguankiat, S. Tan, B. H. Paboriboune, P. Akkhavong, K. Chaisiri, K. | 2017 | Intestinal Parasitic Infections and Environmental Water Contamination in a Rural Village of Northern Lao PDR | Korean Journal of Parasitology | 102.1678 | 19.8486 | 63.3 | Design and calibration of the model |
| Conlan, J. V. Khamlome, B. Vongxay, K. Elliot, A. Pallant, L. Sripa, B. Blacksell, S. D. Fenwick, S. Thompson, R. C | 2013 | Soil-transmitted helminthiasis in Laos: a community-wide cross-sectional study of humans and dogs in a mass drug administration environment | American Journal of Tropical Medicine & Hygiene | 102.1832 | 19.7589 | 46.3 | Design and calibration of the model |
| Sithithaworn, P. Srisawangwong, T. Tesana, S. Daenseekaew, W. Sithithaworn, J. Fujimaki, Y. Ando, K. | 2003 | Epidemiology of Strongyloides stercoralis in north-east Thailand: application of the agar plate culture technique compared with the enzyme-linked immunosorbent assay | Transactions of the Royal Society of Tropical Medicine & Hygiene | 102.4211 | 15.8059 | 12.3 | Design and calibration of the model |
| Sayasone, S. Vonghajack, Y. Vanmany, M. Rasphone, O. Tesana, S. Utzinger, J. Akkhavong, K. Odermatt, P.Sayasone, S. Vonghajack, Y. Vanmany, M. Rasphone, O. Tesana, S. Utzinger, J. Akkhavong, K. Odermatt, P. | 2009 | Diversity of human intestinal helminthiasis in Lao PDR | Transactions of the Royal Society of Tropical Medicine & Hygiene | 102.6326 | 17.9737 | 65.9 | Design and calibration of the model |
| Sithithaworn, P. Srisawangwong, T. Tesana, S. Daenseekaew, W. Sithithaworn, J. Fujimaki, Y. Ando, K. | 2003 | Epidemiology of Strongyloides stercoralis in north-east Thailand: application of the agar plate culture technique compared with the enzyme-linked immunosorbent assay | Transactions of the Royal Society of Tropical Medicine & Hygiene | 102.6387 | 16.0836 | 12.3 | Design and calibration of the model |
| Boonjaraspinyo, S. Boonmars, T. Kaewsamut, B. Ekobol, N. Laummaunwai, P. Aukkanimart, R. Wonkchalee, N. Juasook, A. Sriraj, P. | 2013 | A cross-sectional study on intestinal parasitic infections in rural communities, northeast Thailand | Korean Journal of Parasitology | 102.8236 | 16.4322 | 0.4 | Design and calibration of the model |
| Sithithaworn, P. Srisawangwong, T. Tesana, S. Daenseekaew, W. Sithithaworn, J. Fujimaki, Y. Ando, K. | 2003 | Epidemiology of Strongyloides stercoralis in north-east Thailand: application of the agar plate culture technique compared with the enzyme-linked immunosorbent assay | Transactions of the Royal Society of Tropical Medicine & Hygiene | 103.22 | 16.6 | 12.3 | Design and calibration of the model |
| Conlan, J. V. Khamlome, B. Vongxay, K. Elliot, A. Pallant, L. Sripa, B. Blacksell, S. D. Fenwick, S. Thompson, R. C | 2014 | Soil-transmitted helminthiasis in Laos: a community-wide cross-sectional study of humans and dogs in a mass drug administration environment | American Journal of Tropical Medicine & Hygiene | 104.2274 | 20.4096 | 46.3 | Design and calibration of the model |
| Forrer, A. Khieu, V. Schar, F. Vounatsou, P. Chammartin, F. Marti, H. Muth, S. Odermatt, P. | 2019 | Strongyloides stercoralis and hookworm co-infection: spatial distribution and determinants in Preah Vihear Province, Cambodia | Parasites & Vectors | 104.6988 | 13.819 | 49 | Design and calibration of the model |
| Sayasone, S. Vonghajack, Y. Vanmany, M. Rasphone, O. Tesana, S. Utzinger, J. Akkhavong, K. Odermatt, P.Sayasone, S. Vonghajack, Y. Vanmany, M. Rasphone, O. Tesana, S. Utzinger, J. Akkhavong, K. Odermatt, P. | 2009 | Diversity of human intestinal helminthiasis in Lao PDR | Transactions of the Royal Society of Tropical Medicine & Hygiene | 104.7696 | 16.572 | 65.9 | Design and calibration of the model |
| Forrer, A. Khieu, V. Schar, F. Vounatsou, P. Chammartin, F. Marti, H. Muth, S. Odermatt, P. | 2019 | Strongyloides stercoralis and hookworm co-infection: spatial distribution and determinants in Preah Vihear Province, Cambodia | Parasites & Vectors | 104.9012 | 13.5786 | 49 | Design and calibration of the model |
| Forrer, A. Khieu, V. Schar, F. Vounatsou, P. Chammartin, F. Marti, H. Muth, S. Odermatt, P. | 2019 | Strongyloides stercoralis and hookworm co-infection: spatial distribution and determinants in Preah Vihear Province, Cambodia | Parasites & Vectors | 104.941 | 14.2174 | 49 | Design and calibration of the model |
| Koga-Kita, K. | 2004 | Intestinal parasitic infections and socioeconomic status in Prek Russey Commune, Cambodia | Nippon Koshu Eisei Zasshi - Japanese Journal of Public Health | 104.9441 | 11.4829 | 21.8 | Design and calibration of the model |
| Khieu, V. Schar, F. Marti, H. Sayasone, S. Duong, S. Muth, S. Odermatt, P. | 2013 | Diagnosis, treatment and risk factors of Strongyloides stercoralis in schoolchildren in Cambodia | PLoS Neglected Tropical Diseases | 104.9881 | 11.3599 | 31.4 | Design and calibration of the model |
| Khieu, V. Schar, F. Marti, H. Sayasone, S. Duong, S. Muth, S. Odermatt, P. | 2013 | Diagnosis, treatment and risk factors of Strongyloides stercoralis in schoolchildren in Cambodia | PLoS Neglected Tropical Diseases | 105.0276 | 11.2677 | 51.4 | Design and calibration of the model |
| Forrer, A. Khieu, V. Schar, F. Vounatsou, P. Chammartin, F. Marti, H. Muth, S. Odermatt, P. | 2018 | Strongyloides stercoralis and hookworm co-infection: spatial distribution and determinants in Preah Vihear Province, Cambodia | Parasites & Vectors | 105.0356 | 13.8556 | 49 | Design and calibration of the model |
| Schar, F. Inpankaew, T. Traub, R. J. Khieu, V. Dalsgaard, A. Chimnoi, W. Chhoun, C. Sok, D. Marti, H. Muth, S. Odermatt, P. | 2014 | The prevalence and diversity of intestinal parasitic infections in humans and domestic animals in a rural Cambodian village | Parasitology International | 105.1152 | 13.3602 | 63.3 | Design and calibration of the model |
| Erlanger, T. E. Sayasone, S. Krieger, G. R. Kaul, S. Sananikhom, P. Tanner, M. Odermatt, P. Utzinger, J. | 2008 | Baseline health situation of communities affected by the Nam Theun 2 hydroelectric project in central Lao PDR and indicators for monitoring | International Journal of Environmental Health Research | 105.1881 | 17.6975 | 9.7 | Design and calibration of the model |
| Forrer, A. Khieu, V. Schar, F. Vounatsou, P. Chammartin, F. Marti, H. Muth, S. Odermatt, P. | 2019 | Strongyloides stercoralis and hookworm co-infection: spatial distribution and determinants in Preah Vihear Province, Cambodia | Parasites & Vectors | 105.2576 | 13.6537 | 49 | Design and calibration of the model |
| Sayasone, S. Mak, T. K. Vanmany, M. Rasphone, O. Vounatsou, P. Utzinger, J. Akkhavong, K. Odermatt, P. | 2013 | Helminth and intestinal protozoa infections, multiparasitism and risk factors in Champasack Province, Lao People's Democratic Republic | PLoS Neglected Tropical Diseases | 105.7281 | 14.2902 | 76.8 | Design and calibration of the model |
| Sayasone, S. Mak, T. K. Vanmany, M. Rasphone, O. Vounatsou, P. Utzinger, J. Akkhavong, K. Odermatt, P. | 2011 | Helminth and intestinal protozoa infections, multiparasitism and risk factors in Champasack Province, Lao People's Democratic Republic | PLoS Neglected Tropical Diseases | 105.7681 | 14.0958 | 76.8 | Design and calibration of the model |
| Tang, N. Luo, N. J. | 2003 | A cross-sectional study of intestinal parasitic infections in a rural district of west China | Canadian Journal of Infectious Diseases | 106.3991 | 29.8047 | 17.71 | Design and calibration of the model |
| Sayasone, S. Mak, T. K. Vanmany, M. Rasphone, O. Vounatsou, P. Utzinger, J. Akkhavong, K. Odermatt, P. | 2011 | Helminth and intestinal protozoa infections, multiparasitism and risk factors in Champasack Province, Lao People's Democratic Republic | PLoS Neglected Tropical Diseases | 106.5456 | 15.1863 | 76.8 | Design and calibration of the model |
| Ahmad; A. F. Ngui; R. Muhammad Aidil; R. Lim; Y. A. Rohela; M. | 2014 | Tropical Biomedicine |  | 100.5907 | 4.2173 | 0.763 | Final validation of the model |
| Appleton; C. C. Mosala; T. I. Levin; J. Olsen; A. | 2009 | Geohelminth infection and re-infection after chemotherapy among slum-dwelling children in Durban; South Africa | Annals of Tropical Medicine and Parasitology | 30.88 | -29.9 | 0 | Figure 3b |
| Appleton; C. C. Mosala; T. I. Levin; J. Olsen; A. | 2009 | Geohelminth infection and re-infection after chemotherapy among slum-dwelling children in Durban; South Africa | Annals of Tropical Medicine and Parasitology | 31 | -29.8 | 0 | Figure 3b |
| Appleton; C. C. Mosala; T. I. Levin; J. Olsen; A. | 2009 | Geohelminth infection and re-infection after chemotherapy among slum-dwelling children in Durban; South Africa | Annals of Tropical Medicine and Parasitology | 30.98 | -29.81 | 0.82 | Final validation of the model |
| Appleton; C. C. Mosala; T. I. Levin; J. Olsen; A. | 2009 | Geohelminth infection and re-infection after chemotherapy among slum-dwelling children in Durban; South Africa | Annals of Tropical Medicine and Parasitology | 30.97 | -29.81 | 1.639 | Final validation of the model |
| Appleton; C. C. Mosala; T. I. Levin; J. Olsen; A. | 2009 | Geohelminth infection and re-infection after chemotherapy among slum-dwelling children in Durban; South Africa | Annals of Tropical Medicine and Parasitology | 30.94 | -29.79 | 3.077 | Final validation of the model |
| Appleton; C. C. Mosala; T. I. Levin; J. Olsen; A. | 2009 | Geohelminth infection and re-infection after chemotherapy among slum-dwelling children in Durban; South Africa | Annals of Tropical Medicine and Parasitology | 30.86 | -29.91 | 4.286 | Final validation of the model |
| Appleton; C. C. Mosala; T. I. Levin; J. Olsen; A. | 2009 | Geohelminth infection and re-infection after chemotherapy among slum-dwelling children in Durban; South Africa | Annals of Tropical Medicine and Parasitology | 30.97 | -29.8 | 6.098 | Final validation of the model |
| Appleton; C. C. Mosala; T. I. Levin; J. Olsen; A. | 2009 | Geohelminth infection and re-infection after chemotherapy among slum-dwelling children in Durban; South Africa | Annals of Tropical Medicine and Parasitology | 31.01 | -29.79 | 8.451 | Final validation of the model |
| Appleton; C. C. Mosala; T. I. Levin; J. Olsen; A. | 2009 | Geohelminth infection and re-infection after chemotherapy among slum-dwelling children in Durban; South Africa | Annals of Tropical Medicine and Parasitology | 31.01 | -29.77 | 17.886 | Final validation of the model |
| Appleton; C. C. Mosala; T. I. Levin; J. Olsen; A. | 2009 | Geohelminth infection and re-infection after chemotherapy among slum-dwelling children in Durban; South Africa | Annals of Tropical Medicine and Parasitology | 31 | -29.79 | 20.69 | Final validation of the model |
| Korzeniewski K; Augustynowicz A; Smolen A; Lass A | 2015 | Epidemiology of intestinal parasitic infections in school children in Ghazni Province; eastern Afghanistan | Pakistan Journal of Medical Sciences | 68.42096 | 33.55391 | 1.495 | Final validation of the model |
| Laymanivong; S. Hangvanthong; B. Insisiengmay; B. Vanisaveth; V. Laxachack; P. Jongthawin; J. Sanpool; O. Thanchomnang; T. Sadaow; L. Phosuk; I. Rodpai; R. Maleewong; W. Intapan; P. M. | 2016 | First molecular identification and report of genetic diversity of Strongyloides stercoralis; a current major soil-transmitted helminth in humans from Lao People's Democratic Republic | Parasitology Research | 102.304217 | 20.137898 | 30.579 | Final validation of the model |
| Midzi; N. Mtapuri-Zinyowera; S. Mapingure; M. P. Paul; N. H. Sangweme; D. Hlerema; G. Mutsaka; M. J. Tongogara; F. Makware; G. Chadukura; V. Brouwer; K. C. Mutapi; F. Kumar; N. Mduluza; T. | 2011 | Knowledge attitudes and practices of grade three primary schoolchildren in relation to schistosomiasis; soil transmitted helminthiasis and malaria in Zimbabwe | BMC Infectious Diseases | 31.533757 | -17.027366 | 0 | Figure 3b |
| Midzi; N. Mtapuri-Zinyowera; S. Mapingure; M. P. Paul; N. H. Sangweme; D. Hlerema; G. Mutsaka; M. J. Tongogara; F. Makware; G. Chadukura; V. Brouwer; K. C. Mutapi; F. Kumar; N. Mduluza; T. | 2011 | Knowledge attitudes and practices of grade three primary schoolchildren in relation to schistosomiasis; soil transmitted helminthiasis and malaria in Zimbabwe | BMC Infectious Diseases | 31.66667 | -17.16667 | 5.4 | Final validation of the model |
| Midzi; N. Mtapuri-Zinyowera; S. Mapingure; M. P. Paul; N. H. Sangweme; D. Hlerema; G. Mutsaka; M. J. Tongogara; F. Makware; G. Chadukura; V. Brouwer; K. C. Mutapi; F. Kumar; N. Mduluza; T. | 2011 | Knowledge attitudes and practices of grade three primary schoolchildren in relation to schistosomiasis; soil transmitted helminthiasis and malaria in Zimbabwe | BMC Infectious Diseases | 31.66667 | -17.16667 | 10.5 | Final validation of the model |
| Midzi; N. Mtapuri-Zinyowera; S. Mapingure; M. P. Paul; N. H. Sangweme; D. Hlerema; G. Mutsaka; M. J. Tongogara; F. Makware; G. Chadukura; V. Brouwer; K. C. Mutapi; F. Kumar; N. Mduluza; T. | 2011 | Knowledge attitudes and practices of grade three primary schoolchildren in relation to schistosomiasis; soil transmitted helminthiasis and malaria in Zimbabwe | BMC Infectious Diseases | 31.66667 | -17.16667 | 29.6 | Final validation of the model |
| Njunda; A. L. Fon; S. G. Assob; J. C. Nsagha; D. S. Kwenti; T. D. Kwenti; T. E. | 2015 | Coinfection with malaria and intestinal parasites; and its association with anaemia in children in Cameroon | Infectious Diseases of Poverty | 9.640833 | 4.721667 | 8.163 | Final validation of the model |
| Sacko; M. Magnussen; P. Keita; A. D. Traore; M. S. Landoure; A. Doucoure; A. Madsen; H. Vennervald; B. J. | 2011 | Impact of Schistosoma haematobium infection on urinary tract pathology; nutritional status and anaemia in school-aged children in two different endemic areas of the Niger River Basin; Mali | Acta Tropica | -8.233234 | 11.616613 | 4.054 | Final validation of the model |
| Sacko; M. Magnussen; P. Keita; A. D. Traore; M. S. Landoure; A. Doucoure; A. Madsen; H. Vennervald; B. J. | 2011 | Impact of Schistosoma haematobium infection on urinary tract pathology; nutritional status and anaemia in school-aged children in two different endemic areas of the Niger River Basin; Mali | Acta Tropica | -7.559894 | 12.866163 | 6.81 | Final validation of the model |
| Salim; N. Knopp; S. Lweno; O. Abdul; U. Mohamed; A. Schindler; T. Rothen; J. Masimba; J. Kwaba; D. Mohammed; A. S. Althaus; F. Abdulla; S. Tanner; M. Daubenberger; C. Genton; B. | 2015 | Distribution and risk factors for Plasmodium and helminth co-infections: a cross-sectional survey among children in Bagamoyo district; coastal region of Tanzania | Plos NTD | -6.1301 | 38.46817 | 0 | Figure 3b |
| Salim; N. Knopp; S. Lweno; O. Abdul; U. Mohamed; A. Schindler; T. Rothen; J. Masimba; J. Kwaba; D. Mohammed; A. S. Althaus; F. Abdulla; S. Tanner; M. Daubenberger; C. Genton; B. | 2015 | Distribution and risk factors for Plasmodium and helminth co-infections: a cross-sectional survey among children in Bagamoyo district; coastal region of Tanzania | Plos NTD | -6.14559 | 38.429218 | 0 | Figure 3b |
| Salim; N. Knopp; S. Lweno; O. Abdul; U. Mohamed; A. Schindler; T. Rothen; J. Masimba; J. Kwaba; D. Mohammed; A. S. Althaus; F. Abdulla; S. Tanner; M. Daubenberger; C. Genton; B. | 2015 | Distribution and risk factors for Plasmodium and helminth co-infections: a cross-sectional survey among children in Bagamoyo district; coastal region of Tanzania | Plos NTD | -6.212 | 38.381672 | 0 | Figure 3b |
| Salim; N. Knopp; S. Lweno; O. Abdul; U. Mohamed; A. Schindler; T. Rothen; J. Masimba; J. Kwaba; D. Mohammed; A. S. Althaus; F. Abdulla; S. Tanner; M. Daubenberger; C. Genton; B. | 2015 | Distribution and risk factors for Plasmodium and helminth co-infections: a cross-sectional survey among children in Bagamoyo district; coastal region of Tanzania | Plos NTD | -6.21027 | 38.37241 | 0 | Figure 3b |
| Salim; N. Knopp; S. Lweno; O. Abdul; U. Mohamed; A. Schindler; T. Rothen; J. Masimba; J. Kwaba; D. Mohammed; A. S. Althaus; F. Abdulla; S. Tanner; M. Daubenberger; C. Genton; B. | 2015 | Distribution and risk factors for Plasmodium and helminth co-infections: a cross-sectional survey among children in Bagamoyo district; coastal region of Tanzania | Plos NTD | -6.2071 | 38.370121 | 0 | Figure 3b |
| Salim; N. Knopp; S. Lweno; O. Abdul; U. Mohamed; A. Schindler; T. Rothen; J. Masimba; J. Kwaba; D. Mohammed; A. S. Althaus; F. Abdulla; S. Tanner; M. Daubenberger; C. Genton; B. | 2015 | Distribution and risk factors for Plasmodium and helminth co-infections: a cross-sectional survey among children in Bagamoyo district; coastal region of Tanzania | Plos NTD | -6.23269 | 38.36586 | 0 | Figure 3b |
| Salim; N. Knopp; S. Lweno; O. Abdul; U. Mohamed; A. Schindler; T. Rothen; J. Masimba; J. Kwaba; D. Mohammed; A. S. Althaus; F. Abdulla; S. Tanner; M. Daubenberger; C. Genton; B. | 2015 | Distribution and risk factors for Plasmodium and helminth co-infections: a cross-sectional survey among children in Bagamoyo district; coastal region of Tanzania | Plos NTD | -6.27611 | 38.36322 | 0 | Figure 3b |
| Salim; N. Knopp; S. Lweno; O. Abdul; U. Mohamed; A. Schindler; T. Rothen; J. Masimba; J. Kwaba; D. Mohammed; A. S. Althaus; F. Abdulla; S. Tanner; M. Daubenberger; C. Genton; B. | 2015 | Distribution and risk factors for Plasmodium and helminth co-infections: a cross-sectional survey among children in Bagamoyo district; coastal region of Tanzania | Plos NTD | -6.212 | 38.301109 | 0 | Figure 3b |
| Salim; N. Knopp; S. Lweno; O. Abdul; U. Mohamed; A. Schindler; T. Rothen; J. Masimba; J. Kwaba; D. Mohammed; A. S. Althaus; F. Abdulla; S. Tanner; M. Daubenberger; C. Genton; B. | 2015 | Distribution and risk factors for Plasmodium and helminth co-infections: a cross-sectional survey among children in Bagamoyo district; coastal region of Tanzania | Plos NTD | -6.2019 | 38.236469 | 0 | Figure 3b |
| Salim; N. Knopp; S. Lweno; O. Abdul; U. Mohamed; A. Schindler; T. Rothen; J. Masimba; J. Kwaba; D. Mohammed; A. S. Althaus; F. Abdulla; S. Tanner; M. Daubenberger; C. Genton; B. | 2015 | Distribution and risk factors for Plasmodium and helminth co-infections: a cross-sectional survey among children in Bagamoyo district; coastal region of Tanzania | Plos NTD | 38.35128 | -6.25671 | 3.448 | Final validation of the model |
| Salim; N. Knopp; S. Lweno; O. Abdul; U. Mohamed; A. Schindler; T. Rothen; J. Masimba; J. Kwaba; D. Mohammed; A. S. Althaus; F. Abdulla; S. Tanner; M. Daubenberger; C. Genton; B. | 2015 | Distribution and risk factors for Plasmodium and helminth co-infections: a cross-sectional survey among children in Bagamoyo district; coastal region of Tanzania | Plos NTD | 38.229809 | -6.20037 | 4 | Final validation of the model |
| Salim; N. Knopp; S. Lweno; O. Abdul; U. Mohamed; A. Schindler; T. Rothen; J. Masimba; J. Kwaba; D. Mohammed; A. S. Althaus; F. Abdulla; S. Tanner; M. Daubenberger; C. Genton; B. | 2015 | Distribution and risk factors for Plasmodium and helminth co-infections: a cross-sectional survey among children in Bagamoyo district; coastal region of Tanzania | Plos NTD | 38.352692 | -6.22357 | 5.66 | Final validation of the model |
| Salim; N. Knopp; S. Lweno; O. Abdul; U. Mohamed; A. Schindler; T. Rothen; J. Masimba; J. Kwaba; D. Mohammed; A. S. Althaus; F. Abdulla; S. Tanner; M. Daubenberger; C. Genton; B. | 2015 | Distribution and risk factors for Plasmodium and helminth co-infections: a cross-sectional survey among children in Bagamoyo district; coastal region of Tanzania | Plos NTD | 38.352921 | -6.22579 | 6 | Final validation of the model |
| Salim; N. Knopp; S. Lweno; O. Abdul; U. Mohamed; A. Schindler; T. Rothen; J. Masimba; J. Kwaba; D. Mohammed; A. S. Althaus; F. Abdulla; S. Tanner; M. Daubenberger; C. Genton; B. | 2015 | Distribution and risk factors for Plasmodium and helminth co-infections: a cross-sectional survey among children in Bagamoyo district; coastal region of Tanzania | Plos NTD | 38.484638 | -6.16894 | 7.143 | Final validation of the model |
| Salim; N. Knopp; S. Lweno; O. Abdul; U. Mohamed; A. Schindler; T. Rothen; J. Masimba; J. Kwaba; D. Mohammed; A. S. Althaus; F. Abdulla; S. Tanner; M. Daubenberger; C. Genton; B. | 2015 | Distribution and risk factors for Plasmodium and helminth co-infections: a cross-sectional survey among children in Bagamoyo district; coastal region of Tanzania | Plos NTD | 38.33535 | -6.22613 | 7.317 | Final validation of the model |
| Salim; N. Knopp; S. Lweno; O. Abdul; U. Mohamed; A. Schindler; T. Rothen; J. Masimba; J. Kwaba; D. Mohammed; A. S. Althaus; F. Abdulla; S. Tanner; M. Daubenberger; C. Genton; B. | 2015 | Distribution and risk factors for Plasmodium and helminth co-infections: a cross-sectional survey among children in Bagamoyo district; coastal region of Tanzania | Plos NTD | 38.37035 | -6.2345 | 7.692 | Final validation of the model |
| Salim; N. Knopp; S. Lweno; O. Abdul; U. Mohamed; A. Schindler; T. Rothen; J. Masimba; J. Kwaba; D. Mohammed; A. S. Althaus; F. Abdulla; S. Tanner; M. Daubenberger; C. Genton; B. | 2015 | Distribution and risk factors for Plasmodium and helminth co-infections: a cross-sectional survey among children in Bagamoyo district; coastal region of Tanzania | Plos NTD | 38.35495 | -6.22584 | 7.843 | Final validation of the model |
| Salim; N. Knopp; S. Lweno; O. Abdul; U. Mohamed; A. Schindler; T. Rothen; J. Masimba; J. Kwaba; D. Mohammed; A. S. Althaus; F. Abdulla; S. Tanner; M. Daubenberger; C. Genton; B. | 2015 | Distribution and risk factors for Plasmodium and helminth co-infections: a cross-sectional survey among children in Bagamoyo district; coastal region of Tanzania | Plos NTD | 38.36586 | -6.23269 | 8 | Final validation of the model |
| Salim; N. Knopp; S. Lweno; O. Abdul; U. Mohamed; A. Schindler; T. Rothen; J. Masimba; J. Kwaba; D. Mohammed; A. S. Althaus; F. Abdulla; S. Tanner; M. Daubenberger; C. Genton; B. | 2015 | Distribution and risk factors for Plasmodium and helminth co-infections: a cross-sectional survey among children in Bagamoyo district; coastal region of Tanzania | Plos NTD | 38.303452 | -6.2139 | 8.333 | Final validation of the model |
| Salim; N. Knopp; S. Lweno; O. Abdul; U. Mohamed; A. Schindler; T. Rothen; J. Masimba; J. Kwaba; D. Mohammed; A. S. Althaus; F. Abdulla; S. Tanner; M. Daubenberger; C. Genton; B. | 2015 | Distribution and risk factors for Plasmodium and helminth co-infections: a cross-sectional survey among children in Bagamoyo district; coastal region of Tanzania | Plos NTD | 38.231049 | -6.18825 | 8.333 | Final validation of the model |
| Salim; N. Knopp; S. Lweno; O. Abdul; U. Mohamed; A. Schindler; T. Rothen; J. Masimba; J. Kwaba; D. Mohammed; A. S. Althaus; F. Abdulla; S. Tanner; M. Daubenberger; C. Genton; B. | 2015 | Distribution and risk factors for Plasmodium and helminth co-infections: a cross-sectional survey among children in Bagamoyo district; coastal region of Tanzania | Plos NTD | 38.51516 | -6.12059 | 8.333 | Final validation of the model |
| Salim; N. Knopp; S. Lweno; O. Abdul; U. Mohamed; A. Schindler; T. Rothen; J. Masimba; J. Kwaba; D. Mohammed; A. S. Althaus; F. Abdulla; S. Tanner; M. Daubenberger; C. Genton; B. | 2015 | Distribution and risk factors for Plasmodium and helminth co-infections: a cross-sectional survey among children in Bagamoyo district; coastal region of Tanzania | Plos NTD | 38.483269 | -6.2515 | 9.677 | Final validation of the model |
| Salim; N. Knopp; S. Lweno; O. Abdul; U. Mohamed; A. Schindler; T. Rothen; J. Masimba; J. Kwaba; D. Mohammed; A. S. Althaus; F. Abdulla; S. Tanner; M. Daubenberger; C. Genton; B. | 2015 | Distribution and risk factors for Plasmodium and helminth co-infections: a cross-sectional survey among children in Bagamoyo district; coastal region of Tanzania | Plos NTD | 38.332272 | -6.25325 | 10 | Final validation of the model |
| Salim; N. Knopp; S. Lweno; O. Abdul; U. Mohamed; A. Schindler; T. Rothen; J. Masimba; J. Kwaba; D. Mohammed; A. S. Althaus; F. Abdulla; S. Tanner; M. Daubenberger; C. Genton; B. | 2015 | Distribution and risk factors for Plasmodium and helminth co-infections: a cross-sectional survey among children in Bagamoyo district; coastal region of Tanzania | Plos NTD | 38.344269 | -6.22503 | 10.204 | Final validation of the model |
| Salim; N. Knopp; S. Lweno; O. Abdul; U. Mohamed; A. Schindler; T. Rothen; J. Masimba; J. Kwaba; D. Mohammed; A. S. Althaus; F. Abdulla; S. Tanner; M. Daubenberger; C. Genton; B. | 2015 | Distribution and risk factors for Plasmodium and helminth co-infections: a cross-sectional survey among children in Bagamoyo district; coastal region of Tanzania | Plos NTD | 38.334579 | -6.23974 | 10.417 | Final validation of the model |
| Salim; N. Knopp; S. Lweno; O. Abdul; U. Mohamed; A. Schindler; T. Rothen; J. Masimba; J. Kwaba; D. Mohammed; A. S. Althaus; F. Abdulla; S. Tanner; M. Daubenberger; C. Genton; B. | 2015 | Distribution and risk factors for Plasmodium and helminth co-infections: a cross-sectional survey among children in Bagamoyo district; coastal region of Tanzania | Plos NTD | 38.344849 | -6.2332 | 10.526 | Final validation of the model |
| Salim; N. Knopp; S. Lweno; O. Abdul; U. Mohamed; A. Schindler; T. Rothen; J. Masimba; J. Kwaba; D. Mohammed; A. S. Althaus; F. Abdulla; S. Tanner; M. Daubenberger; C. Genton; B. | 2015 | Distribution and risk factors for Plasmodium and helminth co-infections: a cross-sectional survey among children in Bagamoyo district; coastal region of Tanzania | Plos NTD | 38.33535 | -6.22613 | 11.111 | Final validation of the model |
| Salim; N. Knopp; S. Lweno; O. Abdul; U. Mohamed; A. Schindler; T. Rothen; J. Masimba; J. Kwaba; D. Mohammed; A. S. Althaus; F. Abdulla; S. Tanner; M. Daubenberger; C. Genton; B. | 2015 | Distribution and risk factors for Plasmodium and helminth co-infections: a cross-sectional survey among children in Bagamoyo district; coastal region of Tanzania | Plos NTD | 38.350761 | -6.22325 | 11.538 | Final validation of the model |
| Salim; N. Knopp; S. Lweno; O. Abdul; U. Mohamed; A. Schindler; T. Rothen; J. Masimba; J. Kwaba; D. Mohammed; A. S. Althaus; F. Abdulla; S. Tanner; M. Daubenberger; C. Genton; B. | 2015 | Distribution and risk factors for Plasmodium and helminth co-infections: a cross-sectional survey among children in Bagamoyo district; coastal region of Tanzania | Plos NTD | 38.23148 | -6.19949 | 12.5 | Final validation of the model |
| Salim; N. Knopp; S. Lweno; O. Abdul; U. Mohamed; A. Schindler; T. Rothen; J. Masimba; J. Kwaba; D. Mohammed; A. S. Althaus; F. Abdulla; S. Tanner; M. Daubenberger; C. Genton; B. | 2015 | Distribution and risk factors for Plasmodium and helminth co-infections: a cross-sectional survey among children in Bagamoyo district; coastal region of Tanzania | Plos NTD | 38.449139 | -6.1389 | 13.333 | Final validation of the model |
| Salim; N. Knopp; S. Lweno; O. Abdul; U. Mohamed; A. Schindler; T. Rothen; J. Masimba; J. Kwaba; D. Mohammed; A. S. Althaus; F. Abdulla; S. Tanner; M. Daubenberger; C. Genton; B. | 2015 | Distribution and risk factors for Plasmodium and helminth co-infections: a cross-sectional survey among children in Bagamoyo district; coastal region of Tanzania | Plos NTD | 38.35294 | -6.22103 | 14.286 | Final validation of the model |
| Salim; N. Knopp; S. Lweno; O. Abdul; U. Mohamed; A. Schindler; T. Rothen; J. Masimba; J. Kwaba; D. Mohammed; A. S. Althaus; F. Abdulla; S. Tanner; M. Daubenberger; C. Genton; B. | 2015 | Distribution and risk factors for Plasmodium and helminth co-infections: a cross-sectional survey among children in Bagamoyo district; coastal region of Tanzania | Plos NTD | 38.343948 | -6.2459 | 16.667 | Final validation of the model |
| Salim; N. Knopp; S. Lweno; O. Abdul; U. Mohamed; A. Schindler; T. Rothen; J. Masimba; J. Kwaba; D. Mohammed; A. S. Althaus; F. Abdulla; S. Tanner; M. Daubenberger; C. Genton; B. | 2015 | Distribution and risk factors for Plasmodium and helminth co-infections: a cross-sectional survey among children in Bagamoyo district; coastal region of Tanzania | Plos NTD | 38.337742 | -6.22579 | 16.667 | Final validation of the model |
| Salim; N. Knopp; S. Lweno; O. Abdul; U. Mohamed; A. Schindler; T. Rothen; J. Masimba; J. Kwaba; D. Mohammed; A. S. Althaus; F. Abdulla; S. Tanner; M. Daubenberger; C. Genton; B. | 2015 | Distribution and risk factors for Plasmodium and helminth co-infections: a cross-sectional survey among children in Bagamoyo district; coastal region of Tanzania | Plos NTD | 38.375542 | -6.22066 | 16.667 | Final validation of the model |
| Salim; N. Knopp; S. Lweno; O. Abdul; U. Mohamed; A. Schindler; T. Rothen; J. Masimba; J. Kwaba; D. Mohammed; A. S. Althaus; F. Abdulla; S. Tanner; M. Daubenberger; C. Genton; B. | 2015 | Distribution and risk factors for Plasmodium and helminth co-infections: a cross-sectional survey among children in Bagamoyo district; coastal region of Tanzania | Plos NTD | 38.337742 | -6.22579 | 20 | Final validation of the model |
| Salim; N. Knopp; S. Lweno; O. Abdul; U. Mohamed; A. Schindler; T. Rothen; J. Masimba; J. Kwaba; D. Mohammed; A. S. Althaus; F. Abdulla; S. Tanner; M. Daubenberger; C. Genton; B. | 2015 | Distribution and risk factors for Plasmodium and helminth co-infections: a cross-sectional survey among children in Bagamoyo district; coastal region of Tanzania | Plos NTD | 38.346191 | -6.2244 | 21.429 | Final validation of the model |
| Salim; N. Knopp; S. Lweno; O. Abdul; U. Mohamed; A. Schindler; T. Rothen; J. Masimba; J. Kwaba; D. Mohammed; A. S. Althaus; F. Abdulla; S. Tanner; M. Daubenberger; C. Genton; B. | 2015 | Distribution and risk factors for Plasmodium and helminth co-infections: a cross-sectional survey among children in Bagamoyo district; coastal region of Tanzania | Plos NTD | 38.340321 | -6.22576 | 25 | Final validation of the model |
| Salim; N. Knopp; S. Lweno; O. Abdul; U. Mohamed; A. Schindler; T. Rothen; J. Masimba; J. Kwaba; D. Mohammed; A. S. Althaus; F. Abdulla; S. Tanner; M. Daubenberger; C. Genton; B. | 2015 | Distribution and risk factors for Plasmodium and helminth co-infections: a cross-sectional survey among children in Bagamoyo district; coastal region of Tanzania | Plos NTD | 38.396278 | -6.11882 | 25 | Final validation of the model |
| Salim; N. Knopp; S. Lweno; O. Abdul; U. Mohamed; A. Schindler; T. Rothen; J. Masimba; J. Kwaba; D. Mohammed; A. S. Althaus; F. Abdulla; S. Tanner; M. Daubenberger; C. Genton; B. | 2015 | Distribution and risk factors for Plasmodium and helminth co-infections: a cross-sectional survey among children in Bagamoyo district; coastal region of Tanzania | Plos NTD | 38.332272 | -6.25325 | 27.273 | Final validation of the model |
| Salim; N. Knopp; S. Lweno; O. Abdul; U. Mohamed; A. Schindler; T. Rothen; J. Masimba; J. Kwaba; D. Mohammed; A. S. Althaus; F. Abdulla; S. Tanner; M. Daubenberger; C. Genton; B. | 2015 | Distribution and risk factors for Plasmodium and helminth co-infections: a cross-sectional survey among children in Bagamoyo district; coastal region of Tanzania | Plos NTD | 38.356731 | -6.24767 | 28.889 | Final validation of the model |
| Salim; N. Knopp; S. Lweno; O. Abdul; U. Mohamed; A. Schindler; T. Rothen; J. Masimba; J. Kwaba; D. Mohammed; A. S. Althaus; F. Abdulla; S. Tanner; M. Daubenberger; C. Genton; B. | 2015 | Distribution and risk factors for Plasmodium and helminth co-infections: a cross-sectional survey among children in Bagamoyo district; coastal region of Tanzania | Plos NTD | 38.378078 | -6.24283 | 33.333 | Final validation of the model |
| Salim; N. Knopp; S. Lweno; O. Abdul; U. Mohamed; A. Schindler; T. Rothen; J. Masimba; J. Kwaba; D. Mohammed; A. S. Althaus; F. Abdulla; S. Tanner; M. Daubenberger; C. Genton; B. | 2015 | Distribution and risk factors for Plasmodium and helminth co-infections: a cross-sectional survey among children in Bagamoyo district; coastal region of Tanzania | Plos NTD | 38.264462 | -6.21001 | 33.333 | Final validation of the model |
| Salim; N. Knopp; S. Lweno; O. Abdul; U. Mohamed; A. Schindler; T. Rothen; J. Masimba; J. Kwaba; D. Mohammed; A. S. Althaus; F. Abdulla; S. Tanner; M. Daubenberger; C. Genton; B. | 2015 | Distribution and risk factors for Plasmodium and helminth co-infections: a cross-sectional survey among children in Bagamoyo district; coastal region of Tanzania | Plos NTD | 38.344849 | -6.2332 | 40 | Final validation of the model |
| Salim; N. Knopp; S. Lweno; O. Abdul; U. Mohamed; A. Schindler; T. Rothen; J. Masimba; J. Kwaba; D. Mohammed; A. S. Althaus; F. Abdulla; S. Tanner; M. Daubenberger; C. Genton; B. | 2015 | Distribution and risk factors for Plasmodium and helminth co-infections: a cross-sectional survey among children in Bagamoyo district; coastal region of Tanzania | Plos NTD | 38.301109 | -6.212 | 50 | Final validation of the model |
| Salim; N. Knopp; S. Lweno; O. Abdul; U. Mohamed; A. Schindler; T. Rothen; J. Masimba; J. Kwaba; D. Mohammed; A. S. Althaus; F. Abdulla; S. Tanner; M. Daubenberger; C. Genton; B. | 2015 | Distribution and risk factors for Plasmodium and helminth co-infections: a cross-sectional survey among children in Bagamoyo district; coastal region of Tanzania | Plos NTD | 38.346191 | -6.2244 | 100 | Final validation of the model |
| Salim; N. Knopp; S. Lweno; O. Abdul; U. Mohamed; A. Schindler; T. Rothen; J. Masimba; J. Kwaba; D. Mohammed; A. S. Althaus; F. Abdulla; S. Tanner; M. Daubenberger; C. Genton; B. | 2015 | Distribution and risk factors for Plasmodium and helminth co-infections: a cross-sectional survey among children in Bagamoyo district; coastal region of Tanzania | Plos NTD | 38.23101 | -6.16804 | 100 | Final validation of the model |
| Siwila; J. Phiri; I. G. Enemark; H. L. Nchito; M. Olsen; A. | 2010 | Intestinal helminths and protozoa in children in pre-schools in Kafue district; Zambia | Transactions of the Royal Society of Tropical Medicine and Hygiene | 28.44125 | -15.61994 | 2.128 | Final validation of the model |
| Siwila; J. Phiri; I. G. Enemark; H. L. Nchito; M. Olsen; A. | 2010 | Intestinal helminths and protozoa in children in pre-schools in Kafue district; Zambia | Transactions of the Royal Society of Tropical Medicine and Hygiene | 28.44125 | -15.61994 | 2.174 | Final validation of the model |
| Siwila; J. Phiri; I. G. Enemark; H. L. Nchito; M. Olsen; A. | 2010 | Intestinal helminths and protozoa in children in pre-schools in Kafue district; Zambia | Transactions of the Royal Society of Tropical Medicine and Hygiene | 28.44125 | -15.61994 | 4.444 | Final validation of the model |
| Siwila; J. Phiri; I. G. Enemark; H. L. Nchito; M. Olsen; A. | 2010 | Intestinal helminths and protozoa in children in pre-schools in Kafue district; Zambia | Transactions of the Royal Society of Tropical Medicine and Hygiene | 28.44125 | -15.61994 | 5.128 | Final validation of the model |
| Siwila; J. Phiri; I. G. Enemark; H. L. Nchito; M. Olsen; A. | 2010 | Intestinal helminths and protozoa in children in pre-schools in Kafue district; Zambia | Transactions of the Royal Society of Tropical Medicine and Hygiene | 28.44125 | -15.61994 | 12 | Final validation of the model |
| Siwila; J. Phiri; I. G. Enemark; H. L. Nchito; M. Olsen; A. | 2010 | Intestinal helminths and protozoa in children in pre-schools in Kafue district; Zambia | Transactions of the Royal Society of Tropical Medicine and Hygiene | 28.44125 | -15.61994 | 12 | Final validation of the model |
| Siwila; J. Phiri; I. G. Enemark; H. L. Nchito; M. Olsen; A. | 2010 | Intestinal helminths and protozoa in children in pre-schools in Kafue district; Zambia | Transactions of the Royal Society of Tropical Medicine and Hygiene | 28.44125 | -15.61994 | 12.5 | Final validation of the model |
| Siwila; J. Phiri; I. G. Enemark; H. L. Nchito; M. Olsen; A. | 2010 | Intestinal helminths and protozoa in children in pre-schools in Kafue district; Zambia | Transactions of the Royal Society of Tropical Medicine and Hygiene | 28.44125 | -15.61994 | 13.043 | Final validation of the model |
