# Supplementary material for: Mapping the global distribution of Strongyloides stercoralis and hookworms by ecological niche modeling
Source: Parasit Vectors. 2022 Jun 8;15:197. doi: 10.1186/s13071-022-05284-w (PMC9178904; doi:10.1186/s13071-022-05284-w)
Supplement: Supplementary file 3 — Additional file 3: Table S3: Nested anova of mobility area (M) of S. stercoralis and hookworms. [file 13071_2022_5284_MOESM3_ESM.docx]

# Additional file 3: Table S3: Nested anova of mobility area (M) of *S. stercoralis* and hookworms

| ***S. stercoralis*** |  |  |  |
| --- | --- | --- | --- |
| **Continent** | **M distance (km)** | **Nested anova** | **Tukey** |
| America | 1877.4 | p<0.01 | a |
| Africa | 1085.5 |  | b |
| Asia | 976 |  | b |
| **Subcontinent** | | | |
| South America | 2516.0 |  | a |
| Central America | 1297.7 |  | b |
| West africa | 1103.1 |  | b |
| East Africa | 1025.8 |  | b |
| West Asia | 891.1 |  | b |
| East Asia | 830.8 |  | b |
| **Size of M used for ENM** | | | |
|  | **100%** | **50%** | **25%** |
| South America | 2516 | 1258 | 629 |
| Central America, Asia and Africa | 1297 | 649 | 324 |
|  |  |  |  |
| **Hookworm** |  |  |  |
| **Continent** | **M distance (km)** | **Nested anova** | **Tukey** |
| America | 1963,7 | p<0.01 | a |
| Africa | 1206,7 |  | b |
| Asia | 1148,6 |  | b |
| Subcontinent | | |  |
| South America | 2365,7 |  | a |
| Central America | 1341,4 |  | b |
| West africa | 1177,5 |  | b |
| East Africa | 1134,2 |  | b |
| West Asia | 1094,5 |  | b |
| East Asia | 1025,8 |  | b |
| **Size of M used for ENM** | | | |
|  | **100%** | **50%** | **25%** |
| South America | 2366 | 1183 | 591,5 |
| Central America, Asia and Africa | 1341 | 670,5 | 335,3 |
