# Supplementary material for: Mapping the global distribution of Strongyloides stercoralis and hookworms by ecological niche modeling
Source: Parasit Vectors. 2022 Jun 8;15:197. doi: 10.1186/s13071-022-05284-w (PMC9178904; doi:10.1186/s13071-022-05284-w)
Supplement: Supplementary file 5 — Additional file 5: Table S4: Final niche models for S. stercoralis. [file 13071_2022_5284_MOESM5_ESM.docx]

# Additional file 5: Table S4: Final niche models for *S. stercoralis*

| **Model (M size; feature class)** | **Mean AUC ratio** | **Omission rate at 5%** | **AIC** | **Delta AIC** | **W AIC** | **Number of variables** | **Transfer of suitability between areas** | | | **Final evaluation** |
| --- | --- | --- | --- | --- | --- | --- | --- | --- | --- | --- |
|  |  |  |  |  |  |  | **AUC extrapolation model** | **AUC clamping extrapolation model** | **AUC no extrapolation model** | **Mean AUC ratio** |
| S.A (100%; qt) | 1.35 | 0.043 | 2858.965 | 0 | 0.499 | 6 | 0.914 | 0.907 | 0.92 | 1.32 |
| S.B (100%; lpt) | 1.349 | 0.043 | 2860.858 | 1.893 | 0.193 | 6 | 0.916 | 0.913 | 0.916 | 1.25 |
| S.C (50%; qt) | 1.286 | 0.043 | 2802.524 | 0 | 0.417 | 6 | 0.861 | 0.876 | 0.88 | 1.25 |
| S.D (50%, t) | 1.257 | 0.043 | 2803.306 | 0.781 | 0.282 | 6 | 0.894 | 0.887 | 0.889 | 1.19 |
| S.E (50%, qt) | 1.305 | 0.043 | 2804.368 | 1.844 | 0.166 | 8 | 0.878 | 0.874 | 0.88 | 1.18 |
| S.G (25%, t) | 1.247 | 0.043 | 2684.428 | 0 | 0.773 | 6 | 0.85 | 0.855 | 0.859 | 1.32 |
