# Supplementary material for: Mapping the global distribution of Strongyloides stercoralis and hookworms by ecological niche modeling
Source: Parasit Vectors. 2022 Jun 8;15:197. doi: 10.1186/s13071-022-05284-w (PMC9178904; doi:10.1186/s13071-022-05284-w)
Supplement: Supplementary file 6 — Additional file 6: Table S5: Final niche models for hookworms. [file 13071_2022_5284_MOESM6_ESM.docx]

# Additional file 6: Table S5: Final niche models for hookworms

| **Model (M size; feature class)** | **Mean AUC ratio** | **Omission rate at 5%** | **AIC** | **Delta AIC** | **W AIC** | **Number of variables** | **Transfer of suitability between areas** | | | **Final evaluation** |
| --- | --- | --- | --- | --- | --- | --- | --- | --- | --- | --- |
|  |  |  |  |  |  |  | **AUC extrapolation model** | **AUC clamping extrapolation model** | **AUC no extrapolation model** | **Mean AUC ratio** |
| H.A (100%; lph) | 1.369 | 0.043 | 4287.089 | 0 | 0.946 | 13 | 0.895 | 0.884 | 0.888 | 1.386 |
| H.B (100%; lpth) | 1.377 | 0.043 | 4287.482 | 0.393 | 0.437 | 13 | 0.877 | 0.887 | 0.893 | 1.355 |
| H.C (50%; qpt) | 1.248 | 0.043 | 4137.248 | 0 | 0.982 | 9 | 0.86 | 0.887 | 0.871 | 1.589 |
