# Supplementary material for: Mapping the global distribution of Strongyloides stercoralis and hookworms by ecological niche modeling
Source: Parasit Vectors. 2022 Jun 8;15:197. doi: 10.1186/s13071-022-05284-w (PMC9178904; doi:10.1186/s13071-022-05284-w)

# Additional file 9: Figure S1: Scatter plot graphs of the prevalence versus the probability of presence

1. **S. stercoralis**
2. **Hookworms**


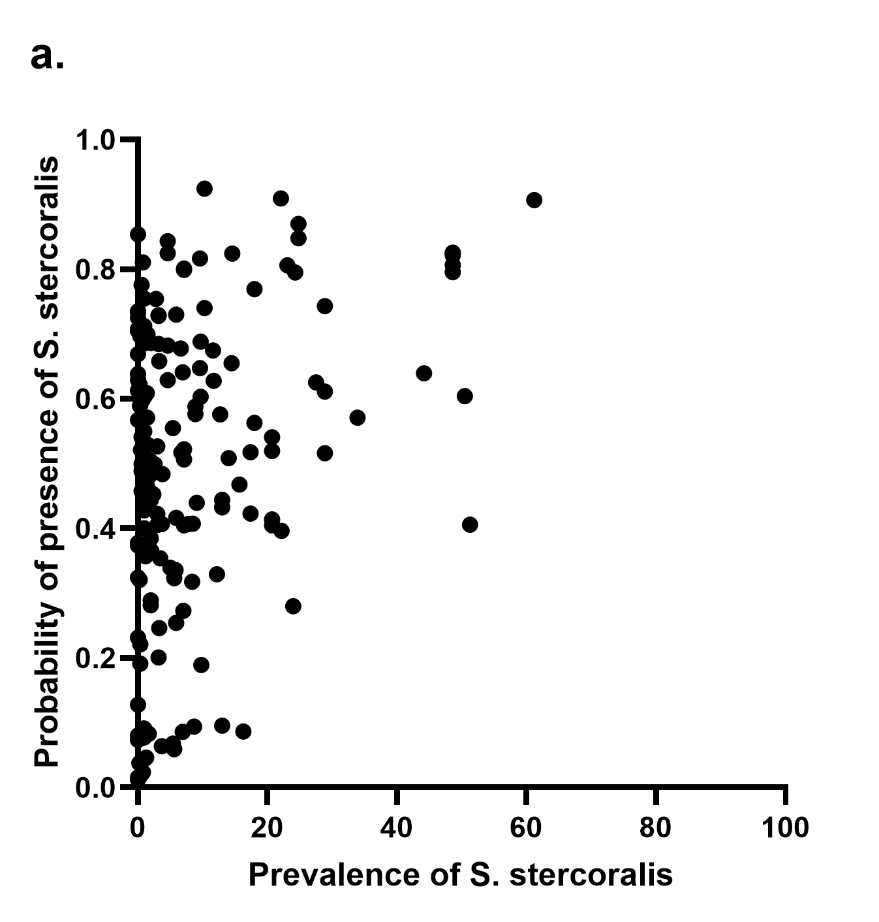


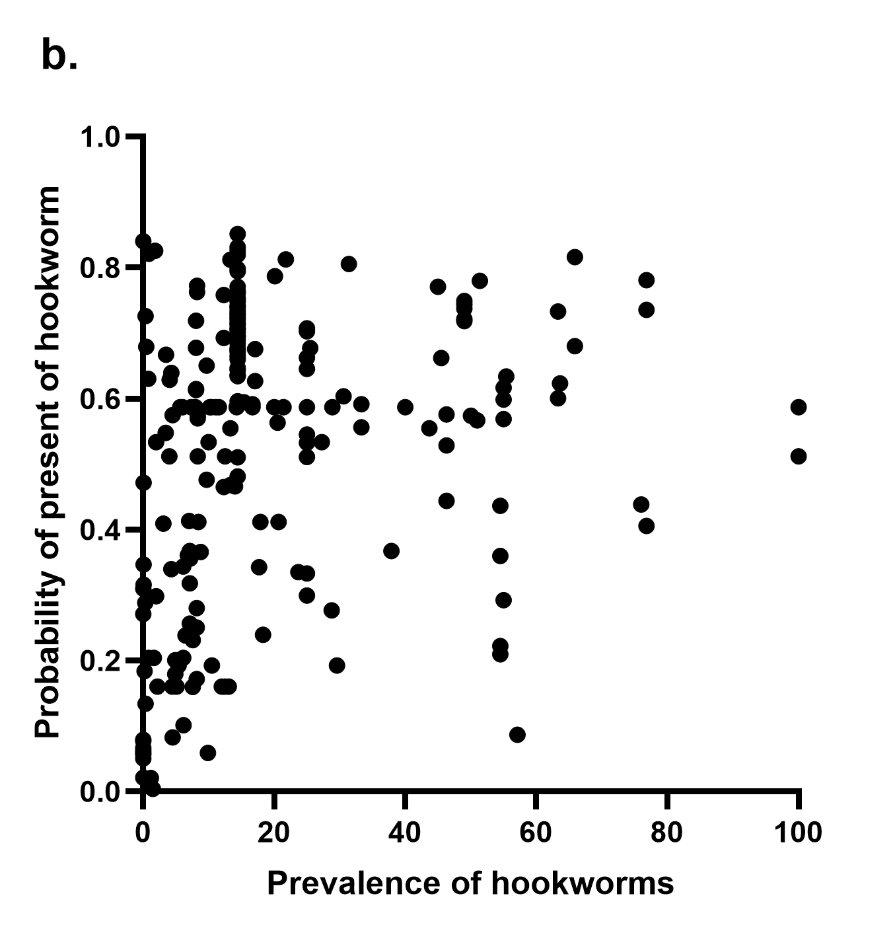

Supplement: Supplementary file 9 — Additional file 9: Figure S1: Scatter plot graphs of the prevalence versus the probability of presence. [file 13071_2022_5284_MOESM9_ESM.docx]
