# Supplementary material for: Mapping the global distribution of Strongyloides stercoralis and hookworms by ecological niche modeling
Source: Parasit Vectors. 2022 Jun 8;15:197. doi: 10.1186/s13071-022-05284-w (PMC9178904; doi:10.1186/s13071-022-05284-w)
Supplement: Supplementary file 10 — Additional file 10: Table S6: Risk population of S. stercoralis and hookworms. [file 13071_2022_5284_MOESM10_ESM.docx]

# Additional file 10: Table S6: Risk population of *S. stercoralis* and hookworm

St: *S. stercoralis*. HKW: hookworm.

| **Continent** | **Country** | **Country Population** | **Low risk ST** | **High risk ST** | **Total risk ST** | **ST % risk Population** | **Low risk HKW** | **High risk HKW** | **Total risk HKW** | **HKW % risk Population** | |
| --- | --- | --- | --- | --- | --- | --- | --- | --- | --- | --- | --- |
| Asia | India | 1525704000 | 302142700 | 165507400 | 467650100 | 30.7 | 624915700 | 644776800 | 1269692500 | 83.2 | |
| Asia | China | 1551020000 | 298324300 | 139983000 | 438307300 | 28.3 | 357562200 | 12475960 | 370038160 | 23.9 | |
| Asia | Bangladesh | 174805500 | 17704140 | 148211800 | 165915940 | 94.9 | 92233450 | 82287390 | 174520840 | 99.8 | |
| Asia | Indonesia | 292994400 | 55381560 | 119335800 | 174717360 | 59.6 | 128262700 | 40321320 | 168584020 | 57.5 | |
| Asia | Vietnam | 104696400 | 43547960 | 46108360 | 89656320 | 85.6 | 49255370 | 51241810 | 100497180 | 96.0 | |
| Asia | Philippines | 107016400 | 16340730 | 72892130 | 89232860 | 83.4 | 38344580 | 60912490 | 99257070 | 92.7 | |
| Asia | Thailand | 69214870 | 14140930 | 51142890 | 65283820 | 94.3 | 14313050 | 54574500 | 68887550 | 99.5 | |
| Asia | Myanmar | 52648290 | 14634440 | 24251820 | 38886260 | 73.9 | 21555130 | 29246600 | 50801730 | 96.5 | |
| Asia | Malaysia | 35891510 | 8207470 | 20462660 | 28670130 | 79.9 | 14228850 | 17892050 | 32120900 | 89.5 | |
| Asia | Nepal | 43764220 | 4894018 | 3472623 | 8366641 | 19.1 | 29030390 | 359002 | 29389392 | 67.2 | |
| Asia | Taiwan | 27261130 | 4204478 | 21246150 | 25450628 | 93.4 | 15347920 | 10260100 | 25608020 | 93.9 | |
| Asia | Sri Lanka | 22023120 | 2336966 | 16675390 | 19012356 | 86.3 | 5011023 | 16321470 | 21332493 | 96.9 | |
| Asia | Cambodia | 20753800 | 581234 | 19863400 | 20444634 | 98.5 | 923075 | 19780670 | 20703745 | 99.8 | |
| Asia | Japan | 116251400 | 35798290 | 33442740 | 69241030 | 59.6 | 13065890 | 290119 | 13356009 | 11.5 | |
| Asia | Pakistan | 249814200 | 6714416 | 1554487 | 8268903 | 3.3 | 8335418 | 60316 | 8395734 | 3.4 | |
| Asia | Laos | 8234621 | 485617 | 7068277 | 7553894 | 91.7 | 3447834 | 4323710 | 7771544 | 94.4 | |
| Asia | Lebanon | 10297930 | 499478 | 189449 | 688927 | 6.7 | 6217280 | 49959 | 6267239 | 60.9 | |
| Asia | Singapore | 3724111 | 929252 | 2268507 | 3197759 | 85.9 | 758591 | 2965520 | 3724111 | 100.0 | |
| Asia | Iran | 85645170 | 771025 | 1010543 | 1781568 | 2.1 | 1804392 | 0 | 1804392 | 2.1 | |
| Asia | Syria | 29204210 | 534980 | 204600 | 739580 | 2.5 | 1268804 | 3946 | 1272750 | 4.4 | |
| Asia | East Timor | 1417368 | 217519 | 964870 | 1182389 | 83.4 | 689440 | 508860 | 1198299 | 84.5 | |
| Asia | Brunei | 415170 | 51401 | 275520 | 326921 | 78.7 | 89977 | 315612 | 405589 | 97.7 | |
| Asia | Republic of Korea | 53122490 | 21475500 | 17533070 | 39008570 | 73.4 | 361465 | 0 | 361465 | 0.7 | |
| Asia | Bhutan | 908324 | 233952 | 56075 | 290027 | 31.9 | 177440 | 0 | 177440 | 19.5 | |
| Asia | Tajikistan | 9807599 | 47 | 0 | 47 | 0.0 | 63 | 0 | 63 | 0.0 | |
| Asia | North Korea | 24701090 | 5498357 | 178531 | 5676888 | 23.0 | 0 | 0 | 0 | 0.0 | |
| Asia | Russia | 153647700 | 136257 | 0 | 136257 | 0.1 | 0 | 0 | 0 | 0.0 | |
| Asia | Azerbaijan | 10897420 | 25077 | 0 | 25077 | 0.2 | 0 | 0 | 0 | 0.0 | |
| Asia | Iraq | 47867330 | 952 | 0 | 952 | 0.0 | 0 | 0 | 0 | 0.0 | |
| Asia | Afghanistan | 33827380 | 19 | 0 | 19 | 0.0 | 0 | 0 | 0 | 0.0 | |
| Asia | Saudi Arabia | 39183190 | 0 | 0 | 0 | 0.0 | 0 | 0 | 0 | 0.0 | |
| Asia | Armenia | 3009644 | 0 | 0 | 0 | 0.0 | 0 | 0 | 0 | 0.0 | |
| Asia | Bahrain | 1179235 | 0 | 0 | 0 | 0.0 | 0 | 0 | 0 | 0.0 | |
| Asia | Qatar | 6389321 | 0 | 0 | 0 | 0.0 | 0 | 0 | 0 | 0.0 | |
| Asia | United Arab Emirates | 9773146 | 0 | 0 | 0 | 0.0 | 0 | 0 | 0 | 0.0 | |
| Asia | Israel | 9123917 | 0 | 0 | 0 | 0.0 | 0 | 0 | 0 | 0.0 | |
| Asia | Jordanian | 8145465 | 0 | 0 | 0 | 0.0 | 0 | 0 | 0 | 0.0 | |
| Asia | Kazakhstan | 19533370 | 0 | 0 | 0 | 0.0 | 0 | 0 | 0 | 0.0 | |
| Asia | Kirgizstan | 6123900 | 0 | 0 | 0 | 0.0 | 0 | 0 | 0 | 0.0 | |
| Asia | Kuwait | 3268944 | 0 | 0 | 0 | 0.0 | 0 | 0 | 0 | 0.0 | |
| Asia | Mongolia | 3459203 | 0 | 0 | 0 | 0.0 | 0 | 0 | 0 | 0.0 | |
| Asia | Oman | 3982123 | 0 | 0 | 0 | 0.0 | 0 | 0 | 0 | 0.0 | |
| Asia | Palestine | 5935187 | 0 | 0 | 0 | 0.0 | 0 | 0 | 0 | 0.0 | |
| Asia | Turkmenistan | 10728570 | 0 | 0 | 0 | 0.0 | 0 | 0 | 0 | 0.0 | |
| Asia | Uzbekistan | 36329930 | 0 | 0 | 0 | 0.0 | 0 | 0 | 0 | 0.0 | |
| Asia | Yemen | 32387550 | 0 | 0 | 0 | 0.0 | 0 | 0 | 0 | 0.0 | |
|  | **Total Asia** | **5066129848** | **855813064** | **913900091** | **1769713155** | **34.9** | **1427200031** | **1048968205** | **2476168236** | **48.9** | |
| Africa | Nigeria | 234119300 | 37748330 | 92875220 | 130623550 | 55.8 | 87618050 | 82008350 | 169626400 | 72.5 | |
| Africa | Ethiopia | 113451000 | 30060080 | 25832740 | 55892820 | 49.3 | 19713430 | 964709 | 20678139 | 18.2 | |
| Africa | Democratic Republic of Congo | 123728800 | 41440290 | 7917517 | 49357807 | 39.9 | 38628350 | 4434984 | 43063334 | 34.8 | |
| Africa | Tanzania | 57917100 | 18496130 | 9655451 | 28151581 | 48.6 | 22644170 | 7809555 | 30453725 | 52.6 | |
| Africa | Kenya | 59367620 | 18390450 | 9202365 | 27592815 | 46.5 | 7819138 | 2754353 | 10573491 | 17.8 | |
| Africa | Uganda | 46450830 | 20825890 | 2750002 | 23575892 | 50.8 | 19194460 | 49537 | 19243997 | 41.4 | |
| Africa | Ghana | 34533040 | 5550518 | 17123310 | 22673828 | 65.7 | 17174730 | 14846110 | 32020840 | 92.7 | |
| Africa | Ivory Coast | 27794330 | 4764946 | 15405090 | 20170036 | 72.6 | 10656110 | 13149260 | 23805370 | 85.6 | |
| Africa | Madagascar | 29765000 | 11715950 | 7785054 | 19501004 | 65.5 | 13222190 | 3262890 | 16485080 | 55.4 | |
| Africa | Mozambique | 32683760 | 11334010 | 6888729 | 18222739 | 55.8 | 23818480 | 3221383 | 27039863 | 82.7 | |
| Africa | Benin | 14528170 | 2479245 | 11037220 | 13516465 | 93.0 | 1575018 | 12953150 | 14528168 | 100.0 | |
| Africa | Cameroon | 31193680 | 8282197 | 4389846 | 12672043 | 40.6 | 14768670 | 5637044 | 20405714 | 65.4 | |
| Africa | Burundi | 12557510 | 7272909 | 2695950 | 9968859 | 79.4 | 3073702 | 0 | 3073702 | 24.5 | |
| Africa | Togo | 10324890 | 1769606 | 7801851 | 9571457 | 92.7 | 1195219 | 9015755 | 10210974 | 98.9 | |
| Africa | Rwanda | 15245550 | 5123589 | 3725822 | 8849411 | 58.0 | 708038 | 0 | 708038 | 4.6 | |
| Africa | Malawi | 20895480 | 7956840 | 494250 | 8451090 | 40.4 | 14024630 | 543455 | 14568085 | 69.7 | |
| Africa | Guinea | 13387340 | 5435156 | 2753312 | 8188468 | 61.2 | 5447606 | 5538328 | 10985934 | 82.1 | |
| Africa | South Sudan | 18371070 | 3964591 | 3104310 | 7068901 | 38.5 | 10960390 | 5854799 | 16815189 | 91.5 | |
| Africa | South Africa | 65642840 | 3297488 | 2014907 | 5312395 | 8.1 | 5414479 | 76011 | 5490490 | 8.4 | |
| Africa | Angola | 34867690 | 5022340 | 60460 | 5082800 | 14.6 | 0 | 69734 | 69734 | 0.2 | |
| Africa | Zambia | 20338930 | 4706599 | 209960 | 4916559 | 24.2 | 6548697 | 10570 | 6559267 | 32.2 | |
| Africa | Central African Republic | 5687078 | 3819006 | 768290 | 4587296 | 80.7 | 4234209 | 462402 | 4696611 | 82.6 | |
| Africa | Chad | 18059240 | 2785985 | 1361317 | 4147302 | 23.0 | 6632112 | 1441568 | 8073680 | 44.7 | |
| Africa | Burkina Faso | 24307930 | 389719 | 3234879 | 3624598 | 14.9 | 9577150 | 10521150 | 20098300 | 82.7 | |
| Africa | Mali | 24817060 | 815472 | 2589819 | 3405291 | 13.7 | 7338774 | 7433335 | 14772109 | 59.5 | |
| Africa | Sierra Leone | 7014469 | 2495458 | 159543 | 2655001 | 37.9 | 3712325 | 443696 | 4156021 | 59.2 | |
| Africa | Liberia | 4914009 | 1772713 | 171723 | 1944436 | 39.6 | 1880359 | 0 | 1880359 | 38.3 | |
| Africa | Congo | 4177284 | 949008 | 688942 | 1637950 | 39.2 | 1239140 | 700387 | 1939527 | 46.4 | |
| Africa | Senegal | 16639990 | 1181427 | 174800 | 1356227 | 8.2 | 2568002 | 2178769 | 4746771 | 28.5 | |
| Africa | Guinea-Bissau | 1673193 | 998630 | 52673 | 1051303 | 62.8 | 302066 | 1370939 | 1673005 | 100.0 | |
| Africa | Gabon | 3143374 | 351087 | 544583 | 895670 | 28.5 | 460481 | 746705 | 1207186 | 38.4 | |
| Africa | Reunion (Francia) | 445819 | 152538 | 250774 | 403312 | 90.5 | 343927 | 101892 | 445819 | 100.0 | |
| Africa | Mauricio | 376005 | 614 | 375391 | 376005 | 100.0 | 118336 | 257669 | 376005 | 100.0 | |
| Africa | Swazilander | 1217190 | 220158 | 84738 | 304896 | 25.0 | 221644 | 0 | 221644 | 18.2 | |
| Africa | Comoros | 488563 | 105258 | 157914 | 263171 | 53.9 | 268392 | 0 | 268392 | 54.9 | |
| Africa | Zimbabwe | 15929030 | 173731 | 6183 | 179914 | 1.1 | 274025 | 0 | 274025 | 1.7 | |
| Africa | Equatorial Guinea | 1268450 | 93495 | 0 | 93495 | 7.4 | 155797 | 0 | 155797 | 12.3 | |
| Africa | Mayotte (France) | 201475 | 35162 | 54298 | 89460 | 44.4 | 113349 | 24250 | 137599 | 68.3 | |
| Africa | Gambia | 2488197 | 48412 | 10477 | 58889 | 2.4 | 1196274 | 1287020 | 2483294 | 99.8 | |
| Africa | Sao Tome and Principe | 155071 | 3080 | 36190 | 39270 | 25.3 | 100775 | 3871 | 104646 | 67.5 | |
| Africa | Sudan | 45588780 | 16934 | 7616 | 24550 | 0.1 | 3278768 | 94298 | 3373066 | 7.4 | |
| Africa | Marruecos | 38150400 | 11389 | 0 | 11389 | 0.0 | 378990 | 0 | 378990 | 1.0 | |
| Africa | Lesotho | 1958824 | 2388 | 0 | 2388 | 0.1 | 0 | 0 | 0 | 0.0 | |
| Africa | Argelia | 46962260 | 0 | 0 | 0 | 0.0 | 0 | 0 | 0 | 0.0 | |
| Africa | Botswana | 2632876 | 0 | 0 | 0 | 0.0 | 0 | 0 | 0 | 0.0 | |
| Africa | Cape Verde | 388574 | 0 | 0 | 0 | 0.0 | 0 | 0 | 0 | 0.0 | |
| Africa | Egypt | 103374500 | 0 | 0 | 0 | 0.0 | 0 | 0 | 0 | 0.0 | |
| Africa | Eritrea | 4456958 | 0 | 0 | 0 | 0.0 | 43407 | 0 | 43407 | 1.0 | |
| Africa | Libya | 7595685 | 0 | 0 | 0 | 0.0 | 0 | 0 | 0 | 0.0 | |
| Africa | Mauritania | 4513858 | 0 | 0 | 0 | 0.0 | 3158 | 0 | 3158 | 0.1 | |
| Africa | Niger | 26187340 | 0 | 0 | 0 | 0.0 | 780366 | 1578 | 781943 | 3.0 | |
| Africa | Namibia | 2550943 | 0 | 0 | 0 | 0.0 | 0 | 0 | 0 | 0.0 | |
| Africa | Occidental Sahara | 404855 | 0 | 0 | 0 | 0.0 | 0 | 0 | 0 | 0.0 | |
| Africa | Somalia | 12158310 | 0 | 0 | 0 | 0.0 | 1142935 | 0 | 1142935 | 9.4 | |
| Africa | Tunes | 12093510 | 0 | 0 | 0 | 0.0 | 0 | 0 | 0 | 0.0 | |
| Africa | Djibouti | 1213408 | 0 | 0 | 0 | 0.0 | 488144 | 0 | 488144 | 40.2 | |
|  | **Total** | **1460398437** | **272058817** | **244453513** | **516512331** | **35.4** | **371058460** | **199269506** | **570327966** | **39.1** | |
| America | Brazil | 226186400 | 64838160 | 52361724 | 117199884 | 51.8 | 104797800 | 18093600 | 122891400 | 54.3 | |
| America | United Estates | 366044900 | 24799830 | 14636370 | 39436200 | 10.8 | 23617820 | 4533182 | 28151002 | 7.7 | |
| America | Colombia | 67737790 | 22970650 | 15481367 | 38452017 | 56.8 | 15900540 | 12232100 | 28132640 | 41.5 | |
| America | Mexico | 152410700 | 13652610 | 10830574 | 24483184 | 16.1 | 28110530 | 13082960 | 41193490 | 27.0 | |
| America | Venezuela | 32392350 | 9172357 | 14066871 | 23239228 | 71.7 | 16483680 | 10471040 | 26954720 | 83.2 | |
| America | Argentina | 47360840 | 15847480 | 5808448 | 21655928 | 45.7 | 20144200 | 27898 | 20172098 | 42.6 | |
| America | Guatemala | 19430940 | 6144703 | 8424246 | 14568949 | 75.0 | 8279845 | 2818644 | 11098489 | 57.1 | |
| America | Haiti | 14326800 | 3290186 | 8828620 | 12118806 | 84.6 | 9331020 | 4551186 | 13882206 | 96.9 | |
| America | Cuba | 11748390 | 5091432 | 5400005 | 10491437 | 89.3 | 5556622 | 6014379 | 11571001 | 98.5 | |
| America | Honduras | 10390240 | 2586466 | 6922323 | 9508789 | 91.5 | 6546443 | 3349775 | 9896218 | 95.2 | |
| America | Dominican Republic | 12151280 | 1569534 | 6800846 | 8370380 | 68.9 | 5718382 | 5892294 | 11610676 | 95.6 | |
| America | El Salvador | 7724171 | 2223137 | 4174670 | 6397807 | 82.8 | 5645076 | 1259631 | 6904707 | 89.4 | |
| America | Nicaragua | 7645260 | 3233992 | 2905169 | 6139161 | 80.3 | 3844025 | 3174065 | 7018090 | 91.8 | |
| America | Canada | 37719540 | 4146128 | 950607 | 5096735 | 13.5 | 801306 | 0 | 801306 | 2.1 | |
| America | Costa Rica | 5608981 | 1123624 | 3832489 | 4956113 | 88.4 | 3411996 | 508435 | 3920431 | 69.9 | |
| America | Ecuador | 17754630 | 2832121 | 1540001 | 4372122 | 24.6 | 4747518 | 1622610 | 6370128 | 35.9 | |
| America | Bolivia | 13803170 | 2489557 | 1684106 | 4173663 | 30.2 | 3363616 | 758774 | 4122390 | 29.9 | |
| America | Chile | 20821860 | 2874216 | 438730 | 3312946 | 15.9 | 1107709 | 180117 | 1287826 | 6.2 | |
| America | Panama | 4420613 | 795531 | 2411974 | 3207505 | 72.6 | 1940434 | 1990969 | 3931403 | 88.9 | |
| America | Puerto Rico | 3813211 | 1045006 | 1418546 | 2463552 | 64.6 | 2025103 | 1202161 | 3227264 | 84.6 | |
| America | Peru | 38585900 | 1547102 | 731359 | 2278461 | 5.9 | 789369 | 0 | 789369 | 2.0 | |
| America | Jamaica | 2942278 | 513022 | 1323378 | 1836399 | 62.4 | 1092317 | 1385191 | 2477508 | 84.2 | |
| America | Paraguay | 7874698 | 167810 | 953675 | 1121485 | 14.2 | 4852130 | 663 | 4852793 | 61.6 | |
| America | Trinidad y Tobago | 1227690 | 418965 | 300906 | 719871 | 58.6 | 832874 | 125491 | 958365 | 78.1 | |
| America | Guyana | 815360 | 168085 | 366589 | 534673 | 65.6 | 312394 | 320824 | 633218 | 77.7 | |
| America | Belize | 414168 | 92544 | 296572 | 389116 | 94.0 | 185354 | 205676 | 391030 | 94.4 | |
| America | Guadalupe (France) | 403535 | 91275 | 267541 | 358816 | 88.9 | 31249 | 357412 | 388661 | 96.3 | |
| America | Martinican (France) | 423480 | 51554 | 277215 | 328769 | 77.6 | 124585 | 297982 | 422567 | 99.8 | |
| America | Barbados | 178716 | 17365 | 161351 | 178716 | 100.0 | 5403 | 173313 | 178716 | 100.0 | |
| America | Santa Lucia | 181479 | 17384 | 160358 | 177741 | 97.9 | 18570 | 162909 | 181479 | 100.0 | |
| America | Surinam | 681709 | 747 | 127294 | 128041 | 18.8 | 436840 | 27186 | 464026 | 68.1 | |
| America | Bahamas | 171796 | 42176 | 52055 | 94231 | 54.9 | 119701 | 12573 | 132274 | 77.0 | |
| America | French Guyana | 330861 | 38092 | 50747 | 88839 | 26.9 | 99705 | 41397 | 141102 | 42.6 | |
| America | Uruguay | 4121751 | 59744 | 0 | 59744 | 1.4 | 196598 | 0 | 196598 | 4.8 | |
| America | St. Vincent and the Grenadines | 78692 | 31124 | 28566 | 59690 | 75.9 | 50126 | 28566 | 78692 | 100.0 |  |
| America | Antigua y Barbuda | 58124 | 980 | 56513 | 57492 | 98.9 | 632 | 57492 | 58124 | 100.0 | |
| America | Dominica | 32545 | 9669 | 2831 | 12500 | 38.4 | 21483 | 8554 | 30037 | 92.3 | |
| America | Virgins Island (US) | 10003 | 0 | 10003 | 10003 | 100.0 | 0 | 10003 | 10003 | 100.0 | |
| America | Granada | 8403 | 6186 | 0 | 6186 | 73.6 | 8403 | 0 | 8403 | 100.0 | |
| America | San Cristobal y Nieves | 6513 | 4023 | 0 | 4023 | 61.8 | 3793 | 2720 | 6513 | 100.0 | |
| America | Virgins Island (UK) | 3534 | 0 | 3534 | 3534 | 100.0 | 0 | 3534 | 3534 | 100.0 | |
| America | Bonaire | 10887 | 0 | 0 | 0 | 0.0 | 10323 | 0 | 10323 | 94.8 | |
| America | Curacao (Netherlands) | 88882 | 0 | 0 | 0 | 0.0 | 88882 | 0 | 88882 | 100.0 | |
| America | Islas Malvinas | 2851 | 0 | 0 | 0 | 0.0 | 0 | 0 | 0 | 0.0 | |
| America | San Pedro y Miquelon (France) | 107 | 0 | 0 | 0 | 0.0 | 0 | 0 | 0 | 0.0 | |
| America | Turks y Caicos (UK) | 601 | 0 | 0 | 0 | 0.0 | 309 | 157 | 466 | 77.6 | |
|  | **Total** | **1138146630** | **194004565** | **174088171** | **368092736** | **32.3** | **280654706** | **94985462** | **375640168** | **33.0** | |
| Europe | Portugal | 10429060 | 2485807 | 1407782 | 3893589 | 37.3 | 1489585 | 0 | 1489585 | 14.3 | |
| Europe | Italy | 62912970 | 2195231 | 1099038 | 3294269 | 5.2 | 4498057 | 0 | 4498057 | 7.1 | |
| Europe | Spain | 54125480 | 1789360 | 391590 | 2180950 | 4.0 | 1315977 | 0 | 1315977 | 2.4 | |
| Europe | United Kingdom | 67997440 | 1747281 | 0 | 1747281 | 2.6 | 1341649 | 0 | 1341649 | 2.0 | |
| Europe | Switzerland | 9970213 | 1319049 | 0 | 1319049 | 13.2 | 9575 | 0 | 9575 | 0.1 | |
| Europe | France | 70983470 | 1150794 | 0 | 1150794 | 1.6 | 293082 | 0 | 293082 | 0.4 | |
| Europe | Albania | 2919457 | 1083495 | 61524 | 1145019 | 39.2 | 822825 | 0 | 822825 | 28.2 | |
| Europe | Germany | 82446910 | 433178 | 0 | 433178 | 0.5 | 5114623 | 0 | 5114623 | 6.2 | |
| Europe | Ireland | 5308876 | 329980 | 0 | 329980 | 6.2 | 211883 | 0 | 211883 | 4.0 | |
| Europe | Austria | 9390486 | 295218 | 0 | 295218 | 3.1 | 0 | 0 | 0 | 0.0 | |
| Europe | Bosnia and Herzegovina | 3583124 | 265673 | 0 | 265673 | 7.4 | 36435 | 0 | 36435 | 1.0 | |
| Europe | Turkey | 84529920 | 236028 | 0 | 236028 | 0.3 | 599306 | 16168 | 615473 | 0.7 | |
| Europe | Slovenia | 2410791 | 235270 | 0 | 235270 | 9.8 | 12218 | 0 | 12218 | 0.5 | |
| Europe | Greece | 10713270 | 78420 | 143996 | 222416 | 2.1 | 180533 | 0 | 180533 | 1.7 | |
| Europe | Montenegro | 643544 | 141071 | 6240 | 147311 | 22.9 | 93385 | 0 | 93385 | 14.5 | |
| Europe | Croatia | 4281344 | 105982 | 1148 | 107130 | 2.5 | 122720 | 0 | 122720 | 2.9 | |
| Europe | Georgia | 3960222 | 91807 | 0 | 91807 | 2.3 | 19870 | 0 | 19870 | 0.5 | |
| Europe | Island | 272808 | 44181 | 0 | 44181 | 16.2 | 0 | 0 | 0 | 0.0 | |
| Europe | Norway | 5022757 | 24239 | 0 | 24239 | 0.5 | 0 | 0 | 0 | 0.0 | |
| Europe | Poland | 42412250 | 17324 | 0 | 17324 | 0.0 | 0 | 0 | 0 | 0.0 | |
| Europe | Ukraine | 47792190 | 10082 | 0 | 10082 | 0.0 | 0 | 0 | 0 | 0.0 | |
| Europe | Andorra | 116967 | 0 | 0 | 0 | 0.0 | 0 | 0 | 0 | 0.0 | |
| Europe | Belgium | 11918820 | 0 | 0 | 0 | 0.0 | 0 | 0 | 0 | 0.0 | |
| Europe | Belarus | 9748290 | 0 | 0 | 0 | 0.0 | 0 | 0 | 0 | 0.0 | |
| Europe | Bulgaria | 7413625 | 0 | 0 | 0 | 0.0 | 0 | 0 | 0 | 0.0 | |
| Europe | Cyprus | 1309758 | 0 | 0 | 0 | 0.0 | 0 | 0 | 0 | 0.0 | |
| Europe | Denmark | 5653747 | 0 | 0 | 0 | 0.0 | 0 | 0 | 0 | 0.0 | |
| Europe | Slovakia | 5988831 | 0 | 0 | 0 | 0.0 | 0 | 0 | 0 | 0.0 | |
| Europe | Estonia | 1377564 | 0 | 0 | 0 | 0.0 | 0 | 0 | 0 | 0.0 | |
| Europe | Finland | 6042599 | 0 | 0 | 0 | 0.0 | 0 | 0 | 0 | 0.0 | |
| Europe | Guernsey (UK) | 45691 | 0 | 0 | 0 | 0.0 | 0 | 0 | 0 | 0.0 | |
| Europe | Hungary | 10315400 | 0 | 0 | 0 | 0.0 | 0 | 0 | 0 | 0.0 | |
| Europe | Man Island (UK) | 94648 | 0 | 0 | 0 | 0.0 | 0 | 0 | 0 | 0.0 | |
| Europe | Faroe Island (Denmark) | 41224 | 0 | 0 | 0 | 0.0 | 29579 | 0 | 29579 | 71.8 | |
| Europe | Jersey (UK) | 64664 | 0 | 0 | 0 | 0.0 | 0 | 0 | 0 | 0.0 | |
| Europe | Leetonia | 2697613 | 0 | 0 | 0 | 0.0 | 0 | 0 | 0 | 0.0 | |
| Europe | Liechtenstein | 43730 | 0 | 0 | 0 | 0.0 | 0 | 0 | 0 | 0.0 | |
| Europe | Lithuania | 2976386 | 0 | 0 | 0 | 0.0 | 0 | 0 | 0 | 0.0 | |
| Europe | Luxemburg | 675724 | 0 | 0 | 0 | 0.0 | 0 | 0 | 0 | 0.0 | |
| Europe | Macedonia | 2427468 | 0 | 0 | 0 | 0.0 | 0 | 0 | 0 | 0.0 | |
| Europe | Malta | 249558 | 0 | 0 | 0 | 0.0 | 0 | 0 | 0 | 0.0 | |
| Europe | Moldova | 4049481 | 0 | 0 | 0 | 0.0 | 0 | 0 | 0 | 0.0 | |
| Europe | Netherlands | 18190420 | 0 | 0 | 0 | 0.0 | 0 | 0 | 0 | 0.0 | |
| Europe | Czech Republic | 11919980 | 0 | 0 | 0 | 0.0 | 0 | 0 | 0 | 0.0 | |
| Europe | Romania | 35761950 | 0 | 0 | 0 | 0.0 | 0 | 0 | 0 | 0.0 | |
| Europe | San Marino | 30361 | 0 | 0 | 0 | 0.0 | 0 | 0 | 0 | 0.0 | |
| Europe | Serbia | 9817854 | 0 | 0 | 0 | 0.0 | 0 | 0 | 0 | 0.0 | |
| Europe | Sweden | 10073670 | 0 | 0 | 0 | 0.0 | 0 | 0 | 0 | 0.0 | |
|  | **Total** | **741152604** | **14079470** | **3111318** | **17190788** | **2.3** | **16191299** | **16168** | **16207467** | **2.2** | |
| Oceania | Australia | 22629980 | 2286780 | 3473679 | 5760459 | 25.5 | 6756227 | 372480 | 7128707 | 31.5 | |
| Oceania | Papua New Guinea | 9557455 | 1073417 | 743551 | 1816968 | 19.0 | 1557287 | 566724 | 2124011 | 22.2 | |
| Oceania | New Zeeland | 3730494 | 300330 | 0 | 300330 | 8.1 | 184494 | 0 | 184494 | 4.9 | |
| Oceania | Salomon Island | 534271 | 131180 | 112999 | 244178 | 45.7 | 268615 | 103821 | 372435 | 69.7 | |
| Oceania | Fiji | 663878 | 132296 | 56675 | 188971 | 28.5 | 443699 | 51261 | 494959 | 74.6 | |
| Oceania | New Caledonia (France) | 156479 | 33215 | 32394 | 65609 | 41.9 | 55860 | 334 | 56195 | 35.9 | |
| Oceania | Vanuatu | 223111 | 7701 | 19332 | 27033 | 12.1 | 69660 | 909 | 70569 | 31.6 | |
|  | **Total** | **37495667** | **3964918** | **4438630** | **8403548** | **22.4** | **9335842** | **1095529** | **10431370** | **27.8** | |
| **World Wide** | **Total** | **8443323187** | **1339920835** | **1339991724** | **2679912559** | **31.7** | **2104440338** | **1344334868** | **3448775207** | **40.8** | |
